# Point-of-Care C-Reactive Protein Testing to Reduce Antibiotic Prescribing for Respiratory Tract Infections in Primary Care: Systematic Review and Meta-Analysis of Randomised Controlled Trials

**DOI:** 10.3390/antibiotics9090610

**Published:** 2020-09-16

**Authors:** Nahara Anani Martínez-González, Ellen Keizer, Andreas Plate, Samuel Coenen, Fabio Valeri, Jan Yvan Jos Verbakel, Thomas Rosemann, Stefan Neuner-Jehle, Oliver Senn

**Affiliations:** 1 Institute of Primary Care, University of Zurich and University Hospital of Zurich, Pestalozzistrasse 24, CH-8091 Zurich, Switzerland; ellen.keizer@usz.ch (E.K.); andreas.plate@usz.ch (A.P.); fabio.valeri@usz.ch (F.V.); thomas.rosemann@usz.ch (T.R.); stefan.neuner-jehle@usz.ch (S.N.-J.); oliver.senn@usz.ch (O.S.); 2 Department of Health Sciences and Medicine, University of Lucerne, Frohburgstrasse 3, PO Box 4466, CH-6002 Lucerne, Switzerland; 3 Centre for General Practice, Department of Family Medicine & Population Health (FAMPOP), University of Antwerp-Campus Drie Eiken, Doornstraat 331, 2610 Antwerp (Wilrijk), Belgium; samuel.coenen@uantwerpen.be; 4 Laboratory of Medical Microbiology, Vaccine & Infectious Disease Institute (VAXINFECTIO), University of Antwerp-Campus Drie Eiken, Universiteitsplein 1, 2610 Antwerp (Wilrijk), Belgium; 5 EPI-Centre, Department of Public Health and Primary Care, KU Leuven (University of Leuven), Kapucijnenvoer 33, 3000 Leuven, Belgium; jan.verbakel@kuleuven.be; 6 Nuffield Department of Primary Care Health Sciences, NIHR Community Healthcare MIC, University of Oxford, Radcliffe Primary Care Building, Radcliffe Observatory Quarter, Woodstock Road, Oxford OX2 6GG, UK

**Keywords:** antibiotic stewardship, antibiotic prescribing, antibiotic use, point-of-care testing, c-reactive protein, diagnostics, respiratory tract infection, primary care, systematic review, meta-analysis

## Abstract

C-reactive protein (CRP) point-of-care testing (POCT) is increasingly being promoted to reduce diagnostic uncertainty and enhance antibiotic stewardship. In primary care, respiratory tract infections (RTIs) are the most common reason for inappropriate antibiotic prescribing, which is a major driver for antibiotic resistance. We systematically reviewed the available evidence on the impact of CRP-POCT on antibiotic prescribing for RTIs in primary care. Thirteen moderate to high-quality studies comprising 9844 participants met our inclusion criteria. Meta-analyses showed that CRP-POCT significantly reduced immediate antibiotic prescribing at the index consultation compared with usual care (RR 0.79, 95%CI 0.70 to 0.90, *p* = 0.0003, I^2^ = 76%) but not during 28-day (*n* = 7) follow-up. The immediate effect was sustained at 12 months (*n* = 1). In children, CRP-POCT reduced antibiotic prescribing when CRP (cut-off) guidance was provided (*n* = 2). Meta-analyses showed significantly higher rates of re-consultation within 30 days (*n* = 8, 1 significant). Clinical recovery, resolution of symptoms, and hospital admissions were not significantly different between CRP-POCT and usual care. CRP-POCT can reduce immediate antibiotic prescribing for RTIs in primary care (number needed to (NNT) for benefit = 8) at the expense of increased re-consultations (NNT for harm = 27). The increase in re-consultations and longer-term effects of CRP-POCT need further evaluation. Overall, the benefits of CRP-POCT outweigh the potential harms (NNTnet = 11).

## 1. Introduction

Acute respiratory tract infections (RTIs) are among the most common reasons for patient encounters in primary care and for inappropriate antibiotic prescribing, which is a major driver for antibiotic resistance (ABR) [1,2,3,4,5]. RTIs are predominantly of viral aetiology and self-limiting in most otherwise healthy individuals. Evidence from systematic reviews and other studies shows that most patients suffering from acute RTIs, such as sore throat, acute sinusitis, pharyngitis, rhinosinusitis, otitis media, and acute bronchitis, do not benefit from antibiotic therapy [6,7,8]. International clinical practice guidelines consequently advise against routine treatment of uncomplicated RTIs with antibiotics [9]. The vast majority of patients with these infections, however, receive an antibiotic prescription for systemic use after seeking medical attention from their primary care physician [4,10].

Inappropriate use of antibiotics is linked to the development of drug-resistant bacteria and increases the incidence of adverse events, re-consultations, and complications and subsequently increases healthcare costs [3,11,12,13,14,15]. Moreover, the rates of antibiotic prescribing have been directly associated with the rates of ABR at the individual, community, and national levels [3,4]. The reduction of antibiotic prescribing for acute RTIs could thus help to decrease ABR. If no effective actions are taken ABR could become the leading cause of death, surpassing cancer [16].

C-reactive protein (CRP) point-of-care test(-ing) (POCT) is one of the top strategies targeted at clinicians to reduce antibiotic prescribing, and it is increasingly being promoted to enhance antibiotic stewardship [17]. It has been demonstrated that uncertainty about the diagnosis of infection can lead to inappropriate antibiotic prescribing, overuse of resources, and disease complications [18,19,20]. With CRP as a biomarker of systematic inflammation, however, CRP-POCT enables clinicians to discern bacterial infections from other inflammatory disorders and helps them to identify the patients who benefit the most from antibiotics [21]. The robustness and accuracy of CRP-POCT compared with laboratory testing have been demonstrated by diagnostic studies [22]. CRP-POCT has also been integrated into some clinical guidelines as part of the assessment for RTIs to reduce diagnostic uncertainty and to aid prescribing decisions [23,24].

Two systematic reviews have suggested that CRP-POCT provided with prescribing guidance can reduce antibiotic prescribing for acute infections. One review focused on RTIs in primary care, but the results published six years ago were limited by a small number of studies of generally small samples that included mostly adults [25]. The second review with a broader scope included all acute infections presenting to ambulatory care [26]. We performed a systematic review to assess the evidence on the clinical effectiveness of CRP-POCT compared with usual care in reducing antibiotic prescribing for RTIs in primary care.

## 2. Results

### 2.1. Identification of Studies

**Figure 1:**
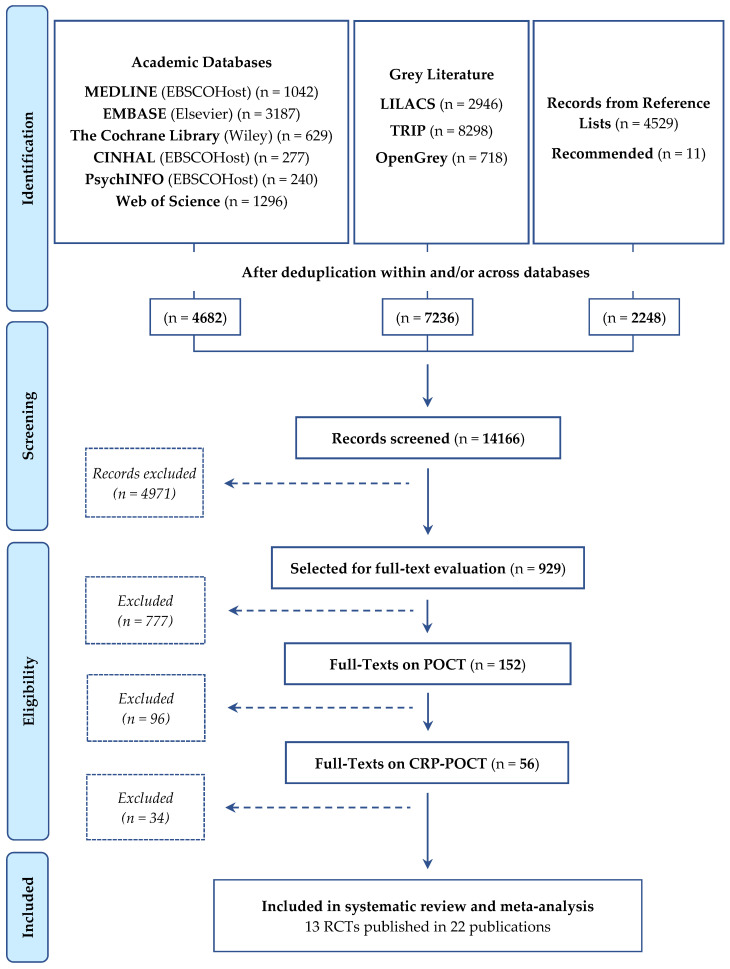
Study identification and process for selection of studies included in the review.

Our searches identified 14,166 records. After full-text evaluation, 152 publications were relevant for POCT. After excluding 34 publications [27,28,29,30,31,32,33,34,35,36,37,38,39,40,41,42,43,44,45,46,47,48,49,50,51,52,53,54,55,56,57,58,59,60] (Supplementary Materials), 13 studies [61,62,63,64,65,66,67,68,69,70,71,72,73] reported across 22 publications [61,62,63,64,65,66,67,68,69,70,71,72,73,74,75,76,77,78,79,80,81,82] met the inclusion criteria for CRP-POCT. Of those, 11 were published in English, one in Norwegian and one was published in both English and Danish. Figure 1 shows the flow of study identification and selection.

### 2.2. Study and Population Characteristics

The studies were published from 1995 to 2019, with 10 studies in the last 10 years (2009–2019) (Table 1). Nine studies were conducted in eight European countries, mainly the Netherlands; four other studies were from Russia, the USA, Japan, and Vietnam. The studies were mostly carried out in the general practitioner’s (GP) office (*n* = 7) and outpatient (primary care centres and hospital) services (*n* = 2). The type of RTI was anatomically defined as upper and/or lower in only six studies. RTI without specific signs or symptoms (*n* = 7) was the most common reason for encounter.

The 13 studies comprised a total of 9844 randomised patients (Table 1); nine individually randomised 4529 patients to intervention groups [61,63,64,65,68,69,71,72,73], and four randomised 226 sites and/or 173 physicians with 5315 patients [62,66,67,70]. Six studies included adult patients only [66,67,68,69,70,73], four included children only [61,62,63,64], two included both adults and children [65,72], and one did not distinguish age groups [71]. Participants had a mean age of 26.4 (SD 15.0) (range: 0–90) years, and 43.9% were male. The ethnicity of patients was reported in only one study as follows: black (46%), white (25%), or other (29%) [68]. Physician characteristics were reported in two studies [70,71]: they were 29–53 years old; had 5–29 years of experience; and in one study, 60% were male [70].

### 2.3. Intervention Characteristics

CRP-POCT was performed by physicians [61,62,63,65,66,67,70,71,72,73] or nurses [64,68,69] (Table 1). Nine studies reported training in the intervention, which consisted of instructions on CRP devices, guidelines for the use of CRP, and/or management of RTIs [62,64,65,66,67,68,69,70,72]. In 11 studies, clinicians also received guidance or information for the interpretation of CRP results in terms of disease severity, antibiotic prescribing, or both. Only eight of these additionally provided specific CRP cut-off guidance to prescribe or to withhold antibiotics [62,65,66,67,68,69,70,73]. Studies used four types of CRP quantitative devices that comprised test kits and analysers, including NycoCard (II) CRP readers, QuickRead CRP kits, Afinion CRP, or CRP multichannel analyser. All studies compared CRP-POCT with a control intervention consisting of usual care. One study used a 2 × 2 factorial design and combined intervention groups, including the effect of enhanced communication skills training (CST), to both CRP-POCT and usual care [70]. Usual care was described as a clinical assessment as usual with CRP not tested [61,63,64,72,73] or as routine practice according to normal procedures with/out access to other investigations [65,68,69,70,71,78]. However, CRP-POCT could have also been performed if patients in the usual care group were at clinical risk or if the results of physical examinations suggested it to be necessary [62,66].

### 2.4. Methodological Quality and Risk of Bias in the Methods of Included Studies

All studies reported the participants’ inclusion criteria and only ten reported the exclusion criteria (Table 2). While most studies (86%) measured the success of interventions by definition of a primary outcome, a majority (64%) also defined secondary outcomes. Most studies (93%) reported sample size calculation and power. Four studies were funded, partly funded, or the reagents were provided by the manufacturers of the CRP-POCT devices [61,65,69,73].

Domains of internal validity mostly showed a low or unclear risk of bias (Table 2 and Supplementary Materials). Most (79%) studies adequately randomised participants, but it was often (64%) unclear how allocation concealment was ensured in the randomisation process. As expected, considering the focus of our systematic review on clinical effectiveness, all studies were at high risk of performance bias as blinding of clinicians and patients to group allocation was not possible. Delivery of the intervention was dependent on both a patient POCT procedure and knowing the results of CRP to treat patients. Most studies adequately performed blinding of outcome assessors for both the primary (64%) and secondary (57%) outcomes, although only five reported the outcome assessors [65,68,70,73,80]. In all studies, primary outcome data were complete and had an acceptable level of attrition (≤20%). The length of follow-up was the same for all groups in each study (range: 0 days to 3.5 years).

### 2.5. Effectiveness of the Use of CRP-POCT on (patient) Outcomes

All 13 studies reported on the primary outcome of rates of antibiotic prescribing. Secondary outcomes were variably reported across studies, limiting the ability to conduct meta-analyses in some cases. The Supplementary Materials show the individual effect estimates not represented in forest plots and all additional figures from meta-analyses.

#### 2.5.1. Primary Outcomes

##### Antibiotic Prescribing Rate at the Index Consultation

**Figure 2:**
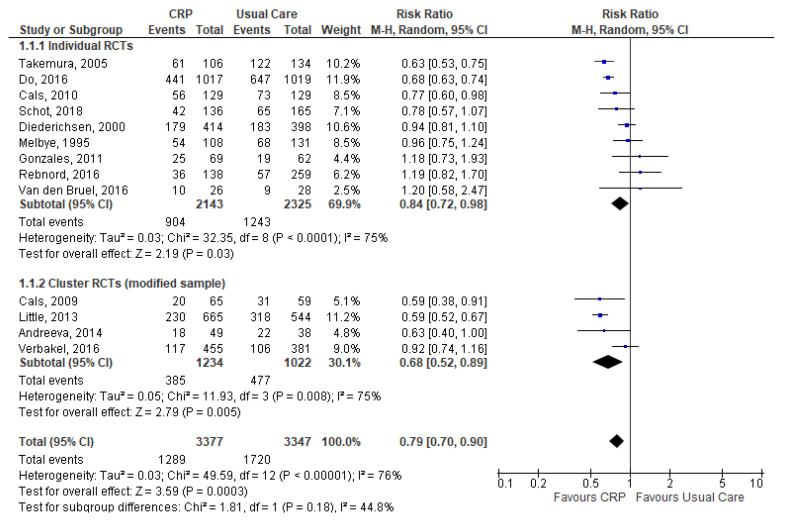
Comparison: CRP-POCT versus usual care. Overall antibiotic prescribing at index consultations. CRP, C-Reactive Protein Point Of Care Test; RCTs, Randomised Controlled Trials; M-H, Mantel-Haenszel; CI, Confidence Interval; df, degrees of freedom; I^2^, heterogeneity between trials.

Meta-analysis of all 13 studies demonstrated significantly lower antibiotic prescribing in the CRP-POCT group compared with the usual care group (38.2% vs. 51.4%: RR 0.79, 95%CI 0.70 to 0.90, *p* = 0.0003) (Figure 2). Between-study heterogeneity was considerable (I^2^ = 76%).

##### Subgroup Analyses of the Antibiotic Prescribing Rate at the Index Consultation

**Figure 3:**
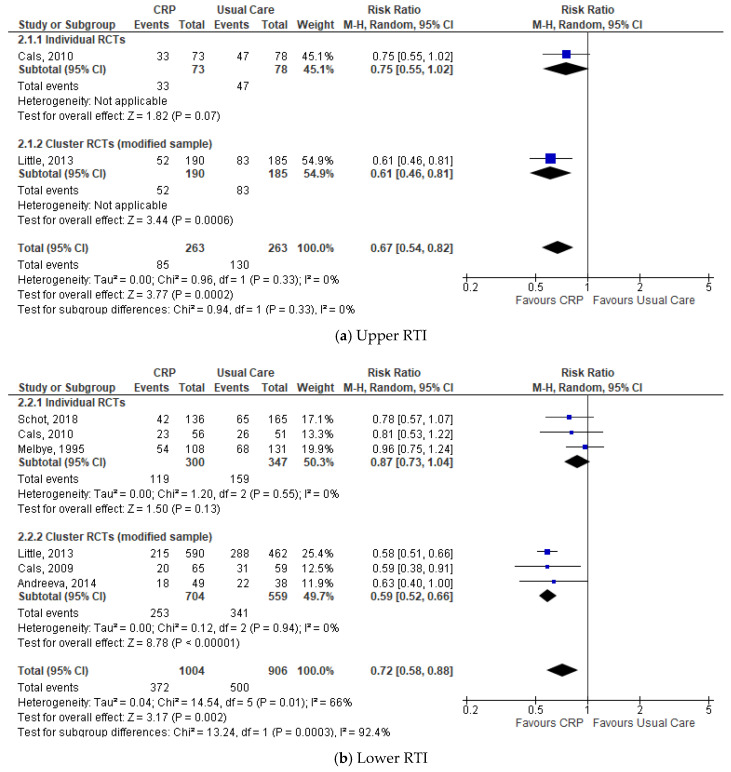
Comparison: CRP-POCT versus usual care. Antibiotic prescribing at index consultations by type of RTI: (**a**) Upper RTI; (**b**) Lower RTI. CRP, C-Reactive Protein Point Of Care Test; RCTs, Randomised Controlled Trials; M-H, Mantel-Haenszel; CI, Confidence Interval; df, degrees of freedom; I^2^, heterogeneity between trials.

Subgroup analysis by type of RTI showed significantly lower antibiotic prescribing in the CRP-POCT group for URTI and for LRTI (URTI (*n* = 2), 32.3% vs. 49.4%: RR 0.67, 95%CI 0.54 to 0.82, *p* = 0.0002, I^2^ = 0%; LRTI (*n* = 6), 37.1% vs. 55.2%: RR 0.72, 95%CI 0.58 to 0.88, *p* = 0.002, I^2^ = 66%) (Figure 3). The pooled estimate showed no significant differences between groups from seven studies with undefined RTI, e.g., signs and symptoms of infection (Supplementary Materials).

**Figure 4:**
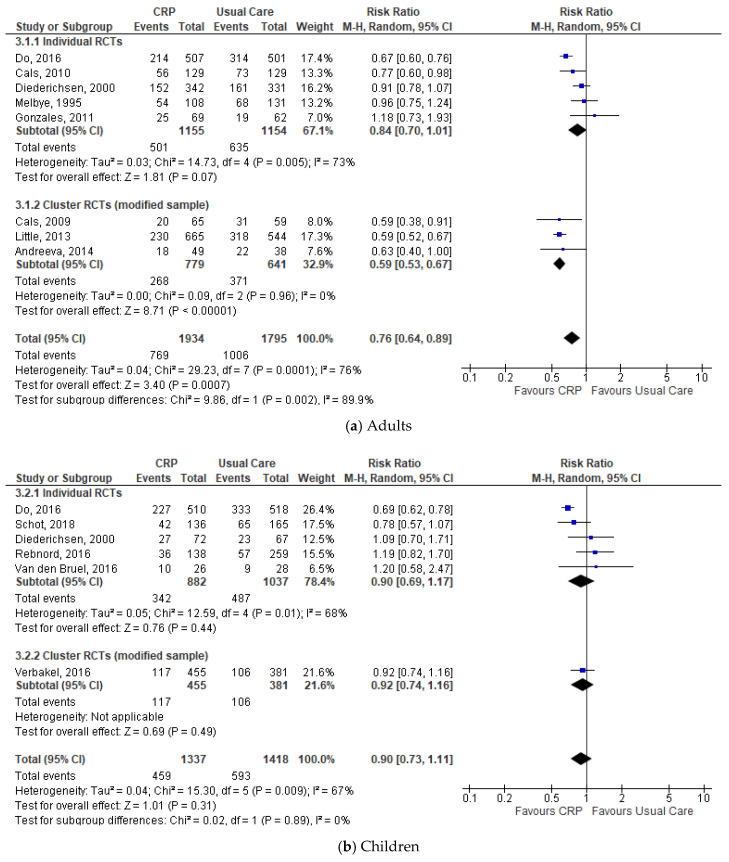
Comparison: CRP-POCT versus usual care. Antibiotic prescribing at index consultations by age group: (**a**) Adults; (**b**) Children. CRP, C-Reactive Protein Point Of Care Test; RCTs, Randomised Controlled Trials; M-H, Mantel-Haenszel; CI, Confidence Interval; df, degrees of freedom; I^2^, heterogeneity between trials.

Subgroup analysis by age groups also showed significantly lower antibiotic prescribing for adults and not significantly lower antibiotic prescribing for children in the CRP-POCT group (≥18 years (*n* = 8), 39.8% vs. 56.0%: RR 0.76, 95%CI 0.64 to 0.89, *p* = 0.0007, I^2^ = 76%; <18 years (*n* = 6), 34.3% vs. 41.8%: RR 0.90, 95%CI 0.73 to 1.11, *p* = 0.31, I^2^ = 67%) (Figure 4).

Subgroup analysis showed significantly lower antibiotic prescribing when CRP cut-off guidance was applied to withhold antibiotics (*n* = 8, 37.6% vs. 54.4%: RR 0.75, 95%CI 0.65 to 0.87, *p* = 0.0001, I^2^ = 72%) and not significantly lower antibiotic prescribing when CRP guidance in five studies was not clear or not applied to withhold or initiate antibiotics (Supplementary Materials).

**Figure 5:**
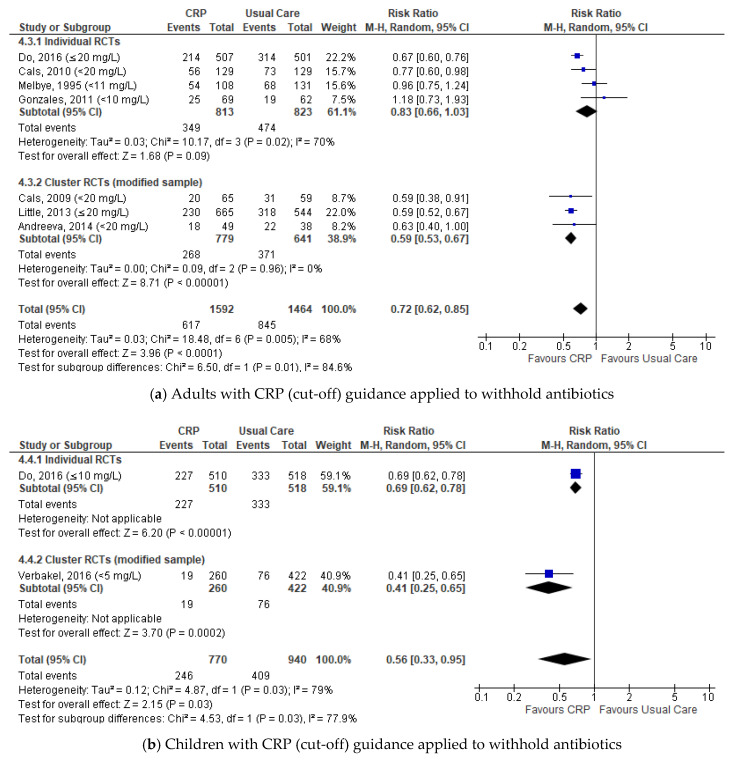
Comparison: CRP-POCT versus usual care. Antibiotic prescribing at index consultations by age group with CRP (cut-off) guidance to withhold antibiotics: (**a**) Adults; (**b**) Children. CRP values recommended to withhold antibiotics are presented within brackets. CRP, C-Reactive Protein (Point Of Care Test); RCTs, Randomised Controlled Trials; M-H, Mantel-Haenszel; CI, Confidence Interval; df, degrees of freedom; I^2^, heterogeneity between trials.

Subgroup analysis by CRP guidance and age groups demonstrated significantly lower antibiotic prescribing among adults in whom clinical CRP cut-off guidance was applied to withhold antibiotics (≥18 years (*n* = 8), 38.8% vs. 54.4%: RR 0.72, 95%CI 0.62 to 0.85, *p* < 0.0001, I^2^ = 68%) (Figure 5). The pooled estimate also showed significantly lower antibiotic prescribing among children in whom CRP cut-off guidance was applied to withhold antibiotics (<18 years (*n* = 2), 31.9% vs. 43.5%: RR 0.56, 95%CI 0.33 to 0.95, *p* = 0.03, I^2^ = 79%).

Subgroup analysis by clinical settings showed significantly lower antibiotic prescribing among patients in the CRP group who attended GP practices (*n* = 7, 35.8% vs. 47.7%: RR 0.77, 95%CI 0.64 to 0.94, *p* = 0.009, I^2^ = 81%) or outpatient (hospital and healthcare centres) services (*n* = 2, 44.7% vs. 66.7%: RR 0.67, 95%CI 0.62 to 0.73, *p* < 0.00001, I^2^ = 0%) but not in other settings from five studies (Supplementary Materials).

Overall between-study heterogeneity was not significant in the subgroup of URTI (I^2^ = 0%, *n* = 2), clinical settings other than GPs (I^2^ = 0–3%, *n* = 2–5 studies) and the set of cluster RCTs (I^2^ = 0%, *n* = 1–3 studies) for LRTI, adults, and adults with CRP (cut-off) guidance. In all other subgroups, heterogeneity remained substantial to considerable.

##### Antibiotic Prescribing Rate during Patient Follow-Up and Longer-Term Effects

Meta-analysis of seven studies demonstrated no significant difference between CRP-POCT and usual care in the rates of antibiotic prescribing at any point during 28 days of follow-up, with moderate between-study heterogeneity (36.3% vs. 41.7%: RR 0.85, 95%CI 0.72 to 1.02, *p* = 0.08, I^2^ = 48%). The individual effect estimate of one study showed significantly lower antibiotic prescribing at 12 months in the same GP practice (43.4% vs. 51.4%: RR 0.84, 95%CI 0.77 to 0.92, *p* = 0.0002) [67,78]. Individual effect estimates of two studies showed a non-significant trend towards lower antibiotic prescribing in the CRP-POCT group at 3 months [61] or 3.5 years [70] of testing.

#### 2.5.2. Sensitivity Analysis and Meta-Regression

The pooled estimate in the meta-analysis of antibiotic prescribing at index consultation was not significantly affected by systematically excluding any study with clinical or methodological variation; heterogeneity remained substantial (Supplementary Materials). The pooled estimate slightly reduced in some cases, but the direction of effect remained significant towards improvement with CRP-POCT. Analyses by continents showed the effect remained significant in favour of CRP-POCT.

Using meta-regression, 59.0% of the between-study heterogeneity (I^2^ = 74.4%, *p* < 0.001) could be explained by three modifiers (I^2^ = 47.9%, *p* = 0.032), including the year of publication (RR 0.86, 95%CI 0.75 to 0.97, *p* = 0.014), age (children: RR 1.21, 95%CI 1.02 to 1.43, *p* = 0.03), and CRP cut-off guidance (RR 0.86, 95%CI 0.71 to 1.04, *p* = 0.11) (Supplementary Materials).

#### 2.5.3. Secondary Outcomes

##### Clinical Recovery and Resolution of Symptoms

Meta-analyses of three studies demonstrated no significant difference between CRP-POCT and usual care in the number of patients with clinical recovery at 7 and 28 days, with no heterogeneity between trials (7 days, 51.7% vs. 52.8%: RR 1.03, 95%CI 0.93 to 1.14, *p* = 0.53, I^2^ = 0%; 28 days, 77.8% vs. 75.3%: RR 0.95, 95%CI 0.70 to 1.28, *p* = 0.72, I^2^ = 0%). Individual estimates of two studies also showed no significant differences between CRP-POCT and usual care in clinical recovery at 7 days [69], in the time (number of days) to full clinical recovery at 28 days [69], or in the mean symptom severity scores [67]. Individual estimates of one study showed significantly longer-time (mean number of days) to the resolution of symptoms among patients in the CRP-POCT group compared with usual care (MD 0.33, 95%CI 0.03 to 0.63, *p* = 0.03) [67]. This difference, however, was not significant between CRP-POCT and usual care in another study [65].

##### Visit Duration and Visits at Follow-Up

Individual estimates of one study showed no significant differences between CRP-POCT and usual care in the length of patients’ visits [68]. One trial showed a reduction in the number of patients with RTI who visited their physician within 3.5 years of follow-up, which started after the first 28 days [70].

##### Re-Consultations and Intention to Re-Consult

Meta-analysis of eight studies demonstrated a significant increase in re-consultations among patients in the CRP-POCT group with low between-study heterogeneity (13.5% vs. 9.7%: RR 1.33, 95%CI 1.14 to 1.57, *p* = 0.0004, I^2^ = 3%). Individual estimates of one study showed significantly more patients in the CRP-POCT group had the intention to re-consult for future similar symptoms (74.8% vs. 61.0%: RR 1.23, 95%CI 1.03 to 1.46, *p* = 0.02) [69]. This difference, however, was not significant between CRP-POCT and usual care in another study [70].

##### Referrals to Secondary Care

Meta-analysis of three studies demonstrated no significant difference between CRP-POCT and usual care in the number of patients referred to hospital with low between-study heterogeneity (2.9% vs. 4.5%: RR 0.84, 95%CI 0.44 to 1.61, *p* = 0.60, I^2^ = 18%). Individual estimates of one study also showed no significant differences between CRP-POCT and usual care in the number of patients with non-urgent referrals to secondary care (specialists or emergency department (ED)) at the time of re-consultations, or at 3 months [61].

##### Admissions to Hospital and Mortality

Meta-analysis of five studies demonstrated no significant difference in hospital admissions between CRP-POCT and usual care with no between-study heterogeneity (1.2% vs. 0.92%: RR 1.18, 95%CI 0.65 to 2.13, *p* = 0.59, I^2^ = 0%). Individual estimates of one study also showed no significant differences between CRP-POCT and usual care in hospital admissions as adverse events due to RTI at 3.5 years [70]. One study found no deaths in the CRP-POCT or usual care groups [69].

##### Ordering of Investigations

Individual estimates of two studies showed no significant difference between CRP-POCT and usual care in the number of patients for whom additional tests were ordered [62,63]. Individual estimates of one study showed significantly less patients in the CRP-POCT group for whom chest X-rays were ordered (55.5% vs. 75.7%: RR 0.73, 95%CI 0.59 to 0.91, *p* = 0.005) [66]. This difference, however, was not significant between CRP-POCT and usual care in another study [68].

##### Patient Satisfaction and Patient Enablement

Meta-analysis of three studies showed no significant difference in the number of patients who were satisfied with their medical consultations in the CRP-POCT group compared with usual care (88.6% vs. 86.8%: RR 0.82, 95%CI 0.55 to 1.21, *p* = 0.31, I^2^ = 48%). Between-study heterogeneity was moderate. Individual estimates of two studies showed no significant difference in the score of the patient enablement index among patients in the CRP-POCT group compared with usual care [69,70].

#### 2.5.4. Combined Benefit and Harm Effect of CRP-POCT

Exposure to usual care including CRP-POCT compared with usual care alone would result in an absolute risk reduction of 13.2% in antibiotic prescribing (38.2% vs. 51.4%) at index consultations (NNTB = 8). Exposure to usual care including CRP-POCT would also result in an absolute risk increase of 3.8% in re-consultations (13.5% vs. 9.7%) (NNTH = 27). Considering both the reduction in antibiotic prescribing at index consultations and the increase in re-consultations, the NNTnet of usual care including CRP-POCT is 11. That is, on average, for every 11 patients getting usual care including CRP-POCT, one patient will experience net benefit, i.e., treatment without antibiotics.

#### 2.5.5. Publication Bias

We were able to assess publication bias for the primary outcome of antibiotic prescribing at index consultations. The funnel plot was symmetrical, and the Egger’s statistic confirmed no evidence of positive publication bias (*p* = 0.092) (Supplementary Materials). There were too few studies (range, n: 2–8) to assess publication bias for other outcomes.

## 3. Discussion

In this systematic review and meta-analysis, we comprehensively summarised the available evidence from 13 RCTs on the clinical effectiveness of CRP-POCT with usual care in reducing antibiotic prescribing for RTIs in primary care. Based on evidence of moderate to high- quality, our results demonstrate that CRP-POCT can reduce immediate (at index consultations) antibiotic prescribing in patients presenting to primary care with upper and lower RTIs. CRP-POCT in combination with CRP (cut-off) guidance effectively reduced immediate antibiotic prescribing in children and enhanced the effect already gained by performing CRP-POCT in adults. Although there was a significant increase in the rates of re-consultation (NNTH = 27) within 30 days of testing, an NNTnet of 11 indicates that the benefit in reducing antibiotic prescribing (NNTB = 8) outweighs the harm when adding CRP-POCT to usual care. Most of the evidence originates from the GP setting where, in addition to the (hospital) outpatient care setting, CRP-POCT appears most effective in reducing antibiotic prescribing. We found no significant effect of CRP-POCT in the rates of clinical recovery, resolution of symptoms, hospital admissions, referrals to secondary care, or in the ordering of further investigations. One study reported no deaths. Limited evidence showed that CRP-POCT did not significantly reduce antibiotic prescribing at any point during 28-days of follow-up (*n* = 7) and that antibiotic prescribing at the index consultation was still lower in the GPs intervention at 12 months (*n* = 1).

CRP-POCT is a simple test that is being widely used in many countries to help clinicians identify whether patients with RTIs need antibiotics and to consequently reduce unnecessary antibiotic prescribing. The test can be performed within five minutes, helping clinicians with rapid decision-making during the consultation. Considering the global importance of antimicrobial resistance and its association with the extent of antibiotic consumption, compared with previous reviews [25,26], we found a slowly increasing volume of evidence from empirical research (*n* = 13 RCTs) mostly published in English. The studies were carried out mostly in high-income countries, predominantly in general practices in the northern European setting, mainly the Netherlands. The evidence represents populations of all ages (range: 0-90), the majority of whom were young adults (mean age: 26.4, SD 15.0), mostly women (56%), with signs and symptoms of RTIs. The results from this review are based on published data and should be considered within this context.

### 3.1. Strengths and Limitations of this Review

Our systematic review complements and expands on previous evaluations with a similar scope. As there is a continued relevance of the topic and findings, new studies have been published since. Our review benefits from adhering to rigorous systematic review methodology. It was based on clearly defined inclusion criteria and critical appraisal of the evidence. We restricted our review to the inclusion of RCTs only since these allow the estimation of causal effects with a lower risk of bias. Where possible, we used meta-analyses to assess the impact of CRP-POCT and to synthesize the evidence. We also carefully addressed the heterogeneity of the study results with predefined and plausible sources of heterogeneity using meta-regression analyses.

In particular, our review benefits from the inclusion of international literature published not only in English and without restriction to countries, nor to the type of health care professionals who prescribed or dispensed antibiotics, or to the year of publication. We made considerable efforts to identify all relevant studies by applying comprehensive and exhaustive literature searches in several peer-reviewed and grey literature sources between 2017 and 2019. We also conducted manual searches and received feedback from experts and colleagues (up to May 2020) about potentially relevant studies for the review, thus increasing the likelihood of identifying more contemporary literature.

The results of our systematic review are limited by the slowly increasing volume of the available literature and by the large and heterogeneous group of outcomes that resulted from our comprehensive approach. Some outcomes were variably reported across studies and in some cases, there was a dearth of data, e.g., mortality, or there was no more than one study per outcome affecting the ability to aggregate data in meta-analyses. A paucity of studies without the use of CRP guidance in children and a lack of data from older adults also limited the conclusions of our review.

### 3.2. Unanswered Questions and Future Research

The majority of participants were young adults (mean age: 26.4, SD 15.0), with merely four studies carried out exclusively in the paediatric population. Moreover, our subgroup and meta-regression analyses revealed that between-study heterogeneity can be explained by age, year of publication, and CRP guidance. In line with a previous review, CRP guidance showed the same direction in explaining heterogeneity [26], although in our analyses this was a non-significant source of heterogeneity likely due to low power. Only eight studies reported on the use of CRP (cut-off) guidance: six in adults, one in children, and one included both adults and children. Moreover, our analyses showed more pronounced effects when a more restrictive CRP (cut-off) guidance to withhold antibiotics was applied. This suggests the need for more studies not only in children in whom CRP (cut-off) guidance is applied but also in older adults, who often present a wide range of comorbidities, are generally under multiple medications, and for whom CRP cut-off values and guidance may differ from other age groups. Studies could also add to the body of evidence by reporting results by age groups and CRP guidance. Newly planned [83] and ongoing [84] trials may help to answer these questions.

The potential effect of CRP-POCT on increasing re-consultations merits consideration in future research. This result was mainly shaped by one study in a meta-analysis. Delayed prescribing was recommended as an option when CRP values were intermediate (20–100 mg/L) and if illness severity did not require immediate antibiotics. Whether these factors or illness deterioration prompt physicians to request subsequent visits or influence patient awareness for the need of reassurance about their illness should be further examined. Intermediate CRP levels may be more difficult to interpret and may benefit from further guidance [22]. GPs have shown to comply with professional guidelines in up to 70% of their management decisions [85], are generally positive about CRP-POCT, and have expressed both the need of proper indication (cut-offs) to interpret POCT and skills training to safely use POCT [86]. Depending on the healthcare system and the available financial structures (e.g., fee-for-service, budget consideration on the physician side), an increase in re-consultations might also be a barrier for CRP-POCT’s implementation in routine clinical practice.

Furthermore, RTIs were not categorised based on their anatomic involvement in 54% of the studies, limiting an aggregated analysis of their effect by RTI type, and only two studies contributed to the effect of URTIs. Although it was possible to look at the effect of clinical setting, there were more types of ‘other’ settings than the number of studies contributing to the effect per setting to produce a meaningful comparison with GPs. In addition, our subgroup analyses showed that heterogeneity may also be explained by the type of RTI and healthcare setting. Using a more standard classification for RTIs and stratifying by clinical setting would be useful to further explore and clarify the effects of CRP-POCT.

Another key question is whether the effect of CRP-POCT sustains over a long-time span. Finding a significant effect at 12 months (*n* = 1) but not at 28 days (*n* = 7) or other follow-up episodes (3 months and 3.5 years) was supported by a few studies (range, n: 1–7), half of which comprised small samples. It was also unclear if or how CRP-POCT was used over a longer-term. Although the short-term effect of CRP-POCT in our review is about the treatment of RTIs and not about its effect on patients’ and physicians’ behaviour after initial visits, long-term studies in larger populations could clarify the use of CRP-POCT and this effect.

Finally, the quality of the evidence was moderate to high due to performance bias, which is unlikely to change in future studies due to the inherent nature of the intervention. A sham control would be difficult to implement to blind control-group participants with a procedure mirroring CRP-POCT. Pragmatic (open-label) trials, however, have the advantage of producing evidence and estimates that are closer to real-life and more valuable for health economics analyses.

### 3.3. Findings in Context with other Interventions and Reviews

CST for physicians has shown a similar effect in lowering antibiotic prescribing when individually compared with CRP-POCT or usual care [67]. In our review, including one study with a factorial intervention consisting of CST added to CRP-POCT enhanced the overall effect in reducing antibiotic prescribing [70]. The study sample was small and the overall pooled effect changed by a small amount but remained significant after removing the study. Another study also reporting data (not used in our review) on similar factorial interventions showed that CST added to CRP-POCT not only enhanced the overall effect but this sustained at 12 months, and the rate of re-consultation was not significant between groups [67]. This suggests that the combined effect of CST and CRP-POCT may have a positive impact on patient behaviour, self-awareness, and management of RTIs. CST has also shown a sustainable effect [78] and appears more cost-effective than CRP-POCT alone [27]. Delayed prescribing has also been shown to reduce antibiotic use [87] and has been integrated into the National Institute for Health and Care Excellence (NICE) guidelines to aid decision-making when CRP levels are intermediate [24].

Procalcitonin has also been tested as a relevant inflammatory marker to reduce antibiotic prescribing for acute RTIs although it has no added value to signs and symptoms in the diagnosis of pneumonia [88]. Moreover, procalcitonin is not yet routinely available as a rapid POCT in primary care and its cost-effectiveness is still to be evaluated [25].

Previous evaluations with a similar scope [25,26,89] also found a significant reduction in antibiotic prescribing when using CRP-POCT. The most relevant to the scope of our review is a Cochrane review [25], which was based on six RCTs (3200 patients, 180 children)—also appraised in another review [89]—and showed an uncertain degree of antibiotic reduction, a non-significant increase in re-consultations, but a potential increase in hospital admissions. It was limited by the paucity of studies in children and studies using CRP guidance. The most recent review focused on acute infections in ambulatory care and based on 10 RCTs [26] showed that CRP guidance in addition to CRP-POCT reduced antibiotic prescribing and found no effect on re-consultations, hospital admissions, or other outcomes, and a relative lack of studies in children. Another review focused on LRTI and based on five RCTs, appraised in the Cochrane review [25], concluded that evidence did not support the use of CRP-POCT in primary care, but meta-analyses were not performed [90]. The reviews used 2 × 2 factorial group data from two cluster RCTs adding the effect of CST to both CRP-POCT and usual care.

Our review included 13 RCTs, all relevant to the study of CRP-POCT to reduce antibiotic prescribing for RTIs in primary care. It included all acute RTIs, appraised the impact of CRP-POCT on a wider range of patient-relevant and process outcomes and relevant subgroups not addressed in previous reviews, e.g., type of RTIs and clinical setting. Whenever possible, it used individual group rather than factorial groups to aggregate data in meta-analyses to minimise confounding of the effect of CRP-POCT versus usual care. It demonstrated a more robust effect of CRP-POCT in reducing antibiotic prescribing for RTIs, although with an increased rate of re-consultation, but no effect on hospital admissions. The effect on clinical recovery, ordering further investigations, and other relevant outcomes were similar to other reviews. In addition to the paucity of studies without the use of CRP guidance in older adults, our review also identified that the paucity of studies in children previously reported by other reviews has only slowly been addressed. Similarly, our review also identified that reporting of current and new patient-relevant and process outcomes is gradually increasing.

## 4. Literature Review Methods

This study is part of a series of systematic reviews designed to assess the evidence of interventions to improve antibiotic use in patients with RTIs in primary care. Its methods are described in detail elsewhere [91]. It follows the guidelines for the reporting of systematic reviews and meta-analyses (PRISMA) (see Supplementary Materials) [92]. The protocol is registered on PROSPERO [91] and followed the recommendations for systematic reviews of healthcare interventions [93,94].

### 4.1. Search Strategy

We searched for studies using 11 sources of literature by placing no restrictions on the language of publication, publication date, country of origin, or reported outcomes. We searched MEDLINE (EBSCOHost), EMBASE (Elsevier), The Cochrane Library (Wiley), CINHAL (EBSCOHost), PsychINFO (EBSCOHost), and Web of Science from their period of inception up to June 2017 (see supplementary materials). We also searched the Latin American and Caribbean Literature on Health Sciences (LILACS), Turning Research Into Practice database (TRIP), and the system for information on grey literature in Europe (http://opengrey.eu/) from all available dates until March–May 2019. Additionally, we screened the reference lists of included studies and relevant reviews published up to 2019. Experts and colleagues also provided us (up to May 2020) with potentially relevant studies.

### 4.2. Eligibility Criteria

We included studies investigating the use of CRP-POCT as an intervention to guide clinicians’ antibiotic prescribing decisions. Studies were eligible if they were randomised controlled trials (RCTs), cluster RCTs, or quasi-RCTs comparing the clinical effectiveness of CRP-POCT with usual care. RCTs had to be carried out in patients, both adults and children of all ages, presenting with common acute RTIs to primary care settings. We defined primary care as outpatient care services, including in-hours (e.g., paediatric and family practice clinics) and out-of-hours (e.g., walk-in clinics, emergency departments, hospital outpatients) services. We further limited study eligibility to RCTs reporting quantitative data for the outcomes of interest. We excluded RCTs in patients with COPD exacerbations and/or other pre-existing chronic pulmonary diseases, as well as RCTs from in-patient (e.g., hospitalised) settings.

### 4.3. Outcome Assessment

We defined the primary outcome as the effect of CRP-POCT on the rates of (any) antibiotics prescribed at the index consultation or any reported follow-up episode. We defined secondary outcomes as the effect of CRP-POCT on clinical recovery or resolution of symptoms, re-consultations, visit duration, RTIs during follow-up, ordering of additional or other diagnostic investigations, referral to hospital, hospital admission, mortality, patient enablement, and patient satisfaction.

### 4.4. Selection of Studies and Data Extraction

Four reviewers independently and in pairs performed the screening and selection of records retrieved from the searches. The first screening of titles and abstracts was performed for relevance and a second screening was performed to select potentially relevant publications for full-text evaluation. The first selection of full texts consisted of publications on POCT followed by a second selection of publications on CRP-POCT. Where necessary, we translated titles, abstracts, and full texts following the recommendations by the Centre for Research in Evidence-Based Practice [95]. We set out to prioritise the selection of studies in English, Spanish, and German, but we expanded to other languages where publications fit the inclusion criteria based on the translation of titles, abstracts, and/or full texts. Data extractions were performed using a priori developed forms by one reviewer and were independently verified by a second reviewer. Studies reported across more than one publication were treated as one unit. Differences in the judgment of study selection and data extractions were resolved by discussion, involving a third reviewer as arbitrator or both. The final list of included studies was confirmed with the team.

### 4.5. Assessment of Methodological Quality and Risk of Bias

Two reviewers independently assessed in duplicate the quality features of the included studies and differences were resolved by discussion. As detailed in the protocol [91], following established guidelines, we did not calculate a composite score [96], but we determined the internal validity (risk of bias) of studies by rating the adequacy of the studies’ core items based on the Cochrane risk of bias guidelines for RCTs [94]. We anticipated that due to the nature of the intervention, blinding patients and clinicians would not be possible, but blinded outcome assessment and reporting could help to identify whether studies are prone to selective reporting. We also assessed if all participants were followed up for the same length of time.

### 4.6. Statistical Analyses and Data Synthesis

We followed available guidelines to incorporate cluster-RCTs and to assess missing data [94]. We estimated the intervention effects using the unadjusted risk ratios (RRs) for binary data or the unadjusted weighted mean differences (MDs) for continuous data. We report the summary statistics and 95% confidence intervals (CIs) together with the exact *p*-values where data allowed their calculation. If at least three studies could be combined, a meta-analysis was performed in Cochrane RevMan (version 5.4.0) using the random-effects model due to clinical heterogeneity [97]. We quantified heterogeneity with the I^2^ statistic as low (≤25%), moderate (50%), substantial (up to 75%), and considerable (>75%) [98].

We limited subgroup, sensitivity, and meta-regression analyses to the primary outcome. We explored the effects of the interventions by pooling data into pre-specified subgroup analyses, including the type of randomisation (individual patient and cluster level), population groups (children and adults), RTI type, use of CRP (cut-off) guidance, and clinical setting. With sensitivity analyses, we assessed the effect of studies with dubious criteria for inclusion, unclear definitions of RTI, at-risk populations, special RCT design, different CRP analysers, or different continents. We performed a random effect meta-regression using the metafor package in R (version 3.6.1) to explore whether between-study heterogeneity could be explained by a set of modifiers. The model included publication year (centred with the mean of publication year = 2010), age groups (adults, adults and children, children), and whether CRP cut-off guidance was provided (no/yes). Where there were at least 10 trials per outcome, we assessed the potential for publication bias and small study effects by the inspection of funnel plots and by the Egger’s test for asymmetry of funnel plots [99].

### 4.7. Combined Benefit and Harm Effect of CRP-POCT

For the outcome of antibiotic prescribing and any outcome resulting in potential harm due to the intervention, we measured the combined benefit and harm effects of CRP-POCT (NNTnet) following recent recommendations [100]. The NNTnet metric is based on (1) the number needed to treat for benefit (NNTB), here the average number of patients needed to be seen by a clinician equipped to perform CRP-POCT to see the benefit (treatment without antibiotics) in one additional patient; and (2) the number needed to treat for harm (NNTH), here the average number of patients needed to be seen by a clinician equipped to perform CRP-POCT to see harm (any) in one additional patient.

## 5. Conclusions

So far, evidence of moderate to high quality shows that compared with usual care, using CRP-POCT to guide antibiotic prescribing for (lower and upper) RTIs in primary care can reduce antibiotic prescribing at index consultations especially if cut-off guidance is provided. This reduction in antibiotic prescribing appeared to increase the re-consultation rate but did not affect clinical recovery, resolution of symptoms, or hospital admissions. Limited evidence showed no significant effects of CRP-POCT on antibiotic prescribing at any point during 28 days of follow-up (*n* = 7) but less antibiotic prescribing at the index consultation in the GPs intervention sustained at 12 months (*n* = 1). The increased re-consultations and longer-term effects of CRP-POCT need further evaluation. The overall benefits of CRP-POCT (NNTnet = 11) on reducing antibiotic prescribing (NNTB = 8) outweigh the potential harms of increased re-consultations (NNTH = 27).

## Figures and Tables

**Table 1:** Characteristics of studies included in the review.

Study, Clinical Setting, Facilities and Location	Population	Interventionist and Training in the Intervention	Intervention and Number Randomised at Baseline (N)	Comparator and Number Randomised at Baseline (N)	CRP-POCT Turnaround Time and Manufacturer	CRP (cut-off) Guidance for Interpretation of CRP Levels
Schot, 2018 [61] The Netherlands Individual RCT 28 daytime general practices and 4 OOH services across three different regions in the Netherlands	Children with suspected LRTI presenting with acute cough of <21 days, reported a fever of >38 °C for <5 days Age, mean: 4 (SD 2.1), range: 3 months to 12 years Male, %: 51.5	GPs’ training in the intervention: n.r.	GP CRP + clinical assessment; N = 136	Usual Care: treatment decisions based on the clinical assessment as usual with no CRP; N = 165	≤4 min Afinion, Alere Technologies AS, Oslo, Norway	CRP < 10 mg/L = Pneumonia less likely, but should not be excluded if a child is ill, or when the duration of symptoms is <6 h CRP > 100 mg/L = Pneumonia much more likely; however, such levels can also be caused by viral infections CRP 10 to 100 mg/L = Likelihood of pneumonia increases with increasing CRP levels
Verbakel, 2016 [62,74,75,76] Belgium Cluster RCT 78 general practices across Flanders	Children with an acute infection lasting a maximum of 5 days at the initial contact Age, mean: 3.87 (SD 4.0), range: 1 month to 16 years Male, %: 52.7	GPs trained to perform the CRP test. Internal quality control performed according to the manufacturer’s instructions	GP CRP + clinical assessment; N = 1730 infectious episodes in 2773 patients	Usual Care: usual practice + CRP only if at clinical risk and presenting at least one symptom/sign of clinical concern ^1^; N = 1417 infectious episodes in 2773 patients	≤4 min Afinion AS100 Analyzer, Alere, USA	CRP < 5 mg/L = Low level = ruling out antibiotics CRP ≥ 5 mg/L = Elevated level
Van den Bruel, 2016 [63] United Kingdom (England) Individual RCT 2 OOH services in Oxfordshire	Children with an acute illness of ≤5 days, fever of ≥38 °C Age, mean: 2.8 (SD 2.8), range: 1 month to 16 years Male, %: 51.5	Physicians’ training in the intervention: n.r.	Physicians CRP + clinical examination according to usual care + clinical guidance on interpretation of CRP levels; N = 26	Usual Care: clinical examination according to usual care; N = 28	3–4 min Afinion, Alere Technologies	CRP < 20 mg/L = Serious infection is less likely CRP > 80 mg/L = Serious infection is more likely
Rebnord, 2016 [64,77] Norway Individual RCT 4 OOH services and 1 paediatric walk-in emergency hospital facility in Bergen	Children with fever or any respiratory symptoms Age, mean: 2.3 (SD 1.8), range: 0 to 6 years Male, %: 55.7	NPs trained in the study inclusion criteria and examination procedures, performed a clinical examination and CRP tests for all children before consultation with the doctor	NP CRP pre-tested + NP clinical examination before consultation with doctors + consultation with paediatricians or physicians with an assessment of CRP results; other tests were also available; N = 138	Usual Care: NP clinical examination with no CRP assistance + clinical assessment by paediatricians or physicians + CRP if necessary, on individual indication; other tests were also available; N = 259	≤2 min QuikRead Go, Orion Diagnostica	n.r.
Do, 2016 [65] Vietnam Individual RCT 10 primary health-care centres - northern Vietnam (routine, urgent care and hospital referral) within a 60 km radius of Hanoi. Rural sites: outpatient clinics - district general hospital (Ba Vi hospital) 60 km West Hanoi	Children and adults with suspected non-severe acute RTI, with at least one focal and one systemic sign or symptom lasting for less than 2 weeks Age, mean: 21.2 (SD 23.8), range: 1 to 65 years Male, %: 39.9	Physicians trained to use specific CRP cut-offs with initial workshop and further training during onsite implementation. Training materials: oral presentations and written information leaflets for doctors and health centres; posters and desk reminders with recommended cut-off values for specific age groups	Physician CRP + guidance based on CRP cut-off values adapted for use in children + GPs advised to use their clinical discretion for CRP values between thresholds, and could potentially perform further examinations at their clinical discretion; all patients received a routine medical history examination; N = 1017	Usual Care: routine practice + use of local treatment guidelines + potential to perform further examinations at the discretion of the treating physician; all patients received a routine medical history examination; N = 1019	≤3 min CRP single test kit NycoCard II Reader, Alere Technologies, Norway	General CRP ≤ 20 mg/L = No antibiotics for patients aged 6–65 years Children CRP ≤ 10 mg/L = No antibiotics for patients aged 1–5 years CRP > 20 to <50 mg/L = No specific recommendation but clinicians were advised to use their clinical discretion CRP ≥ 50 mg/L = Should generally receive antibiotics and hospital referral should be considered Adults CRP > 20 to <99 mg/L = No specific recommendation but clinicians were advised to use their clinical discretion CRP ≥ 100 mg/L = Should generally receive antibiotics and hospital referral should be considered
Andreeva, 2014 [66] Russia Cluster RCT 18 general practices: 9 Arkhangelsk region, 9 Murmansk region	Adults with acute cough/LRTI (acute bronchitis, pneumonia, infectious exacerbations of COPD or asthma), illness of fewer than 28 days duration Age, mean: 50.8 (SD n.r.), range: ≥18 years Male, %: 27.4	GPs: two vocational training sessions on CRP test, theoretical and practical information, guidelines on the interpretation of CRP, a summary of the literature on RTI and CRP role, and paper cases of patients with different RTIs and different CRP values were discussed	GP CRP + guidance on the interpretation of CRP results +/- accessibility and order of chest radiography (for all patients) and other investigations (e.g., a culture of sputum, spirometry, electrocardiogram) when necessary; N = 8 GP offices, 101 patients	Usual Care: clinical assessment with no CRP +/- chest radiography for all patients and other investigations when necessary; choice of antibiotic therapy regimen left at the discretion of physicians; N = 9 GP offices, 78 patients	≤5 min Afinion test system, Axis-Shield, Norway	CRP < 20 mg/L = Antibiotics usually not needed CRP > 50 mg/L = Antibiotics could be indicated considering duration of illness
Little, 2013 [67,78,79] Spain, England, Wales, Poland, Belgium, The Netherlands Individual RCT 111 GP practices from GP networks of at least 2 general practices in the localities of study centres of all 6 countries	Adults with an acute cough lasting up to 28 days, or acute LRTI as the main diagnosis (despite cough not being the most prominent symptom) and acute URTI (sore throat, otitis media, sinusitis, influenza, and coryzal illness) Age, mean: 26.4 (SD 15.0), range: ≥18 years Male, %: 36.7	GPs: a run-in period of several weeks before data collection to practise using the device, internet training on how to target testing and how to negotiate with the patient about management decisions	GP CRP testing + guidance on the interpretation of CRP testing and prescribing + internet training on how to target testing and how to negotiate with the patient about management decisions ^2^; N = 1062	1) Usual Care: GPs assessed and managed patients according to the practice’s normal procedures; N = 870	≤5 min QuikRead CRP kits, Orion Diagnostica, Espoo, Finland	CRP ≤ 20 mg/L = Withhold antibiotics: self-limiting LRTI CRP 21 to 50 mg/L = Withhold antibiotics for most cases: most patients have self-limiting LRTI; assess signs, symptoms, risk factors; CRP is important CRP 51 to 99 mg/L = Withhold antibiotics in the majority of cases and consider Delayed antibiotics in the minority of cases: assessment of signs, symptoms, risk factors; CRP is crucial CRP ≥ 100 mg/L = Prescribe antibiotics: severe infection
Gonzales, 2011 [68] United States Individual RCT 1 ED supporting a 3-year emergency medicine residency program located in a large, Midwestern metropolitan city in the United States	Adults with a new cough lasting ≤21 days, at least one other symptom of acute RTI (fever, sore throat, night sweats, body aches, nasal or chest congestion, shortness of breath) Age, mean: 41.2 (SD 12.5), range: ≥18 years Male, %: 32.1	NPs performed CRP testing; management algorithms placed for doctors in the medical chart. ED visits and house staff received current evidence on CRP levels as adjuncts in the diagnosis of pneumonia (or other antibiotic-responsive illnesses), a 1.5-h educational seminar on evidence-based recommendations for evaluation and treatment of acute cough illness and community-acquired pneumonia for adults	NP CRP + clinical algorithm to guide physicians on the ordering of chest x-ray and on antibiotic treatment for adults with acute cough illness and community-acquired pneumonia + activation of GPs in using the algorithm with a statement (“Please consider using this algorithm in your clinical care decisions, although it should not substitute for your clinical judgment”); N = 69	Usual Care: no CRP testing + clinical management algorithm to guide recommendations for a chest x-ray and antibiotic treatment for adults with acute cough illness (based on a clinical algorithm for predicting pneumonia) + activation of GPs in using the algorithm with a statement (“Please consider using this algorithm in your clinical care decisions, although it should not substitute for your clinical judgment”); N = 62	1 min QuikRead CRP, Orion Corporation, Orion Diagnostica, Espoo, Finland	Low to Intermediate (<30%) probability of Pneumonia = abnormal signs OR abnormal chest examination: CRP < 10 mg/L = Normal = No antibiotics and no chest x-ray CRP 10 to 99 mg/L = Intermediate: Not helpful CRP ≥ 100 mg/L = High: Perform chest x-ray: − normal x-ray = no antibiotics − abnormal x-ray = antibiotics High (>30%) probability of Pneumonia = abnormal signs WITH abnormal chest examination: CRP < 100 mg/L = Perform chest x-ray: − normal x-ray = no antibiotics − abnormal x-ray = antibiotics CRP ≥ 100 mg/L = Perform chest x-ray: − consider antibiotics regardless of chest x-ray results
Cals, 2010 [69] The Netherlands Individual RCT 11 family practice centres in the south-eastern Netherlands	Adults with a current episode of LRTI (cough lasting <4 weeks with ≥1 of 4 focal signs and symptoms and at least one systemic sign and symptom) or Rhinosinusitis (episodes lasting <4 weeks with at least one symptom of rhinorrhoea history and blocked nose; and at least one other symptom or sign) Age, mean: 44.3 (SD 13.8), range: ≥18 years Male, %: 30.7	NPs received CRP device demonstration, did not communicate test result to GP or patient until after the study. GPs were informed about the trial procedure, received a 30-min practice-based seminar on the EB use of CRP, stressing the additional CRP value to rule out serious infection with emphasis on using CRP together with clinical findings, a 4-week run-in period before recruitment to get familiar with CRP devices and interpretation	NP CRP +/- GP clinical assessment and management of antibiotic therapy based on CRP results + decision-making on a management strategy including immediate, delayed or no antibiotics; N = 129	Usual Care: no CRP testing + antibiotic therapy based on clinical assessment + decision-making on a management strategy including immediate, delayed or no antibiotics; N = 129	≤3 min QuikRead CRP analysers, Orion Diagnostica, Espoo, Finland	CRP < 20 mg/L = No antibiotics CRP > 100 mg/L = Immediate antibiotics CRP 20 to 99 mg/L = Delayed prescription at physicians’ discretion
Cals, 2009 [70,80,81] The Netherlands Cluster RCT 20 general practices from the South-Eastern part of Noord-Brabant province in the Netherlands including urban and rural areas; GP practices are geographically spread throughout this region	Adults with suspected LRTI, with a cough lasting <4 weeks and with one focal and one systemic symptom Age, mean: 45.4 (SD 8.2), range: ≥18 years Male, %: 38.6	GPs received a 30-min practice-based guideline on how to use CRP, ruling out a serious infection. Practice nurses received an introduction to technical and practical aspects. Practices received an 8-week-run-in period before recruitment to enable familiarisation with CRP devices and interpretation	GP CRP + guidance on the interpretation of results based on CRP cut-off values with an emphasis on the additional value of CRP in ruling out serious infection + familiarisation with CRP devices and interpretation of results; N = 110	(1) Usual Care: Dutch guidelines-informed clinical assessment for the diagnosis and management of acute cough, and therapeutic advice for LRTI; practices were informed that they would receive a CRP device and/or communication training after the study period; N = 120 (2) ECST: motivational interviewing built around 11 key tasks ^3^; practices were informed that they would receive a CRP device and/or the communication training after the study period; N = 84 (3) CRP + ECST; N = 117	≤3 min NycoCard II Reader, Axis-Shield, Norway	CRP < 20 mg/L = Withhold antibiotics in most patients with low values (<75% of patients with LRTI in primary care): pneumonia extremely unlikely CRP 21 to 99 mg/L = Delayed antibiotics at discretion of physicians: patients should be carefully assessed based on the combination of medical history, physical examination, and CRP value: - CRP 20 to 50 mg/L = pneumonia very unlikely - CRP 50 to 100 = clear infection, most likely acute bronchitis, possibly pneumonia: combine with clinical findings; CRP is very important CRP > 100 = Immediate antibiotics: severe infection, Pneumonia more likely
Takemura, 2005 [71] Japan Individual RCT 1 general/internal medicine clinic of Nishi-Ohmiya regional/community hospital	Children and adults with a clinically relevant fever of >37.5 °C, and symptoms suspected of infection at the time of or during the week before an initial consultation Age, mean: 34.9 (SD 15.4), range: 8 to 83 Male, %: 55.9	Physicians’ training in the intervention: n.r.	Advanced testing group: Physician CRP + WBC testing before initial consultation + information on CRP and WBC normal reference levels + if considered necessary, potential to perform urgent testing after history taking and physical examination + results of non-urgent additional or subsequent tests evaluated on patient’s next visit; N = 147	Usual Care: non-advanced testing group defined as standard management and treatment with no CRP before initial consultation + decision-making on antibiotic management and treatment based on history taking and physical examination + if considered necessary, potential to perform urgent testing after history taking and physical examination; N = 154	CRP approx. 40–50 min; WBC 10 min CRP multichannel analyser, model TBA-30FR; Toshiba, Saitama City, Japan	CRP ≤ 5 mg/L = Normal reference intervals
Diederichsen, 2000 [72,82] Denmark Individual RCT 35 General practices in the County of Funen in Denmark	Children and adults with respiratory infections Age, mean: 41 (SD 14.2), range: median 37 (range: 0 to 90) Male, %: 42.8	GPs discussed, before the start of the study, the trial procedure with the project leader and a product specialist from Nycomed; a supervised test trial was carried out	GP CRP + clinical assessment to guide antibiotics prescribing + information on the normal levels of CRP for antibiotic prescribing but no strict guidelines were given; N = 414	Usual Care: clinical assessment only; N = 398	≤3 min NycoCard CRP Reader, Nycomed, Alere Technologies, Afinion, Norway	CRP < 10 mg/L = Normal CRP < 10 mg/L = Seldom the result of bacterial infection CRP < 50 mg/L = Seldom the result of bacterial infection
Melbye, 1995 [73] Norway Individual RCT 10 General practices in Northern Norway	Adults with signs of pneumonia, bronchitis and asthma, who presented with symptoms of coughing or heavy breathing, or who had chest pain that was aggravated by coughing or deep inspiration Age, mean: 49.25 (SD 11.6), range: ≥18 years Male, %: 36.9	GPs’ training in the intervention: n.r.	GP CRP + doctors’ preliminary decision on antibiotic treatment + guide on antibiotic prescribing based on the duration of illness following recommended CRP cut-off values (if a preliminary decision needed to change in light of the CRP results); N = 108	Usual Care, N = 131	≤3 min NycoCard CRP Reader, Nycomed, Alere Technologies, Afinion, Norway	Disease duration 0–24 h CRP < 50 mg/L = No change in clinical decision CRP ≥ 50 mg/L = Antibiotics Disease duration 1–6 days CRP < 11 mg/L = No antibiotic prescribing CRP 11–49 mg/L = No change in clinical decision CRP ≥ 50 mg/L = Antibiotics Disease duration ≥7 days CRP < 11 mg/L = No antibiotic prescribing CRP 11–24 mg/L = No change in clinical decision CRP ≥ 25 mg/L = Antibiotics

Note. OOH, Out-Of-Hours care services; ED, Emergency Department services; SD, standard deviation; CRP, C-Reactive Protein; ECST, Enhanced Communication Skills Training; GP, General Practice or General Practitioner; NP, nurse or Nurse Practitioner; RTI, Respiratory Tract Infection; URTI, Upper Respiratory Tract Infection; LRTI, Lower Respiratory Tract Infection; COPD, Chronic Obstructive Pulmonary Disease; EB, Evidence-Based; n.r., not reported. ^1^ Clinical risk was assessed with a validated clinical decision rule during clinical assessment; in a clinical risk group, CRP was dependent on the presence of at least one of the following clinical features: breathlessness, a body temperature of at least 40 °C, diarrhoea in children 12–30 months of age, and clinician concern. ^2^ Training in how to target testing in cases of clinical uncertainty consisted of e.g., patients with abnormal auscultation, fever dyspnoea. ^3^ Motivational interviewing was built around 11 key aspects including, for example, exploring patients’ fears and expectations, asking patients’ opinion on AB, and outlining the natural duration of cough in LRTI.

**Table 2:** Methodological features and risk of bias in the included studies.

Study (First Author, Publication Year)	Inclusion Criteria	Exclusion Criteria	Primary Outcome(s)	Secondary Outcome(s)	Sample Size and Power	Attrition ≤20% 1ry Outcome (Attrition Bias)	Random Sequence Generation (Selection Bias)	Allocation Concealment (Selection Bias)	Blinding of Clinicians (Performance Bias)	Blinding of Patients (Performance Bias)	Blinding of Outcome Assessors 1ry Outcome (Detection Bias)	Blinding - Outcome Assessors 2ry Outcome (dEtection Bias)	Incomplete Outcome Data 1ry Outcome (Attrition Bias)	Selective Reporting (Reporting Bias)	Participants Comparable at Baseline	Same Length of Follow-Up	Source of Funding
Schot, 2018 [61] The Netherlands Individual RCT	✓	✓	✓	✓	✓	✓	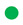	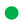	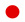	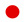	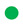	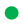	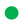	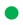	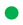	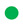	Netherlands Organization for HRD; Alere Technologies AS; SALTRO & Star Medical Diagnostic Centre
Verbakel, 2016 [62,74,75,76] Belgium Cluster RCT	✓	✓	✓	✓	✓	✓	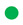	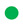	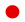	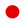	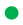	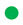	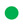	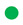	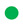	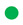	NIHDI, Research Foundation Flanders, NIHR Diagnostic Evidence Co-operative Oxford
Van den Bruel, 2016 [63] United Kingdom (England) Individual RCT	✓	✓	✓	✓	✓	✓	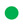	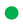	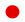	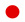	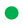	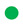	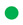	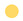	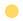	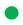	National School for Primary Care Research
Rebnord, 2016 [64,77] Norway Individual RCT	✓		✓	✓	✓	✓	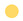	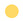	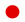	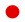	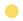	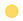	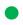	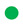	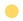	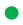	Norwegian Research Fund
Do, 2016 [65] Vietnam Individual RCT	✓	✓	✓	✓	✓	✓	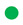	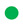	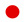	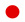	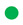	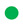	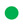	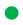	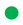	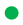	Welcome Trust UK and Global Antibiotic Resistance Partnership; Alere Technologies
Andreeva, 2014 [66] Russia Cluster RCT	✓	✓	✓	✓	✓	✓	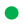	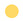	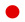	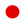	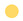	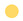	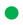	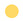	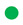	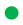	Not reported
Little, 2013 [67,78,79] Spain, England, Wales, Poland, Belgium, The Netherlands Cluster RCT	✓	✓	✓	✓	✓	✓	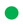	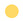	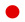	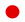	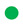	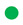	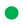	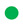	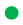	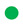	European Commission Framework Programme, NIHR, Research Foundation Flanders
Gonzales, 2011 [68] United States Individual RCT	✓	✓	✓	✓	✓	✓	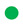	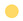	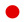	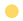	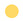	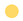	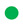	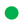	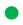	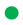	TRIP initiative and agency for HRQ, HSRD Service of the Department of Veterans Affairs
Cals, 2010 [69] The Netherlands Individual RCT	✓	✓	✓	✓	✓	✓	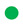	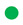	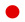	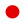	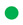	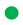	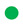	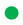	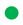	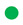	Orion Diagnostica Espoo Finland
Cals, 2009 [70,80,81] The Netherlands Cluster RCT	✓	✓	✓	✓	✓	✓	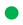	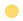	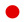	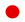	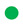	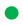	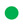	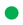	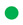	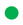	Netherlands Organisation for HRD, Wales Office for R&D, NIHSC funded the South East Wales Trial Unit
Takemura, 2005 [71] Japan Individual RCT	✓					✓	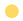	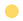	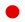	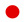	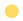	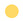	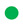	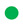	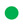	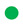	International Clinical Pathology Centre Tokyo
Diederichsen, 2000 [72,82] Denmark Individual RCT	✓	✓			✓	✓	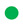	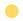	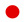	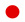	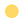	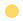	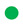	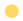	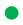	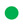	Not reported
Melbye, 1995 [73] Norway Individual RCT	✓	✓	✓		✓	✓	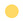	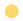	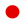	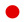	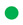	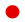	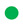	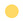	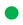	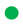	Norwegian Research Academy and Nycomed Pharma

Note. Green = adequate; Yellow = unclear; Red = not adequate. NIHDI, National Institute for Health and Disability Insurance; NIHR, National Institute for Health and Research; TRIP, Translating Research into Practice initiative; HRQ, Healthcare Research and Quality; HSRD, Health Services Research and Development; R&D, Research and Development; NIHSC, National Institute for Health and Social Care.

## Supplementary Materials

Additional supporting material is contained in the supplementary materials and it is available online at https://www.mdpi.com/2079-6382/9/9/610/s1.
