# Supplementary material for: Point-of-Care C-Reactive Protein Testing to Reduce Antibiotic Prescribing for Respiratory Tract Infections in Primary Care: Systematic Review and Meta-Analysis of Randomised Controlled Trials
Source: Antibiotics (Basel). 2020 Sep 16;9(9):610. doi: 10.3390/antibiotics9090610 (PMC7559694; doi:10.3390/antibiotics9090610)
Supplement: Supplementary file 1 [file antibiotics-09-00610-s001.pdf]

Point-of-care C - reactive protein as a stewardship intervention to reduce antibiotic prescribing in patients with respiratory tract infections in primary care: a systematic review and meta-analysis of randomised controlled trials

APPENDIX: TABLES AND FIGURES

Appendix, Table of Contents

Systematic Review Checklist 3

Appendix, Table 1. PRISMA checklist. 3

Search strategy 6

Appendix, Table 2. a) Search strategy in *EMBASE (Elsevier). 6

Appendix, Table 2. b) Search strategy in *LILACS. 8

List of excluded studies 11

Figures: risk of bias 14

Appendix, Figure 1. Graph for the risk of bias assessment across all included RCTs. 14

Figures: primary outcomes 15

Appendix, Figure 2. Antibiotic prescribing at index consultations by type of RTIs. 15

Appendix, Figure 3. a) Antibiotic prescribing at index consultations: CRP (cut-off) guidance applied versus not applied to withhold antibiotics. 16

Appendix, Figure 3. b) Antibiotic prescribing at index consultations: CRP (cut-off) guidance applied by age groups versus not applied to withhold antibiotics. 17

Appendix, Figure 4. Antibiotic prescribing at index consultations by Healthcare Settings. 18

Appendix, Figure 5. a) Antibiotic prescribing at any point during 28 days follow-up. 19

Appendix, Figure 5. b) Antibiotic prescribing at any point during 28 days follow-up by RTI type. 19

Appendix, Figure 5. c) CRP-POCT compared with Usual Care. Antibiotic prescribing within 3 months........ 20

Appendix, Figure 5. d) CRP-POCT compared with Usual Care. Antibiotic prescribing at 12 months follow-up........ 20

Appendix, Figure 5. e) CRP-POCT compared with Usual Care. Antibiotic prescribing at 12 months follow-up by RTI type. 20

Appendix, Figure 5. f) CRP-POCT compared with Usual Care. Antibiotic prescribing at follow-up: within 3.5 years. 21

Figures: secondary outcomes 22

Appendix, Figure 6. a) Clinical recovery within 7 days. 22

Appendix, Figure 6. b) Clinical recovery within 28 days. 22

Appendix, Figure 7. Time in mean number of days to resolution of symptoms. 22

Appendix, Figure 8. Re-consultations within 30 days. 23

Appendix, Figure 9. Intention to re-consult. 23

Appendix, Figure 10. Referrals to Hospital. 24

Appendix, Figure 11. Admissions to Hospital. 24

Appendix, Figure 12. a) Ordering of Investigations: additional tests. 25

Appendix, Figure 12. b) Ordering of Investigations: chest x-rays. 25

Appendix, Figure 13. Patient Satisfaction. 26

Appendix, Figure 14. Patient Enablement. 26

Individual trial estimates 27

Appendix, Table 4. Individual trial estimates from data not combined in meta-analyses.. 27

Sensitivity analyses 28

Appendix, Table 5. Sensitivity analyses. 28

Funnel Plot and Egger's Test 29

Appendix, Figure 15. Funnel plot and Egger Test to assess publication bias. 29

Meta-regression 30

Appendix, Table 6. Meta-regression results for the outcome of overall antibiotic prescribing at index consultations. 30

Appendix, Figure 16. Funnel plots for meta-analysis and meta-regression considering studies split according to age. 32

Systematic Review Checklist

Appendix, Table 1. PRISMA checklist.

Section/topic	#	Checklist item	Reported on page #
**TITLE**			
Title	1	Identify the report as a systematic review, meta-analysis, or both.	1
**ABSTRACT**			
Structured summary	2	Provide a structured summary including, as applicable: background; objectives; data sources; study eligibility criteria, participants, and interventions; study appraisal and synthesis methods; results; limitations; conclusions and implications of key findings; systematic review registration number.	1
**INTRODUCTION**			
Rationale	3	Describe the rationale for the review in the context of what is already known.	2
Objectives	4	Provide an explicit statement of questions being addressed with reference to participants, interventions, comparisons, outcomes, and study design (PICOS).	2
**METHODS**			
Protocol and registration	5	Indicate if a review protocol exists, if and where it can be accessed (e.g., Web address), and, if available, provide registration information including registration number.	9
Eligibility criteria	6	Specify study characteristics (e.g., PICOS, length of follow-up) and report characteristics (e.g., years considered, language, publication status) used as criteria for eligibility, giving rationale.	9
Information sources	7	Describe all information sources (e.g., databases with dates of coverage, contact with study authors to identify additional studies) in the search and date last searched.	9
Search	8	Present full electronic search strategy for at least one database, including any limits used, such that it could be repeated.	9
Study selection	9	State the process for selecting studies (i.e., screening, eligibility, included in systematic review, and, if applicable, included in the meta-analysis).	9

Data collection process	10	Describe method of data extraction from reports (e.g., piloted forms, independently, in duplicate) and any processes for obtaining and confirming data from investigators.	10
Data items	11	List and define all variables for which data were sought (e.g., PICOS, funding sources) and any assumptions and simplifications made.	10
Risk of bias in individual studies	12	Describe methods used for assessing risk of bias of individual studies (including specification of whether this was done at the study or outcome level), and how this information is to be used in any data synthesis.	10
Summary measures	13	State the principal summary measures (e.g., risk ratio, difference in means).	10
Synthesis of results	14	Describe the methods of handling data and combining results of studies, if done, including measures of consistency (e.g., I^2) for each meta-analysis.	10
Risk of bias across studies	15	Specify any assessment of risk of bias that may affect the cumulative evidence (e.g., publication bias, selective reporting within studies).	10
Additional analyses	16	Describe methods of additional analyses (e.g., sensitivity or subgroup analyses, meta-regression), if done, indicating which were pre-specified.	10, 11
**RESULTS**			
Study selection	17	Give numbers of studies screened, assessed for eligibility, and included in the review, with reasons for exclusions at each stage, ideally with a flow diagram.	2, Figure 1
Study characteristics	18	For each study, present characteristics for which data were extracted (e.g., study size, PICOS, follow-up period) and provide the citations.	2, Table 1
Risk of bias within studies	19	Present data on risk of bias of each study and, if available, any outcome level assessment (see item 12).	3
Results of individual studies	20	For all outcomes considered (benefits or harms), present, for each study: (a) simple summary data for each intervention group (b) effect estimates and confidence intervals, ideally with a forest plot.	3-6
Synthesis of results	21	Present results of each meta-analysis done, including confidence intervals and measures of consistency.	4-6, Figures 2-5, Supplementary materials
Risk of bias across studies	22	Present results of any assessment of risk of bias across studies (see Item 15).	Table 2, Supplementary appendix

Additional analysis	23	Give results of additional analyses, if done (e.g., sensitivity or subgroup analyses, meta-regression [see Item 16]).	Supplementary materials
**DISCUSSION**			
Summary of evidence	24	Summarize the main findings including the strength of evidence for each main outcome; consider their relevance to key groups (e.g., healthcare providers, users, and policy makers).	6
Limitations	25	Discuss limitations at study and outcome level (e.g., risk of bias), and at review-level (e.g., incomplete retrieval of identified research, reporting bias).	7
Conclusions	26	Provide a general interpretation of the results in the context of other evidence, and implications for future research.	11
**FUNDING**			
Funding	27	Describe sources of funding for the systematic review and other support (e.g., supply of data); role of funders for the systematic review.	11

From: Moher D, Liberati A, Tetzlaff J, Altman DG, The PRISMA Group (2009). Preferred Reporting Items for Systematic Reviews and Meta-Analyses: The PRISMA Statement. PLoS Med 6(7): e1000097. doi:10.1371/journal.pmed1000097

For more information, visit: **www.prisma-statement.org**

Search strategy

Appendix, Table 2. a) Search strategy in *EMBASE (Elsevier).

***EMBASE (Elsevier) with conference abstracts.**

**Concept 1 - RTIs**
'acute sinusitis'/exp OR 'acute otitis media'/exp OR 'common cold'/exp OR 'sore throat'/exp OR 'pharyngitis'/exp OR 'tonsillitis'/exp OR 'rhinitis'/exp OR 'rhinopharyngitis'/exp OR 'laryngitis'/exp OR ('bronchitis'/exp NOT 'chronic bronchitis'/exp) OR 'respiratory tract disease'/de OR 'pleurisy'/exp OR 'pneumonia'/exp OR 'coughing'/exp OR 'sneezing'/exp OR 'otalgia'/exp OR 'influenza'/exp OR (flu OR influenza OR "Respiratory Syncytial Vir*" OR rsv OR rti OR urti OR rhinit* OR pharyngit* OR nasopharyngit* or rhinopharyngit* OR rhinorrhoea or rhinorrhea OR tonsillit* OR laryngit* OR mononucleo* OR croup OR pseudocroup or tracheobronchit* or laryngotracheobronchit* OR pneumon* or pleuropneumon* or bronchopneumon* OR pleurisy OR cough* or sneez* OR earache* OR (common NEAR/3 (cold OR colds)) OR ((runny or running or discharg* or congest* or blocked or stuff* or dripping) NEAR/3 (nose* or nasal)) OR ((respiratory OR chest) NEAR/3 (infect* OR inflam*)) OR (throat* NEAR/3 (inflam* or infect*)) OR (acute NEAR/3 (sinusit* OR bronchit* OR bronchiolit* OR "otitis media" OR nasosinusit* or rhinosinusit*)) OR (acute NEAR/3 "middle ear*" NEAR/3 infect*)):ab,ti
**Concept 2 - Antibiotics**
'antibiotic agent'/exp OR 'quinoline derived antiinfective agent'/exp OR 'sulfonamide'/exp OR 'amantadine'/exp OR 'amantadine sulfate'/exp OR 'arbidol'/exp OR 'rimantadine'/exp OR 'umifenovir'/exp OR 'zanamivir'/exp OR 'oseltamivir'/exp OR 'peramivir'/exp OR (Antibacterial* or Anti-bacterial* or Antibiotic* or Anti-biotic* or anti-infective OR Macrolide* or beta-Lactam* or Antimicrobial* or Anti-microbial* or Penicillin or Methicillin or ampicillin or azithromycin or Cephalexin OR fluoroquinolon* OR quinolon* OR quinolin* OR sulfonamid* OR sulphonamid* OR sulfamoyl):ti,ab OR (amantadine OR adamantanamine OR endantadine OR enzil OR amantix OR amantrel OR amazolon OR aminoadamantane OR aminoadamantine OR atarin OR boidan OR hofcomant OR infectoflu OR mantadan OR mantadix OR mantidan OR midantane OR nurelin OR padiken OR paritrel OR parkintrel OR pk-merz OR prayanol OR protexin OR symadine OR symetrel OR symmetrel OR tregor OR viregyt OR virofral OR virosol OR virucid OR 'amantadine sulfate' OR 'amantadine sulphate' OR grippin OR 'arbidol' OR 'rimantadin*' OR flumadine OR flumandine OR gabirol OR germic OR remantadin OR remantadine OR ro22-1859 OR roflual OR 'umifenovir' OR 'zanamivir' OR gg167 OR gr-121167 OR relenza OR 'oseltamivir' OR tamiflu OR 'peramivir' OR bcx-1812 OR bcx1812 OR peramiflu OR rapiacta OR rapivab OR rwj-270201 OR rwj270201):ti,ab
**Concept 3 - Setting**
'ambulatory care'/exp OR 'outpatient department'/exp OR 'general practice'/exp OR 'general practitioner'/exp OR 'primary health care'/exp OR 'outpatient'/exp OR 'outpatient care'/exp OR 'home visit'/exp OR 'emergency health service'/exp OR 'pharmacist'/exp OR 'pharmacy technician'/exp OR 'pharmacy'/exp OR (ambulatory NEAR/3 (care or setting? or facilit* or ward? or department? or service?)):ti,ab or (practi* or physician* or doctor* or Clinician* OR pharmacist* OR pharmacy OR pharmacies):ti,ab or ("primary care" or "primary health care" or "primary healthcare"):ti,ab or ("after hour*" or afterhour* or "out of hour*" or ooh):ti,ab or ((clinic OR clinics OR office) NEAR/3 (visit OR visits)):ti,ab or ((health* or medical) NEAR/2 (center OR centers or centre*)):ti,ab or outpatient*:ti,ab or (emergency NEAR/3 (care or setting* or facility OR facilities or ward OR wards or department* or service*)):ti,ab
**Concept 4.a - Interventions1**
'rapid test'/exp OR 'chemiluminescence immunoassay'/exp OR 'immunofluorescence test'/exp OR 'inhalation test'/exp OR 'laboratory test'/exp OR 'Gram staining'/exp OR 'calcitonin derivative'/exp OR 'blood cell count'/exp OR 'blood gas'/exp OR 'enzyme linked immunosorbent assay'/exp OR 'nucleic acid amplification'/exp OR 'latex agglutination test'/exp OR 'thorax radiography'/exp OR 'C reactive protein'/exp OR 'polymerase chain reaction'/exp OR 'colorimetry'/exp OR 'cell culture'/exp OR (("point of care" OR poc) NEAR/3 (diagnos* or test* or assay* or kit or kits)):ti,ab OR ((immediat* OR routine) NEAR/3 (test* or diagnos*)):ti,ab OR ((rapid* or quick* or swift* or office*) NEAR/3 (test* or kit or kits or assay* or swab*)):ti,ab

OR (strep* NEAR/5 (test* or kit or kits or assay* or swab*)):ti,ab OR procalcitonin:ti,ab OR "c-reactive protein*":ti,ab OR monospot*:ti,ab OR ((antibod* OR gram) NEAR/3 stain*):ti,ab OR (Fluoresc* NEAR/3 Antibod*):ti,ab OR ("reverse transcriptas*" NEAR/3 ("polymerase chain reaction*" or pcr)):ti,ab OR ((singleplex* or multiplex*) NEAR/3 ("polymerase chain reaction*" or pcr)):ti,ab OR ((chest* or thorac* OR thorax) NEAR/3 (radiogra* or x-ray*)):ti,ab OR ((leukocyt* or "white blood cell*" or wbc OR neutrophil) NEAR/3 (test* or count*)):ti,ab OR (blood NEAR/2 (gas or gases) NEAR/3 (analy* or test*)):ti,ab OR elisa:ti,ab OR immunoassay:ti,ab OR ((agglutinin OR coagglutinin OR breath-based OR inflammatory) NEAR/3 test*):ti,ab OR (cell NEAR/3 (culture* OR colon*)):ti,ab
**Concept 4.b - Interventions2**
'decision support system'/exp OR 'information system'/exp OR 'health personnel attitude'/exp OR 'patient information'/exp OR 'patient education'/exp OR 'health promotion'/exp OR 'practice guideline'/exp OR 'protocol compliance'/exp OR 'inappropriate prescribing'/exp OR 'drug misuse'/exp OR 'drug utilization'/exp OR 'absenteeism'/exp OR 'return to work'/exp OR Intervention*:ti OR audit*:ti OR feedback:ti OR ((clinical OR clinician* OR performance OR outcome OR regulatory) NEAR/3 (intervention* OR audit* OR feedback OR review)):ti,ab OR (decision* NEAR/3 (make OR makes OR making OR made OR tool* OR system* OR method* OR approach*)):ti,ab OR ((drug* OR medical* OR pharmac*) NEAR/3 (utiliz* OR misuse OR misusage OR limit* OR restriction* OR banning)):ti,ab OR ((prescib* OR dispens* OR utiliz*) NEAR/3 (formular* OR restrict* OR control* OR banning)):ti,ab OR (attitud* NEAR/3 ('health personnel' OR doctor OR physician OR practitioner*)):ti,ab OR (practice* NEAR/3 pattern*):ti,ab OR (risk* NEAR/3 assess*):ti,ab OR ((education OR teach* OR information OR instruct*) NEAR/3 (professional OR "interprofessional" OR material* OR method* OR campaign* OR strateg* OR patient* OR public OR program*)):ti,ab OR (communication NEAR/3 (skill* OR train* OR improve* OR strateg*)):ti,ab OR ((public OR health OR awareness OR local OR national OR regional OR wise*) NEAR/3 (campaign* OR strategy OR strategies)):ab OR campaign:ti OR strategy:ti OR strategies:ti OR ((professional* OR doctor* OR physician* OR practitioner*) NEAR/3 patient* NEAR/3 (relation* OR interaction OR request* OR ask*)):ti,ab OR ((guideline* OR protocol* OR workflow* OR recommendation* OR path*)NEAR/3 (adheren* OR comply* OR complian* OR obey*)):ti,ab OR ((professional* OR clinical*) NEAR/3 (competen* OR skill* OR abilit* OR knowledg*)):ti,ab OR ((inappropriat* OR imprudent* OR unreasonab* OR unwis* OR improper* OR unnecessar* OR useless* OR incorrect* OR worthless* OR useless* OR unneeded OR gratuitous* OR ineffect* OR overus* OR 'over us*') NEAR/3 (prescri* OR give OR gives OR giving OR issue OR issuing OR provid* OR use OR usage OR utiliz*)):ti,ab OR ((appropriat* OR judicious* OR judge* OR judging OR wise* OR prudent* OR sensible OR reasonabl* OR proper* OR necessar* OR useful* OR correct* OR worthwhile* OR needed OR effectiv* OR delay* OR postpon*) NEAR/3 (prescri* OR give OR gives OR giving OR issue OR issuing OR provid* OR use OR usage OR utiliz*)):ti,ab OR ((non-antibiotic OR nonantibiotic) NEAR/3 (prescribe* OR us*)):ti,ab OR ((critical* OR clinical*) NEAR/3 (path OR paths OR pathway* OR algorithm* OR "prediction rule*")):ti,ab OR ((antibiotic* OR 'anti biotic*' OR 'anti microb*' OR antimicrob*) NEAR/3 steward*):ti,ab OR ((system* OR computer* OR electronic*) NEAR/3 (remind* OR alert*)):ti,ab OR ((econom* OR financ* OR regulatory OR dollar* OR cash OR money OR physician* OR provider* OR doctor* OR clinician* OR practitioner* OR nurse*) NEAR/3 (incentiv* OR reimburs*)):ti,ab OR ((worker* OR job OR jobs OR workplace* OR employe* OR student* OR school* OR daycare OR 'day care' OR pupil* OR child* OR infant* OR baby OR babies OR toddler*) NEAR/5 (keep* OR stay* OR remain*) NEAR/3 (home OR away)):ti,ab OR ((return* OR 'com* back') NEAR/5 (work* OR job OR jobs OR school* OR class OR daycare OR 'day care')):ti,ab

Note:

*Similar strategies were conducted in MEDLINE (EBSCOHost), The Cochrane Library (Wiley), CINHAL (EBSCOHost), PsychINFO (EBSCOHost) and Web of Science.

Appendix, Table 2. b) Search strategy in *LILACS.

LILACS Synthax: En Bireme, and IBECS, BINACIS, PAHO, PAHO-IRIS, LIS -Health Information Locator, MedCarib, CUMED, Sec. Est. Saúde SP

**Concept 1 - RTIs**
**(**"respiratory tract infection" **OR** respiratory tract infection$ **OR** "respiratory infections" **OR** "respiratory infection" **OR** "infecciones del sistema respiratorio" **OR** "infecciones respiratorias" **OR** "infección respiratoria" **OR** "infecciones del tracto respiratorio superior" **OR** "infecciones de las vias respiratorias superiores" **OR** "infecções respiratorias" **OR** "infecção respiratoria" **OR** "infecções do trato respiratório superior" **OR** "infecção do trato respiratório superior" **OR** "infecções das vias respiratórias superiores" **OR** "infecção das vias aereas superiores" **OR** "infecções das vias aereas superiores" **OR** "infecçõ do sistema respiratorio superior" **OR** "infecções do sistema respiratorio superior" **OR** "upper respiratory tract infections" **OR** "upper respiratory infections" **OR** rti **OR** urti ari **OR** arti **OR** urti **OR** lrti **OR** c01.539.739$ **OR** c08.730$ **OR** "common cold" **OR** common cold$ **OR** "resfriado común" **OR** "resfriado comum" **OR** catarrh$ **OR** coryza **OR** coriza **OR** "coriza aguda" **OR** "acute corzya" **OR** **((**nose$ **OR** nasal**)** **AND** **(**obstruction **OR** obstruccion **OR** obstrução **OR** runny **OR** running **OR** discharg$ **OR** congest$ **OR** block$ **OR** stuff$ **OR** dripping **OR** stuff$ **OR** nasosinusit$**))** **OR** c02.782.687.207$ **OR** c08.730.162$ **OR** cough **OR** cough$ **OR** acute cough$ **OR** tos$ **OR** "tos aguda" **OR** "tos subaguda" **OR** tosse$ **OR** "tosse aguda" **OR** "tosse subaguda" **OR** "subacute cough" **OR** c08.618.248$ **OR** c23.888.852.293$ **OR** sneezing **OR** sneez$ **OR** estornudo$ **OR** espirro$ **OR** c23.888.852.889$ **OR** g09.772.832 **OR** bronchitis **OR** bronchit$ **OR** bronchial infection$ **OR** bronquitis **OR** bronquite **OR** bronquit$ aguda **OR** c08.127.446$ **OR** c08.381.495.146$ **OR** c08.730.099$ **OR** "respiratory sounds" **OR** rales **OR** crackles **OR** rhonch **OR** tracheobronchit$ **OR** laryngotracheobronchit$ **OR** wheez$ **OR** "ruidos de la respiracion" **OR** "sonidos de la respiracion" **OR** "ruidos pulmonares" **OR** "ruidos del pulmon" **OR** "roce pleural" **OR** estertores **OR** ronquidos **OR** roncus **OR** sibilancia$ **OR** crepitacion **OR** "sonidos respiratorios" **OR** "ruidos respiratorios" **OR** "sons da respiração" **OR** "ruidos da respiração" **OR** "ruidos traqueobronquicos" **OR** "ruidos traqueo-bronquicos" **OR** "sons pulmonares" **OR** "atrito pleural" **OR** estertores **OR** roncos **OR** crepitação **OR** "estertor crepitante" **OR** bronchiolitis **OR** bronchiolit$ **OR** bronquiolitis **OR** bronquiolite **OR** c08.127.446.135$ **OR** c08.381.495.146.135$ **OR** c08.730.099.135$ **OR** "viral bronchiolitis" **OR** "bronchiolitis viral" **OR** "bronchiolite viral" **OR** "respiratory syncytial viruses" **OR** "respiratory syncytial virus" **OR** "respiratory syncytial viruses" **OR** "virus sincitiales respiratorios" **OR** "virus sincitial respiratorio" **OR** "virus sinciciais respiratorios" **OR** "virus sincicial respiratorio" **OR** "respiratory syncytial human virus" **OR** "respiratory syncytial human viruses" **OR** "respiratory syncytial virus infection" **OR** "respiratory syncytial virus infections" **OR** "otitis media" **OR** om **OR** "acute otitis media" **OR** aom **OR** "middle ear inflammation" **OR** "middle ear infection" **OR** "middle ear infections" **OR** "middle ear effusion" **OR** "serous otitis media" **OR** ome **OR** csom **OR** "glue ear" **OR** "glue ears" **OR** "otite média" **OR** "otite media aguda" **OR** "inflamação da orelha media" **OR** "inflamación del oído medio" **OR** "otitis media aguda" **OR** c09.218.705.663$ **OR** earache **OR** earaches **OR** "dolor de oído" **OR** "dor de orelha" **OR** otalgia **OR** "dor de ouvido" **OR** c09.218.350$ **OR** c23.888.592.612.302$ **OR** "laryngitis" **OR** "acute laryngitis" **OR** laryngit$ **OR** laringit$ **OR** pharyngolaryngit$ **OR** laryngotracheit$ **OR** laringopatias **OR** hoarseness **OR** ronquera **OR** rouquidão **OR** hoars$ **OR** **((**voice**)** **AND** **(**los$ **OR** disturb$ **OR** quality **OR** disorder$ **OR** croak$**))** **OR** **((**voz**)** **AND** **(**perdida **OR** perda **OR** calidad **OR** qualidade **OR** desorden$ **OR** ronc$ **OR** rouc$ **OR** disturb$ **OR** perturbação**))** **OR** croup **OR** pseudocroup **OR** "pseudo croup" **OR** crup **OR** crupe **OR** c08.360.535$ **OR** c08.730.368$ c09.400.535$ **OR** pharyngitis **OR** pharyngit$ **OR** acute pharyngit$ **OR** faringit$ **OR** sore throat$ **OR** acute sore throat$ **OR** "acute sore throat" **OR** "dor de garganta" **OR** "dolor de garganta" **OR** c07.550.781$ **OR** c08.730.561$ **OR** c09.775.649$ **OR** nasopharyngitis **OR** nasopharyngit$ **OR** nasofaringit$ **OR** rhinopharyngit$ **OR** rinofaringit$ **OR** c07.550.350.700$ **OR** c07.550.781.500$ c08.730.561.500$ **OR** c09.775.350.700$ **OR** c09.775.649.500$ **OR** tonsillitis **OR** tonsillit$ **OR** "acute tonsillitis" **OR** acute tonsillit$ **OR** tonsilit$ **OR** tonsilit$ aguda **OR** amigdalit$ **OR** amigdalit$ aguda **OR** "angina tonsillar" **OR** c07.550.781.750$ **OR** c08.730.561.750$ **OR** c09.775.649.750$ **OR** tracheitis **OR** traqueitis **OR** traqueite **OR** laryngotracheit$ **OR** c08.730.848$ **OR** c08.907.763$ **OR** rhinitis **OR** rinit$ **OR** rhinit$ **OR** nasal catarrh$ **OR** "acute rhinitis" **OR** rinit$ **OR** "catarro nasal" **OR** rhinosinusit$ **OR** nasosinusitis **OR** rhinovirus **OR** rhinovir$ **OR** rhinorrhea **OR** rhinorrhoea **OR** rinorrea **OR** rinorréia **OR** c08.460.799$ **OR** c08.730.674$ **OR** c09.603.799$ **OR** sinusitis **OR**

sinusit$ OR "acute sinusitis" OR acute sinutis$ OR c08.460.692.752$ OR c08.730.749$ OR c09.603.692.752$ OR ((infect$ OR infeccion$ OR infecçõ$ OR inflam$ OR inflamacion OR inflamação OR redness OR irritat$ OR edema) AND (laryng$ OR laring$ OR larynx OR epiglottit$ OR epiglotiti$ OR glottis OR glotis OR glote OR glot$ OR mucosa laring$ OR mucosa laryng$ OR "vocal cords" OR "pliegues vocales" OR "pregas vocais" OR throat OR pharyng$ OR faring$ OR garganta OR "nasal cavities" OR "cavidades nasals" OR "cavidades nasals" OR "cavidad nasal" OR tonsil$ OR amigdal$ OR tonsilas OR trachea OR traquea OR traqueia OR nasal mucos$ OR "mucosa nasal" OR "da mucosa nasal" OR "paranasal sinuses" OR "nos seios paranasais" OR "de los senos paranasales" OR sinusal$)) OR "human influenza" OR influenza$ OR grippe OR human flu$ OR flu$ OR "gripe humana" OR gripe OR gripa OR "gripa humana" OR "influenza humana" OR "influenza em humanos" OR "influenza en humanos" OR human parainfluenza OR parainfluenza$ humana OR ((infect$ OR infeccion$ OR infecçõ$) AND ("human parainfluenza" OR human parainfluenza virus OR "virus da parainfluenza humana" OR "virus de la parainfluenza humana")) OR c02.782.620.365$ OR c08.730.310$ OR pneumonia OR neumoní$ OR bronchopneumon$ OR pneumon$ OR pleuropneumon$ OR pleurisy OR pleuresia OR pleuritis OR pleurisia OR pleurisies OR pleuritides)
**Concept 2 - Antibiotics**
("anti-bacterial agents" OR antibiotic$ OR antibacterianos OR antibacterial$ OR anti-bacterial$ OR anti-biotic$ OR d27.505.954.122.085$ OR "anti-infective agents" OR antiinfective OR anti-infective$ OR "anti infective" OR antiinfeccioso$ OR anti-infeccioso$ OR "anti infecciosos" OR antimicrobial$ OR anti-microbial$ OR "anti microbial" OR ((agent$) AND (antiinfective OR anti-infective$ OR "anti infective" OR antiinfeccioso$ OR anti-infeccioso$ OR "anti infecciosos" OR antimicrobial$ OR anti-microbial$ OR "anti microbial")) OR penicillins OR penicil$ OR d02.065.589.099.750$ OR d02.886.108.750$ OR d03.438.260.825$ OR d03.605.084.737$ OR d04.075.080.875.099.221.750$ OR methicillin OR meticillin$ OR meticilin$ OR d02.065.589.099.750.500$ OR 02.886.108.750.500$ OR d03.633.100.300.750.500$ OR ampicillin OR ampicilin$ OR d02.065.589.099.750.750.050$ OR d02.886.108.750.750.050$ OR d03.633.100.300.750.750.050$ OR amoxicillin OR amoxicil$ OR amoxycillin$ OR d02.065.589.099.750.750.050.050$ OR d02.886.108.750.750.050.050$ OR d03.438.260.825.750.050.050$ OR d03.605.084.737.750.050.050$ OR d04.075.080.875.099.221.750.750.050.050$ OR tetracyclines OR tetra-cyclin$ OR tetraciclin$ OR d02.455.426.559.847.562.900$ OR d04.615.562.900$ OR azithromycin OR azithromycin$ OR azitromicin$ OR d02.540.576.500.992.050$ OR cephalexin OR cephalexin$ OR cefalexin$ OR d02.065.589.099.249.200$ OR d02.886.665.074.200$ OR d03.633.100.300.249.200$ OR macrolides OR macrolid$ OR d02.540.505$ OR d02.540.576.500$ OR d04.345.674.500$ OR quinolones OR quinolon$ OR fluoroquinolone$ OR d03.438.810.835$ OR ciprofloxacin OR ciprofloxacin$ OR d03.438.810.835.322.186$ OR cephalosporins OR cephalosporin$ OR cephalosporin$ OR d02.065.589.099.249$ OR d02.886.665.074$ OR d03.633.100.300.249$ OR beta-lactam$ OR "beta lactam" OR sulfonamide OR amantadine OR "amantadine sulfate" OR adamantanamine OR endantadine OR "amantadine sulfate" OR "amantadine sulphate" OR rimantadine OR rimantadin$ OR arbidol OR umifenovir OR zanamivir OR oseltamivir OR peramivir OR sulfonamide$ OR sulphonamid$ OR sulfamoyl OR enzil OR amantix OR amantrel OR amazolon OR aminoadamantane OR aminoadamantine OR atarin OR boidan OR hofcomant OR infectoflu OR mantadan OR mantadix OR mantidan OR midantane OR nurelin OR padiken OR paritrel OR parkintrel OR pk-merz OR prayanol OR protexin OR symadine OR symetrel OR symmetrel OR tregor OR viregyt OR virofral OR virosol OR virucid OR grippin OR flumadine OR flumandine OR gabirol OR germic OR remantadin OR remantadine OR roflual OR 'umifenovir' OR relenza OR oseltamivir OR tamiflu OR peramivir OR peramiflu OR rapiacta OR rapivab)
**Concept 3 - Prescribing**
("drug prescriptions" OR ((prescrip$ OR prescrib$ OR prescripcion$ OR prescriç$ OR use OR uso OR utiliza$) AND (de medicament$ OR treatment OR tratamiento OR therapy OR drug OR pharmac$ OR famac$)) OR "physician practice patterns" OR "padrões de pratica medica" OR "pautas de la práctica en medicina" OR ((prescrip$ OR prescrib$ OR prescripcion$ OR prescriç$ OR use OR uso OR utiliza$ OR utilisa$ OR giv$ OR issu$) AND (practice OR pattern$ OR padron OR practica OR pratica OR over OR abusiv$ OR excessive$ OR sobre OR inappropriate OR inadecuada OR inapropriada OR incorrecta OR irracional OR misuse OR mal uso OR improv$ OR appropriate$ OR proper OR racional OR correcta OR apropriada OR delay$ OR tardio OR atras$ OR postpon$ OR reduc$ OR discontinu$ OR stop$ OR restrict$ OR restringir OR limit$ OR banning OR banimento OR prohibicion OR overdos$ OR sobredos$ OR judicious$ OR juicioso OR judicioso OR prudent$ OR sensible OR reasonabl$ OR razonable$ OR razoavel OR necessary OR necesari$)) OR deprescrib$ OR deprescrip$ OR deprescriç$ OR stewardship OR campaign$

**OR** campaña$ **OR** campanha$ **OR** program$ **OR** strateg$ **OR** estrategia$ **OR** regulation$ **OR** regulatory **OR** regulacion **OR** regulamento**)**	
**Concept 4 - Setting**	
**(**"primary health care" **OR** "atencion primaria de salud" **OR** "atenção primaria a saude" **OR** "ambulatory care" **OR** "atención ambulatoria" **OR** "assistencia ambulatorial" **OR** "asistencia ambulatoria" **OR** "ambulatory care facilities" **OR** "instituciones de atencion ambulatoria" **OR** "instituiçoes de assistencia ambulatorial" **OR** "general practice" **OR** "medicina general" **OR** "medicina geral" **OR** "general practitioners" **OR** "medicos generales" **OR** "clinicos gerais" **OR** "family physicians" **OR** "medicos de familia" **OR** "primary care physicians" **OR** "médicos de atención primaria" **OR** "medicos de atenção primaria" **OR** "office visits" **OR** "visita a consultorio medico" **OR** "outpatients" **OR** "pacientes ambulatorios" **OR** "pacientes ambulatoriais" **OR** "paciente ambulatorio" **OR** "emergency medical services" **OR** "servicios medicos de urgencia" **OR** "serviços medicos de emergencia" **OR** "house calls" **OR** "visita domiciliaria" **OR** "visita domiciliar" **OR** pharmacists **OR** farmaceuticos **OR** pharmacist$ **OR** pharmacy **OR** farmacia **OR** "pharmacy technicians" **OR** "tecnicos de farmacia" **OR** "primary care" **OR** "primary health care" **OR** "primary healthcare" **OR ((**ambulatory **OR** ambulatori$ **OR** emergency**) AND (**care **OR** setting$ **OR** facilit$ **OR** ward$ **OR** department$ **OR** service$ **OR** ward$ **OR** atenção **OR** atencion **OR** assitencia **OR** asistencia **OR** servicio$ **OR** institution **OR** institucion **OR** instituiço**)) OR** practi$ **OR** physician$ **OR** doctor$ **OR** medico **OR** medico **OR** clinic$ **OR** professional$ **OR** professional$ **OR** farmac$ **OR** pharmac$ **OR** pratica$ **OR** professional$ **OR** after hour$ **OR** afterhour$ **OR** out of hour$ **OR** ooh **OR** outpatient$ **OR ((**clinic$ **OR** office**) AND (**visit$**)) OR ((**health$ **OR** medical**) AND (**center$ **OR** centre$**)))**	

Note:

LILACS = Latin American and Caribbean Literature on Health Sciences.

*Similar strategies were conducted in the databases Turning Research Into Practice database (TRIP), and the system for information on grey literature in Europe (http://opengrey.eu/).

List of excluded studies

N	Study reference	Reason for exclusion
1	Oppong R, Smith RD, Little P, Verheij T, Butler CC, Goossens H, et al. Cost-effectiveness of internet-based training for primary care clinicians on antibiotic prescribing for acute respiratory tract infections in Europe. J Antimicrob Chemother. 2018;73(11):3189-98.	cost-effectiveness modelling
2	Lubell Y, Do NTT, Nguyen KV, Ta NTD, Tran NTH, Than HM, et al. C-reactive protein point of care testing in the management of acute respiratory infections in the Vietnamese primary healthcare setting - a cost benefit analysis. Antimicrob Resist Infect Control. 2018;7:119.	cost-effectiveness modelling
3	Holmes EAF, Harris SD, Hughes A, Craine N, Hughes DA. Cost-Effectiveness Analysis of the Use of Point-of-Care C-Reactive Protein Testing to Reduce Antibiotic Prescribing in Primary Care. Antibiotics. 2018;7(4).	cost-effectiveness modelling
4	Minnaard MC, van de Pol AC, Hopstaken RM, van Delft S, Broekhuizen BDL, Verheij TJM, et al. C-reactive protein point-of-care testing and associated antibiotic prescribing. Fam Pract. 2016;33(4):408-13.	decision making analysis
5	Hughes A, Gwyn L, Harris S, Clarke C. Evaluating a point-of-care C-reactive protein test to support antibiotic prescribing decisions in a general practice. Clinical Pharmacist. 2016;8(10).	pilot; non-randomised
6	Huddy JR, Ni MZ, Barlow J, Majeed A, Hanna GB. Point-of-care C reactive protein for the diagnosis of lower respiratory tract infection in NHS primary care: a qualitative study of barriers and facilitators to adoption. BMJ Open. 2016;6(3):e009959.	qualitative study
7	Hunter R. Cost-Effectiveness of Point-of-Care C-Reactive Protein Tests for Respiratory Tract Infection in Primary Care in England. Advances in Therapy. 2015;32(1):69-85.	cost-effectiveness modelling
8	Anthierens S, Tonkin-Crine S, Cals JW, Coenen S, Yardley L, Brookes-Howell L, et al. Clinicians' views and experiences of interventions to enhance the quality of antibiotic prescribing for acute respiratory tract infections. J Gen Intern Med. 2015;30(4):408-16.	qualitative study
9	Nijman RG, Moll HA, Smit FJ, Gervaix A, Weerkamp F, Vergouwe Y, et al. C-reactive protein, procalcitonin and the lab-score for detecting serious bacterial infections in febrile children at the emergency department: a prospective observational study. Pediatr Infect Dis J. 2014;33(11):e273-9.	prospective observational study
10	Llor C, Cots JM, Hernández S, Ortega J, Arranz J, Monedero MJ, et al. Effectiveness of two types of intervention on antibiotic prescribing in respiratory tract infections in Primary Care in Spain. Happy Audit Study. Atencion Primaria. 2014;46(9):492-500.	non-randomised; no trial
11	Llor C, Bjerrum L, Munck A, Cots JM, Hernández S, Moragas A. Access to point-of-care tests reduces the prescription of antibiotics among antibiotic-requesting subjects with respiratory tract infections. Respiratory Care. 2014;59(12):1918-23.	non-randomised; no trial; no comparator
12	Lacroix L, Manzano S, Vandertuin L, Hugon F, Galetto-Lacour A, Gervaix A. Impact of the lab-score on antibiotic prescription rate in children with fever without source: a randomized controlled trial. PLoS One. 2014;9(12):e115061.	no comparator of interest
13	ClinicalTrials.gov [Internet]. Identifier: NCT04470518. Validation of a vital signs and symptoms decision tree and the effect of a point-of-care C-reactive protein test, oxygen saturation, a brief intervention and a parent leaflet on diagnosing, antibiotic prescribing rate and parental satisfaction in acutely ill children in primary care; 2014 Dec [cited 2020 Sep 08]. Available at: https://clinicaltrials.gov/ct2/show/NCT02024282?cond=NCT02024282&draw=2&rank=1.	validation study
14	Peters CM, Schouwenaars FM, Haagsma E, Evenhuis HM, Echteld MA. Antibiotic prescribing and C-reactive protein testing for pulmonary infections in patients with intellectual disabilities. Br J Gen Pract. 2013;63(610):e326-e30.	non-randomised; no trial

15	Oppong R, Jit M, Smith RD, Butler CC, Melbye H, Mölstad S, et al. Cost-effectiveness of point-of-care C-reactive protein testing to inform antibiotic prescribing decisions. Br J Gen Pract. 2013;63(612):e465-e71.	cost-effectiveness modelling
16	Llor C, Hernández S, Cots JM, Bjerrum L, González B, García G, et al. [Physicians with access to point-of-care tests significantly reduce the antibiotic prescription for common cold]. Rev Esp Quimioter. 2013;26(1):12-20.	non-randomised; no trial
17	Joshi A, Perin DP, Gehle A, Nsiah-Kumi PA. Feasibility of using C-reactive protein for point-of-care testing. Technol Health Care. 2013;21(3):233-40.	feasibility study; no trial
18	Llor C, Cots JM, López-Valcárcel BG, Arranz J, García G, Ortega J, et al. Interventions to reduce antibiotic prescription for lower respiratory tract infections: Happy Audit study. Eur Respir J. 2012;40(2):436-41.	non-randomised; no trial
19	Llor C, Bjerrum L, Arranz J, García G, Cots JM, González López-Valcárcel B, et al. C-reactive protein testing in patients with acute rhinosinusitis leads to a reduction in antibiotic use. Fam Pract. 2012;29(6):653-8.	non-randomised; no trial
20	Anthierens S, Tonkin-Crine S, Douglas E, Fernandez-Vandellos P, Krawczyk J, Llor C, et al. General practitioners' views on the acceptability and applicability of a web-based intervention to reduce antibiotic prescribing for acute cough in multiple European countries: a qualitative study prior to a randomised trial. BMC Fam Pract. 2012;13:9.	qualitative study; no trial
21	Kavanagh KE, O'Shea E, Halloran R, Cantillon P, Murphy AW. A pilot study of the use of near-patient C-Reactive Protein testing in the treatment of adult respiratory tract infections in one Irish general practice. BMC Fam Pract. 2011;12:93.	pilot; non-randomised
22	Cals JWL, Ament AJHA, Hood K, Butler CC, Hopstaken RM, Wassink GF, et al. C-reactive protein point of care testing and physician communication skills training for lower respiratory tract infections in general practice: economic evaluation of a cluster randomized trial. J Eval Clin Pract. 2011;17(6):1059-69.	cost-effectiveness modelling
23	Llor C, Sierra N, Hernández S, Moragas A, Hernández M, Bayona C, et al. Impact of C-reactive protein testing on adherence to thrice-daily antibiotic regimens in patients with lower respiratory tract infection. Prim Care Respir J. 2010;19(4):358-62.	non-randomised; no trial
24	Jakobsen KA, Melbye H, Kelly MJ, Ceynowa C, Molstad S, Hood K, et al. Influence of CRP testing and clinical findings on antibiotic prescribing in adults presenting with acute cough in primary care. Scand J Prim Health Care. 2010;28(4):229-36.	non-randomised; no trial
25	Cals JWL, Chappin FHF, Hopstaken RM, van Leeuwen ME, Hood K, Butler CC, et al. C-reactive protein point-of-care testing for lower respiratory tract infections: a qualitative evaluation of experiences by GPs. Fam Pract. 2010;27(2):212-8.	qualitative; non-randomised
26	Rausch S, Flammang M, Haas N, Stein R, Tabouring P, Delvigne S, et al. C-reactive protein to initiate or withhold antibiotics in acute respiratory tract infections in adults, in primary care: review. Bull Soc Sci Med Grand Duche Luxemb. 2009(1):79-87.	review
27	Cals JWL, Butler CC, Dinant GJ. 'Experience talks': physician prioritisation of contrasting interventions to optimise management of acute cough in general practice. Implement Sci. 2009;4:6.	qualitative; non-randomised
28	Cohen R, Lécuyer A, Wollner C, Deberdt P, Thollot F, Henriquet V, et al. [Evaluation of impact of CRP rapid test in management of febrile children in ambulatory pediatric practice]. Arch Pediatr. 2008;15(6):1126-32.	non-randomised; no comparator
29	Muszyńska A, Steciwko A, Horst-Sikorska W, Siebert J, Mastalerz-Migas A, Wawrzyniak A, et al. Usefulness of rapid CRP tests (NycoCard II® CRP) in everyday work of a family doctor, in the aspect of rationalization of indications for antibiotic therapy in acute infections. Fam Med Prim Care Rev. 2007;9(4):998-1006.	non-randomised; no comparator with POCT
30	Briel M, Young J, Tschudi P, Hersberger KE, Hugenschmidt C, Langewitz W, et al. Prevalence and influence of diagnostic tests for acute respiratory tract infections in primary care. Swiss Med Wkly. 2006;136(15-16):248-53.	non-randomised; no comparator

31	Bjerrum L, Gahrn-Hansen B, Munck AP. [General practitioners who use CRP have a lower antibiotic prescribing rate to patients with sinusitis - secondary publication]. Ugeskr Laeger. 2005;167(25-31):2775-7.	non-randomised; no trial
32	Bjerrum L, Gahrn-Hansen B, Munck AP. C-reactive protein measurement in general practice may lead to lower antibiotic prescribing for sinusitis. Br J Gen Pract. 2004;54(506):659-62.	non-randomised; no trial
33	Fagan MS. Can the use of antibiotic in the treatment of acute bronchitis be reduced? [Kan bruk av antibiotika ved akutt bronkitt reduseres?]. Tidsskr Nor Laegeforen. 2001;121(4):455-8.	non-randomised
34	Dahler-Eriksen BS, Lauritzen T, Lassen JF, Lund ED, Brandslund I. Near-patient test for C-reactive protein in general practice: assessment of clinical, organizational, and economic outcomes. Clin Chem. 1999;45(4):478-85.	no relevant comparator; crp effect can't be assessed exclusively

Figures: risk of bias

Appendix, Figure 1. Graph for the risk of bias assessment across all included RCTs.

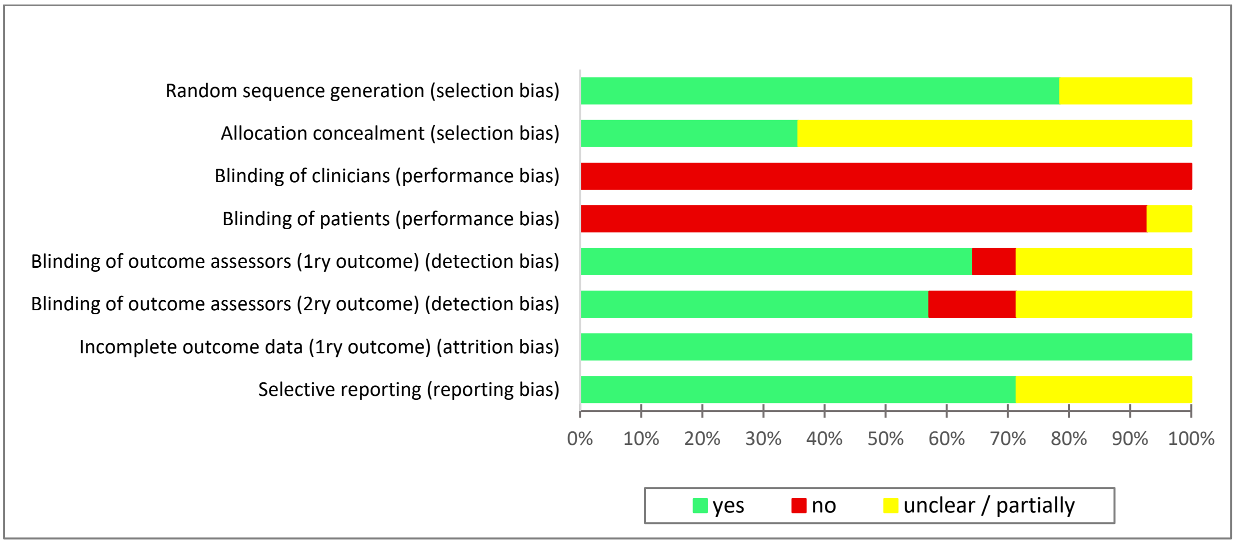

Figures: primary outcomes

Appendix, Figure 2. Antibiotic prescribing at index consultations by type of RTIs.

Study or Subgroup	CRP Events	CRP Total	Usual Care Events	Usual Care Total	Weight	Risk Ratio M-H, Random, 95% CI
**2.4.1 Individual and Cluster (modified sample) RCTs - Upper RTIs**						
Little, 2013	52	190	83	185	7.0%	0.61 [0.46, 0.81]
Cals, 2010	33	73	47	78	6.4%	0.75 [0.55, 1.02]
**Subtotal (95% CI)**		**263**		**263**	**13.4%**	**0.67 [0.54, 0.82]**
Total events	85		130			
Heterogeneity: Tau² = 0.00; Chi² = 0.96, df = 1 (P = 0.33); I² = 0%						
Test for overall effect: Z = 3.77 (P = 0.0002)						
**2.4.2 Individual and Cluster (modified sample) RCTs - Lower RTIs**						
Little, 2013	215	590	288	462	10.0%	0.58 [0.51, 0.66]
Cals, 2009	20	65	31	59	4.5%	0.59 [0.38, 0.91]
Andreeva, 2014	18	49	22	38	4.3%	0.63 [0.40, 1.00]
Schot, 2018	42	136	65	165	6.3%	0.78 [0.57, 1.07]
Cals, 2010	23	56	26	51	4.8%	0.81 [0.53, 1.22]
Melbye, 1995	54	108	68	131	7.6%	0.96 [0.75, 1.24]
**Subtotal (95% CI)**		**1004**		**906**	**37.5%**	**0.72 [0.58, 0.88]**
Total events	372		500			
Heterogeneity: Tau² = 0.04; Chi² = 14.54, df = 5 (P = 0.01); I² = 66%						
Test for overall effect: Z = 3.17 (P = 0.002)						
**2.4.3 Individual and Cluster (modified sample) RCTs - Undefined RTIs**						
Takemura, 2005	61	106	122	134	9.2%	0.63 [0.53, 0.75]
Do, 2016	441	1017	647	1019	10.7%	0.68 [0.63, 0.74]
Verbakel, 2016	117	455	106	381	8.1%	0.92 [0.74, 1.16]
Diederichsen, 2000	179	414	183	398	9.5%	0.94 [0.81, 1.10]
Gonzales, 2011	25	69	19	62	3.9%	1.18 [0.73, 1.93]
Rebnord, 2016	36	138	57	259	5.5%	1.19 [0.82, 1.70]
Van den Bruel, 2016	10	26	9	28	2.2%	1.20 [0.58, 2.47]
**Subtotal (95% CI)**		**2225**		**2281**	**49.2%**	**0.87 [0.72, 1.05]**
Total events	869		1143			
Heterogeneity: Tau² = 0.04; Chi² = 32.13, df = 6 (P < 0.0001); I² = 81%						
Test for overall effect: Z = 1.48 (P = 0.14)						
**Total (95% CI)**		**3492**		**3450**	**100.0%**	**0.78 [0.69, 0.87]**
Total events	1326		1773			
Heterogeneity: Tau² = 0.03; Chi² = 52.07, df = 14 (P < 0.00001); I² = 73%						
Test for overall effect: Z = 4.17 (P < 0.0001)						
Test for subgroup differences: Chi² = 3.61, df = 2 (P = 0.16), I² = 44.5%						

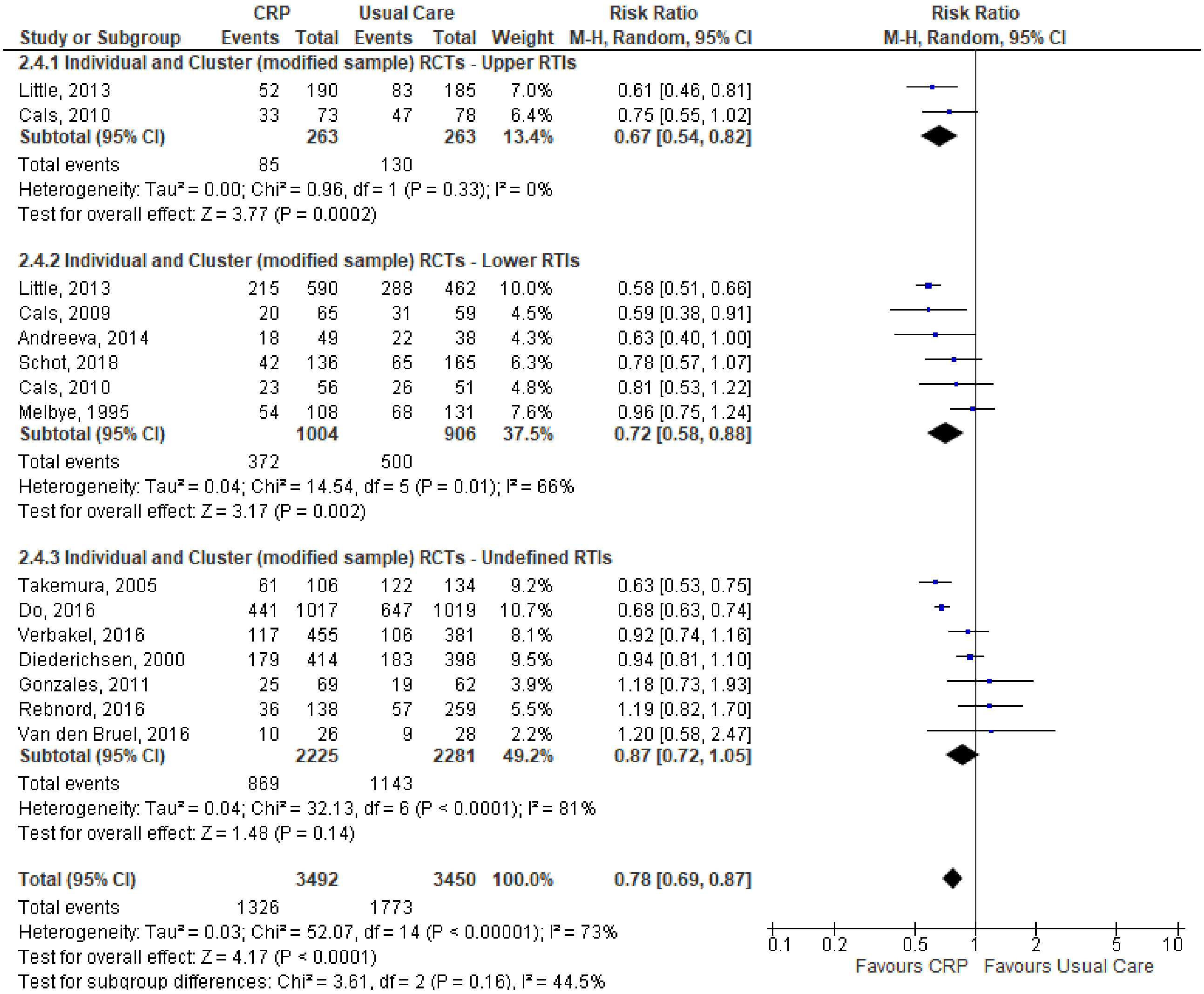

Note. CRP, C-Reactive Protein (Point Of Care Test); RCTs, Randomised Controlled Trials; M-H, Mantel-Haenszel; CI, Confidence Interval; df, degrees of freedom; I², heterogeneity between trials.

Appendix, Figure 3. a) Antibiotic prescribing at index consultations: CRP (cut-off) guidance applied versus not applied to withhold antibiotics.

Study or Subgroup	CRP Events	CRP Total	Usual Care Events	Usual Care Total	Weight	Risk Ratio M-H, Random, 95% CI
**4.1.1 Individual and Cluster RCTs (modified sample) - CRP (cut-off) guidance**						
Cals, 2009 (<20 mg/L)	20	65	31	59	5.1%	0.59 [0.38, 0.91]
Little, 2013 (≤20 mg/L)	230	665	318	544	11.2%	0.59 [0.52, 0.67]
Andreeva, 2014 (<20 mg/L)	18	49	22	38	4.8%	0.63 [0.40, 1.00]
Do, 2016 (≤10 or ≤20 mg/L)	441	1017	647	1019	11.9%	0.68 [0.63, 0.74]
Cals, 2010 (<20 mg/L)	56	129	73	129	8.5%	0.77 [0.60, 0.98]
Verbakel, 2016 (<5 mg/L)	117	455	106	381	9.0%	0.92 [0.74, 1.16]
Melbye, 1995 (<11 mg/L)	54	108	68	131	8.5%	0.96 [0.75, 1.24]
Gonzales, 2011 (<10 mg/L)	25	69	19	62	4.4%	1.18 [0.73, 1.93]
**Subtotal (95% CI)**		**2557**		**2363**	**63.3%**	**0.75 [0.65, 0.87]**
Total events	961		1284			
Heterogeneity: Tau² = 0.03; Chi² = 25.05, df = 7 (P = 0.0007); I² = 72%						
Test for overall effect: Z = 3.80 (P = 0.0001)						
**4.1.2 Individual RCTs - No CRP (cut-off) guidance***						
Takemura, 2005	61	106	122	134	10.2%	0.63 [0.53, 0.75]
Schot, 2018	42	136	65	165	7.1%	0.78 [0.57, 1.07]
Diederichsen, 2000	179	414	183	398	10.6%	0.94 [0.81, 1.10]
Rebnord, 2016	36	138	57	259	6.2%	1.19 [0.82, 1.70]
Van den Bruel, 2016	10	26	9	28	2.5%	1.20 [0.58, 2.47]
**Subtotal (95% CI)**		**820**		**984**	**36.7%**	**0.87 [0.68, 1.12]**
Total events	328		436			
Heterogeneity: Tau² = 0.06; Chi² = 17.95, df = 4 (P = 0.001); I² = 78%						
Test for overall effect: Z = 1.06 (P = 0.29)						
**Total (95% CI)**		**3377**		**3347**	**100.0%**	**0.79 [0.70, 0.90]**
Total events	1289		1720			
Heterogeneity: Tau² = 0.03; Chi² = 49.59, df = 12 (P < 0.00001); I² = 76%						
Test for overall effect: Z = 3.59 (P = 0.0003)						
Test for subgroup differences: Chi² = 0.96, df = 1 (P = 0.33), I² = 0%						

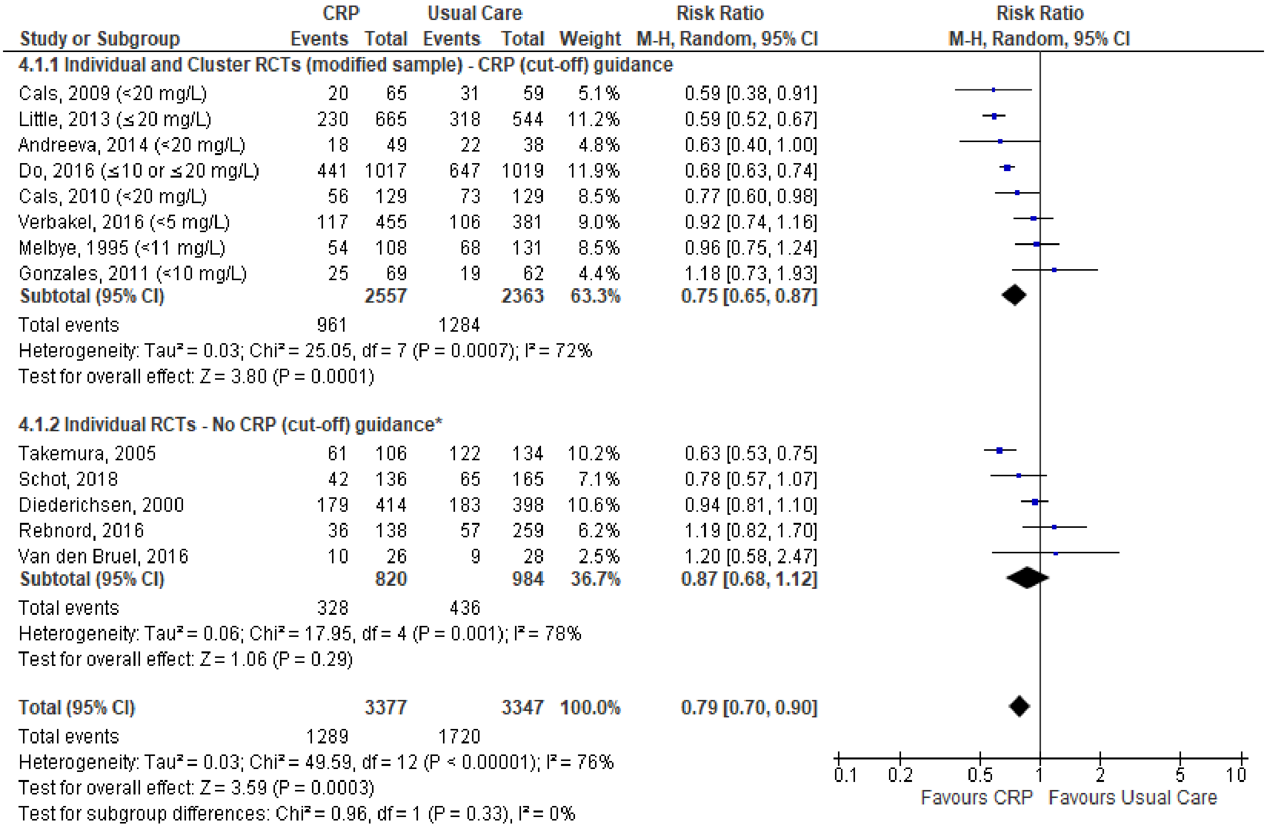

Note. CRP, C-Reactive Protein (Point Of Care Test); RCTs, Randomised Controlled Trials; M-H, Mantel-Haenszel; CI, Confidence Interval; df, degrees of freedom; I², heterogeneity between trials. CRP values recommended to withhold antibiotics are presented in brackets.

* CRP (cut-off) guidance was not clear or not applied to withhold antibiotics

Appendix, Figure 3. b) Antibiotic prescribing at index consultations: CRP (cut-off) guidance applied by age groups versus not applied to withhold antibiotics.

Study or Subgroup	CRP Events	CRP Total	Usual Care Events	Usual Care Total	Weight	Risk Ratio M-H, Random, 95% CI
**4.5.1 Individual and Cluster (modified sample) RCTs - Adults**						
Cals, 2009 (<20 mg/L)	20	65	31	59	4.7%	0.59 [0.38, 0.91]
Little, 2013 (≤20 mg/L)	230	665	318	544	10.6%	0.59 [0.52, 0.67]
Andreeva, 2014 (<20 mg/L)	18	49	22	38	4.5%	0.63 [0.40, 1.00]
Do, 2016 (≤20 mg/L)	214	507	314	501	10.7%	0.67 [0.60, 0.76]
Cals, 2010 (<20 mg/L)	56	129	73	129	8.0%	0.77 [0.60, 0.98]
Melbye, 1995 (<11 mg/L)	54	108	68	131	8.0%	0.96 [0.75, 1.24]
Gonzales, 2011 (<10 mg/L)	25	69	19	62	4.1%	1.18 [0.73, 1.93]
**Subtotal (95% CI)**		**1592**		**1464**	**50.5%**	**0.72 [0.62, 0.85]**
Total events	617		845			
Heterogeneity: Tau² = 0.03; Chi² = 18.48, df = 6 (P = 0.005); I² = 68%						
Test for overall effect: Z = 3.96 (P < 0.0001)						
**4.5.2 Individual and Cluster (modified sample) RCTs - Children**						
Verbakel, 2016 (<5 mg/L)	19	260	76	422	4.2%	0.41 [0.25, 0.65]
Do, 2016 (≤10 mg/L)	227	510	333	518	10.8%	0.69 [0.62, 0.78]
**Subtotal (95% CI)**		**770**		**940**	**15.0%**	**0.56 [0.33, 0.95]**
Total events	246		409			
Heterogeneity: Tau² = 0.12; Chi² = 4.87, df = 1 (P = 0.03); I² = 79%						
Test for overall effect: Z = 2.15 (P = 0.03)						
**4.5.3 Individual RCTs - No CRP (cut-off) guidance***						
Takemura, 2005	61	106	122	134	9.7%	0.63 [0.53, 0.75]
Schot, 2018	42	136	65	165	6.7%	0.78 [0.57, 1.07]
Diederichsen, 2000	179	414	183	398	10.1%	0.94 [0.81, 1.10]
Rebnord, 2016	36	138	57	259	5.8%	1.19 [0.82, 1.70]
Van den Bruel, 2016	10	26	9	28	2.3%	1.20 [0.58, 2.47]
**Subtotal (95% CI)**		**820**		**984**	**34.5%**	**0.87 [0.68, 1.12]**
Total events	328		436			
Heterogeneity: Tau² = 0.06; Chi² = 17.95, df = 4 (P = 0.001); I² = 78%						
Test for overall effect: Z = 1.06 (P = 0.29)						
**Total (95% CI)**		**3182**		**3388**	**100.0%**	**0.75 [0.66, 0.84]**
Total events	1191		1690			
Heterogeneity: Tau² = 0.03; Chi² = 50.10, df = 13 (P < 0.00001); I² = 74%						
Test for overall effect: Z = 4.69 (P < 0.00001)						
Test for subgroup differences: Chi² = 2.71, df = 2 (P = 0.26), I² = 26.1%						

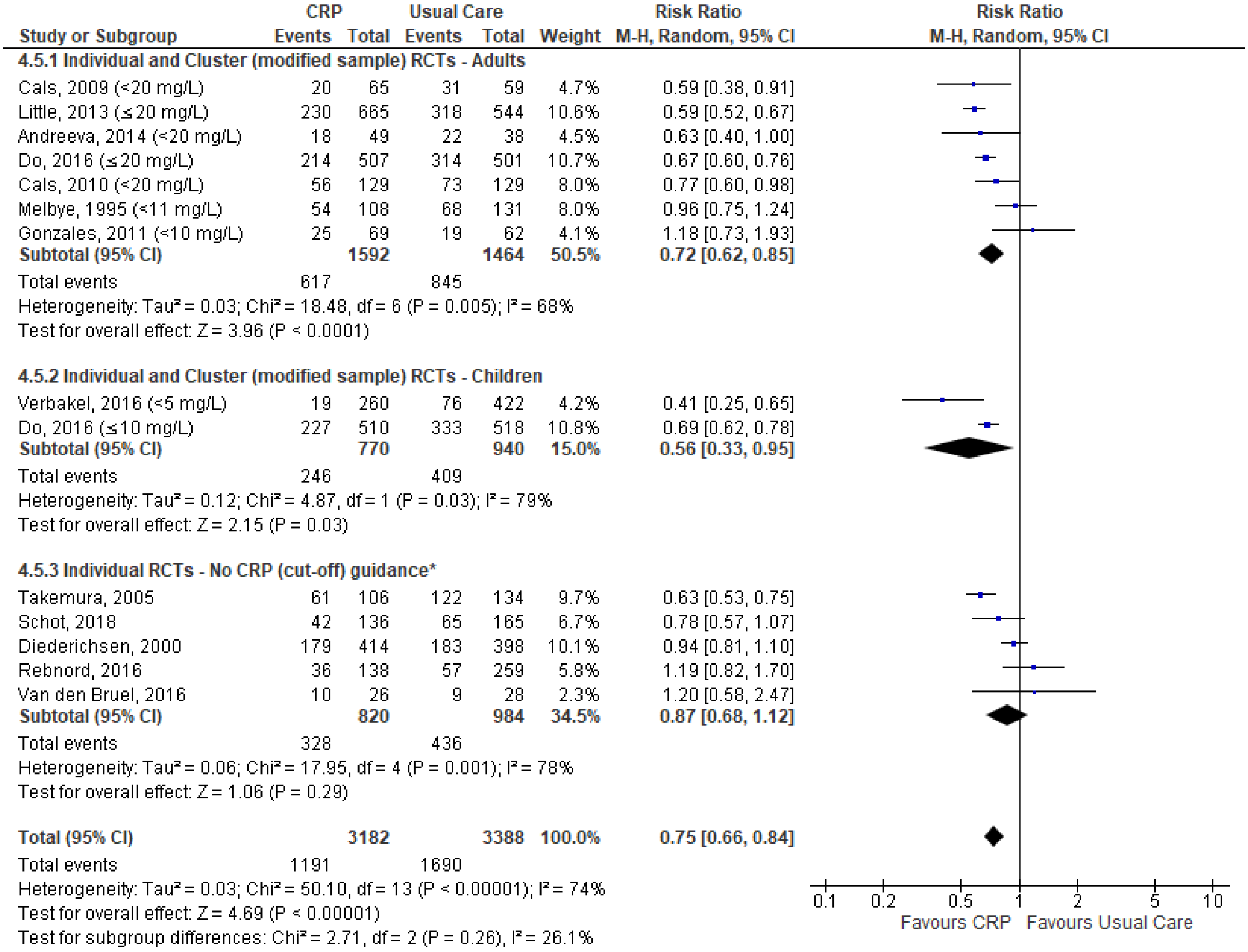

Note. CRP, C-Reactive Protein (Point Of Care Test); RCTs, Randomised Controlled Trials; M-H, Mantel-Haenszel; CI, Confidence Interval; df, degrees of freedom; I², heterogeneity between trials. CRP values recommended to withhold antibiotics are presented in brackets.

* CRP (cut-off) guidance was not clear or not applied to withhold antibiotics

Appendix, Figure 4. Antibiotic prescribing at index consultations by Healthcare Settings.

Study or Subgroup	CRP Events	CRP Total	Usual Care Events	Usual Care Total	Weight	Risk Ratio M-H, Random, 95% CI
**5.1.1 Individual and Cluster (modified sample) RCTs - GPs**						
Cals, 2009	20	65	31	59	5.0%	0.59 [0.38, 0.91]
Little, 2013	230	665	318	544	11.0%	0.59 [0.52, 0.67]
Andreeva, 2014	18	49	22	38	4.7%	0.63 [0.40, 1.00]
Cals, 2010	56	129	73	129	8.4%	0.77 [0.60, 0.98]
Verbakel, 2016	117	455	106	381	8.9%	0.92 [0.74, 1.16]
Diederichsen, 2000	179	414	183	398	10.4%	0.94 [0.81, 1.10]
Melbye, 1995	54	108	68	131	8.3%	0.96 [0.75, 1.24]
**Subtotal (95% CI)**		**1885**		**1680**	**56.6%**	**0.77 [0.64, 0.94]**
Total events	674		801			
Heterogeneity: Tau² = 0.05; Chi² = 30.80, df = 6 (P < 0.0001); I² = 81%						
Test for overall effect: Z = 2.59 (P = 0.009)						
**5.1.2 Individual RCTs - Outpatient (hospitals and health-centres) services**						
Takemura, 2005	61	106	122	134	10.0%	0.63 [0.53, 0.75]
Do, 2016	441	1017	647	1019	11.7%	0.68 [0.63, 0.74]
**Subtotal (95% CI)**		**1123**		**1153**	**21.8%**	**0.67 [0.62, 0.73]**
Total events	502		769			
Heterogeneity: Tau² = 0.00; Chi² = 0.64, df = 1 (P = 0.42); I² = 0%						
Test for overall effect: Z = 10.27 (P < 0.00001)						
**5.1.3 Individual RCTs - Other (OOH, OHH+GPs, Ped ED, ED) services**						
Schot, 2018	42	136	65	165	7.0%	0.78 [0.57, 1.07]
Rebnord, 2016 (Ped ED)	11	59	19	108	2.7%	1.06 [0.54, 2.07]
Gonzales, 2011	25	69	19	62	4.3%	1.18 [0.73, 1.93]
Van den Bruel, 2016	10	26	9	28	2.4%	1.20 [0.58, 2.47]
Rebnord, 2016 (OOH)	25	79	38	151	5.1%	1.26 [0.82, 1.92]
**Subtotal (95% CI)**		**369**		**514**	**21.6%**	**1.00 [0.81, 1.24]**
Total events	113		150			
Heterogeneity: Tau² = 0.00; Chi² = 4.13, df = 4 (P = 0.39); I² = 3%						
Test for overall effect: Z = 0.03 (P = 0.97)						
**Total (95% CI)**		**3377**		**3347**	**100.0%**	**0.80 [0.70, 0.90]**
Total events	1289		1720			
Heterogeneity: Tau² = 0.03; Chi² = 50.19, df = 13 (P < 0.00001); I² = 74%						
Test for overall effect: Z = 3.53 (P = 0.0004)						
Test for subgroup differences: Chi² = 13.14, df = 2 (P = 0.001), I² = 84.8%						

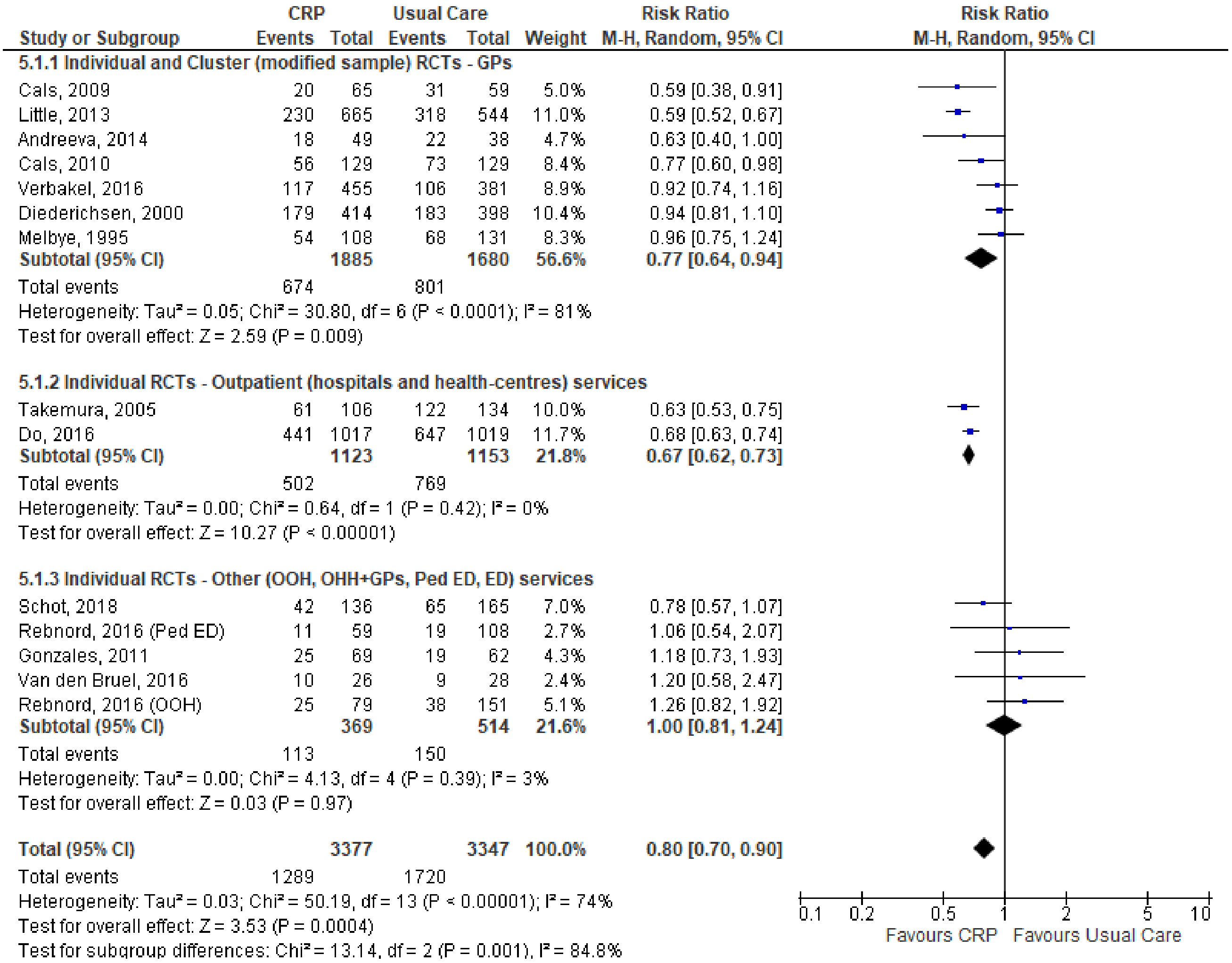

Note. CRP, C-Reactive Protein (Point Of Care Test); RCTs, Randomised Controlled Trials; M-H, Mantel-Haenszel; CI, Confidence Interval; df, degrees of freedom; I², heterogeneity between trials. OOH = Out-Of-Hours, GP = General Practice, Ped = Paediatric, ED = Emergency Department. Schot, 2018: OOH+GP; Rebnord, 2016: mutually exclusive data for OOH and Ped ED; Gonzales, 2011: ED; Van den Bruel: OOH.

Appendix, Figure 5. a) Antibiotic prescribing at any point during 28 days follow-up.

Study or Subgroup	CRP Events	CRP Total	Usual Care Events	Usual Care Total	Weight	Risk Ratio M-H, Random, 95% CI
**7.1.1 Individual RCTs**						
Cals, 2010	68	129	84	129	23.6%	0.81 [0.66, 1.00]
Schot, 2018	6	81	8	99	2.8%	0.92 [0.33, 2.53]
Melbye, 1995	61	108	78	131	22.8%	0.95 [0.76, 1.18]
Do, 2016	140	461	91	300	22.6%	1.00 [0.80, 1.25]
Van den Bruel, 2016	6	17	1	19	0.8%	6.71 [0.90, 50.22]
**Subtotal (95% CI)**		**796**		**678**	**72.5%**	**0.93 [0.78, 1.10]**
Total events	281		262			
Heterogeneity: Tau² = 0.01; Chi² = 6.08, df = 4 (P = 0.19); I² = 34%						
Test for overall effect: Z = 0.86 (P = 0.39)						
**7.1.2 Cluster RCTs (modified sample)**						
Andreeva, 2014	20	49	27	38	12.7%	0.57 [0.39, 0.85]
Cals, 2009	29	65	34	59	14.8%	0.77 [0.55, 1.10]
**Subtotal (95% CI)**		**114**		**97**	**27.5%**	**0.68 [0.51, 0.91]**
Total events	49		61			
Heterogeneity: Tau² = 0.01; Chi² = 1.24, df = 1 (P = 0.27); I² = 19%						
Test for overall effect: Z = 2.63 (P = 0.009)						
**Total (95% CI)**		**910**		**775**	**100.0%**	**0.85 [0.72, 1.02]**
Total events	330		323			
Heterogeneity: Tau² = 0.02; Chi² = 11.56, df = 6 (P = 0.07); I² = 48%						
Test for overall effect: Z = 1.76 (P = 0.08)						
Test for subgroup differences: Chi² = 3.33, df = 1 (P = 0.07), I² = 69.9%						

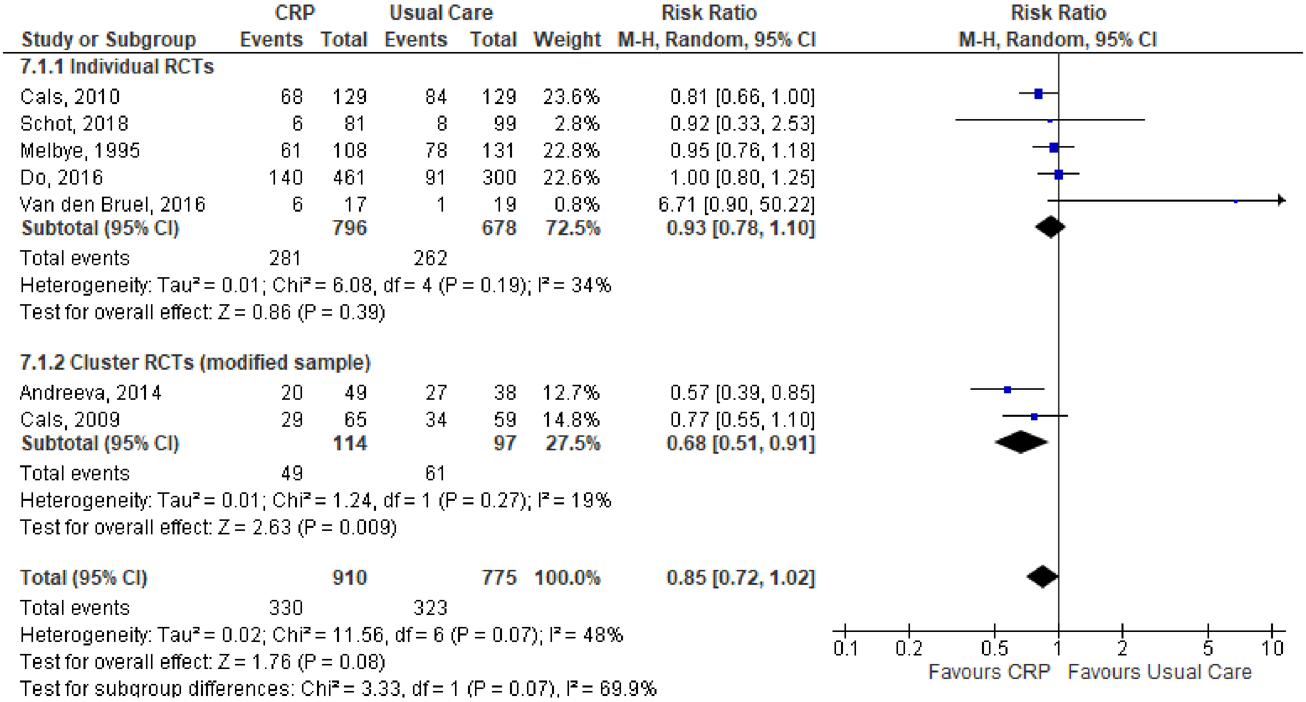

Appendix, Figure 5. b) Antibiotic prescribing at any point during 28 days follow-up by RTI type.

Study or Subgroup	CRP Events	CRP Total	Usual Care Events	Usual Care Total	Weight	Risk Ratio M-H, Random, 95% CI
**7.2.1 Individual RCTs - Upper RTIs**						
Cals, 2010	42	73	54	78	68.5%	0.83 [0.65, 1.06]
**Subtotal (95% CI)**		**73**		**78**	**68.5%**	**0.83 [0.65, 1.06]**
Total events	42		54			
Heterogeneity: Not applicable						
Test for overall effect: Z = 1.47 (P = 0.14)						
**7.2.2 Individual RCTs - Lower RTIs**						
Cals, 2010	26	56	30	51	31.5%	0.79 [0.55, 1.13]
**Subtotal (95% CI)**		**56**		**51**	**31.5%**	**0.79 [0.55, 1.13]**
Total events	26		30			
Heterogeneity: Not applicable						
Test for overall effect: Z = 1.28 (P = 0.20)						
**Total (95% CI)**		**129**		**129**	**100.0%**	**0.82 [0.67, 1.00]**
Total events	68		84			
Heterogeneity: Tau² = 0.00; Chi² = 0.05, df = 1 (P = 0.82); I² = 0%						
Test for overall effect: Z = 1.94 (P = 0.05)						
Test for subgroup differences: Chi² = 0.05, df = 1 (P = 0.82), I² = 0%						

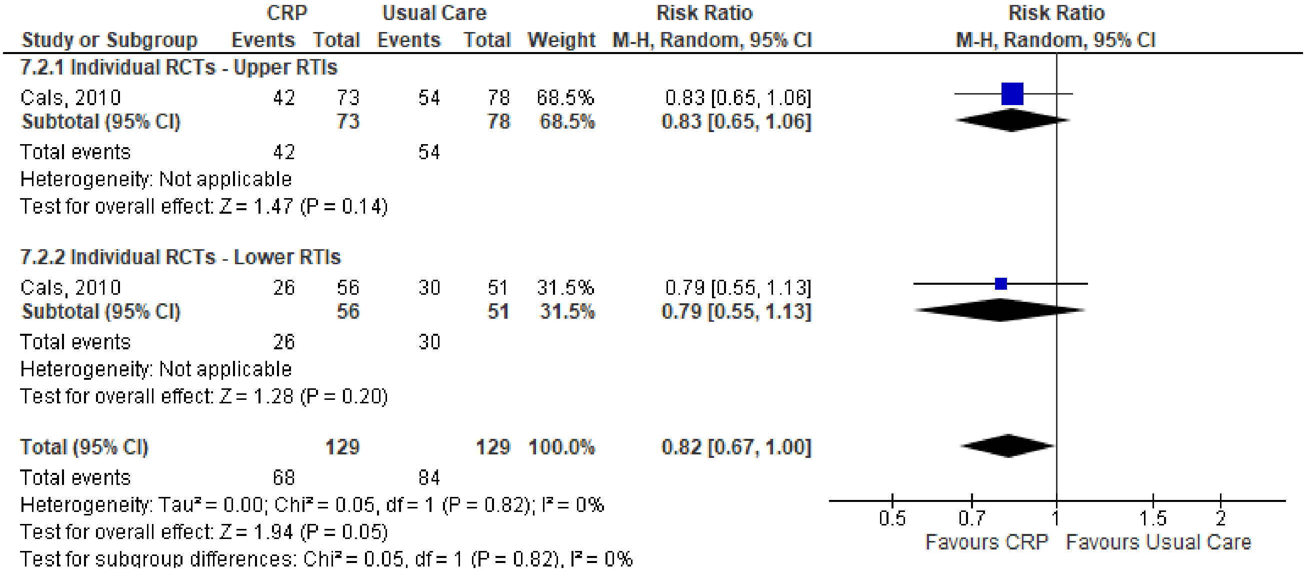

Note. CRP, C-Reactive Protein (Point Of Care Test); RCTs, Randomised Controlled Trials; M-H, Mantel-Haenszel; CI, Confidence Interval; df, degrees of freedom; I2, heterogeneity between trials.

Appendix, Figure 5. c) CRP-POCT compared with Usual Care. Antibiotic prescribing within 3 months.

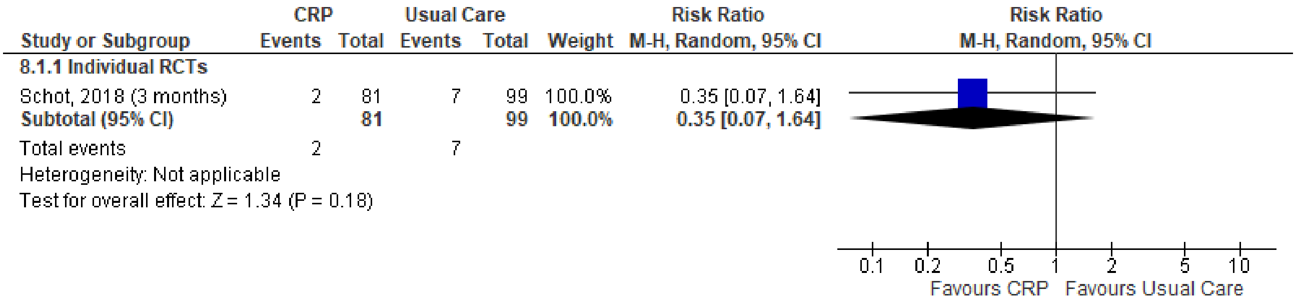

Appendix, Figure 5. d) CRP-POCT compared with Usual Care. Antibiotic prescribing at 12 months follow-up.

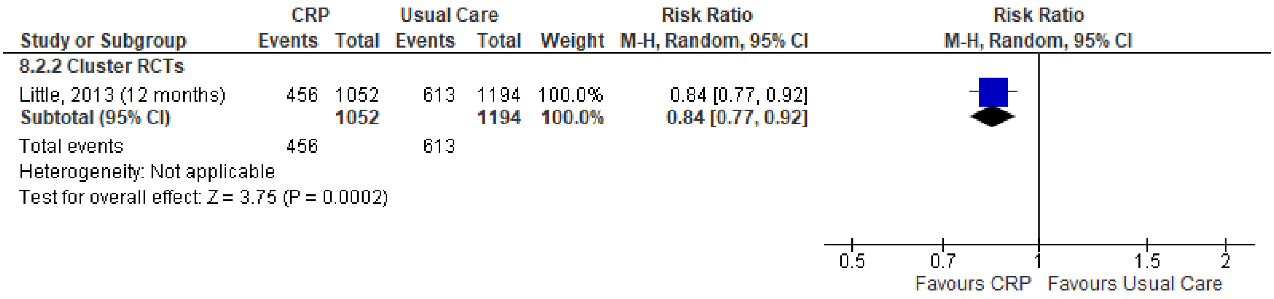

Appendix, Figure 5. e) CRP-POCT compared with Usual Care. Antibiotic prescribing at 12 months follow-up by RTI type.

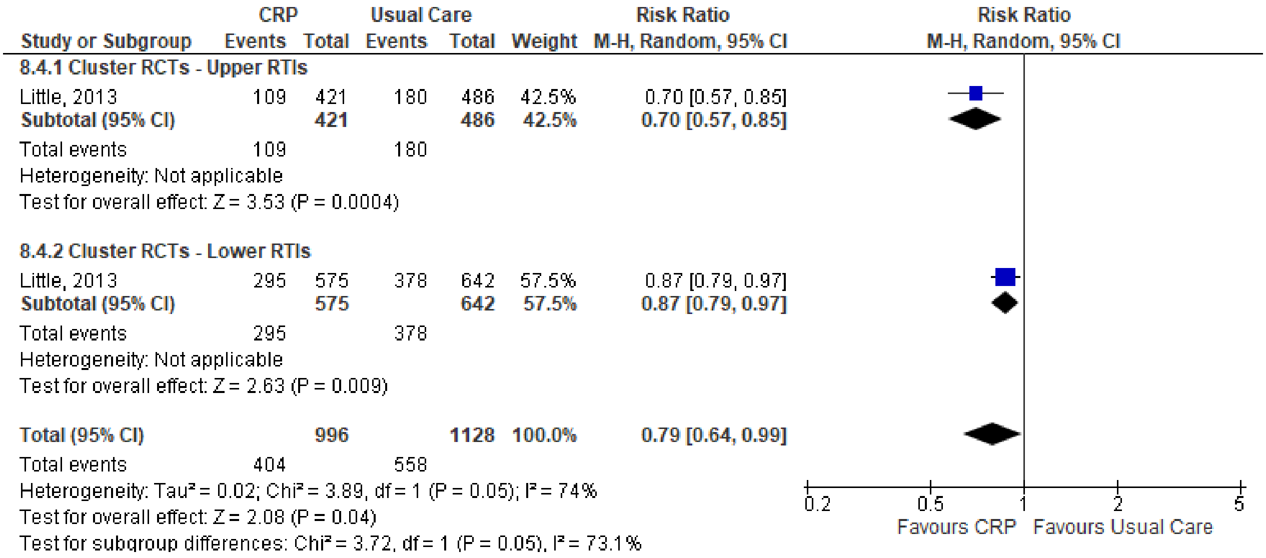

Note. CRP, C-Reactive Protein (Point Of Care Test); RCTs, Randomised Controlled Trials; M-H, Mantel-Haenszel; CI, Confidence Interval; df, degrees of freedom; I^2, heterogeneity between trials.

Appendix, Figure 5. f) CRP-POCT compared with Usual Care. Antibiotic prescribing at follow-up: within 3.5 years.

Study or Subgroup	CRP Mean	CRP SD	CRP Total	Usual Care Mean	Usual Care SD	Usual Care Total	Weight	Mean Difference IV, Random, 95% CI
**8.3.1 Cluster RCTs**								
Cals, 2009 (3.5 years)	0.307	0.4987	294	0.357	0.6159	373	100.0%	-0.05 [-0.13, 0.03]
**Subtotal (95% CI)**			**294**			**373**	**100.0%**	**-0.05 [-0.13, 0.03]**
Heterogeneity: Not applicable								
Test for overall effect: Z = 1.16 (P = 0.25)								

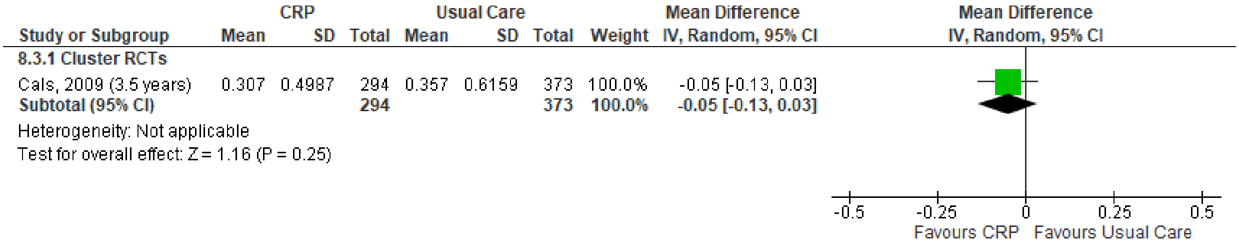

Figures: secondary outcomes

Appendix, Figure 6. a) Clinical recovery within 7 days.

Study or Subgroup	CRP Events	CRP Total	Usual Care Events	Usual Care Total	Weight	Risk Ratio (Non-event) M-H, Random, 95% CI
**9.1.1 Individual RCTs**						
Melbye, 1995 (1)	46	102	53	128	19.4%	0.94 [0.75, 1.18]
Cals, 2010 (2)	27	118	31	125	51.1%	1.03 [0.89, 1.18]
Diederichsen, 2000 (3)	251	407	252	384	29.5%	1.12 [0.93, 1.34]
**Subtotal (95% CI)**		**627**		**637**	**100.0%**	**1.03 [0.93, 1.14]**
Total events	324		336			
Heterogeneity: Tau² = 0.00; Chi² = 1.43, df = 2 (P = 0.49); I² = 0%						
Test for overall effect: Z = 0.63 (P = 0.53)						

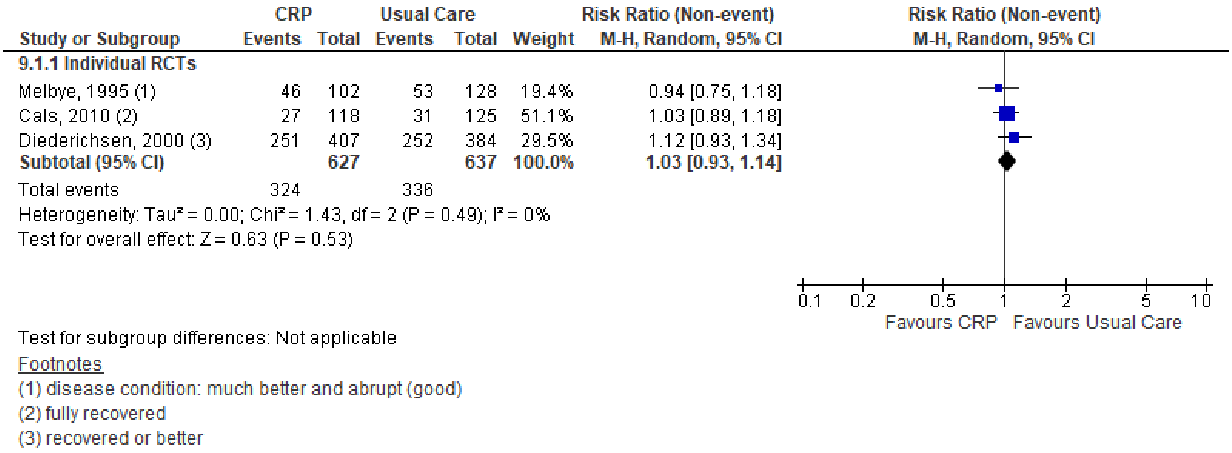

Test for subgroup differences: Not applicable

Footnotes

(1) disease condition: much better and abrupt (good)

(2) fully recovered

(3) recovered or better

Appendix, Figure 6. b) Clinical recovery within 28 days.

Study or Subgroup	CRP Events	CRP Total	Usual Care Events	Usual Care Total	Weight	Risk Ratio (Non-event) M-H, Random, 95% CI
**9.2.1 Individual RCTs**						
Melbye, 1995	71	98	82	121	55.1%	0.85 [0.57, 1.29]
**Subtotal (95% CI)**		**98**		**121**	**55.1%**	**0.85 [0.57, 1.29]**
Total events	71		82			
Heterogeneity: Not applicable						
Test for overall effect: Z = 0.75 (P = 0.46)						
**9.2.2 Cluster RCTs (modified sample)**						
Cals, 2009	76	102	69	91	38.6%	1.05 [0.64, 1.73]
Andreeva, 2014	60	66	47	51	6.4%	1.16 [0.35, 3.89]
**Subtotal (95% CI)**		**168**		**142**	**44.9%**	**1.07 [0.68, 1.69]**
Total events	136		116			
Heterogeneity: Tau² = 0.00; Chi² = 0.02, df = 1 (P = 0.89); I² = 0%						
Test for overall effect: Z = 0.29 (P = 0.78)						
**Total (95% CI)**		**266**		**263**	**100.0%**	**0.95 [0.70, 1.28]**
Total events	207		198			
Heterogeneity: Tau² = 0.00; Chi² = 0.53, df = 2 (P = 0.77); I² = 0%						
Test for overall effect: Z = 0.36 (P = 0.72)						
Test for subgroup differences: Chi² = 0.51, df = 1 (P = 0.48), I² = 0%						

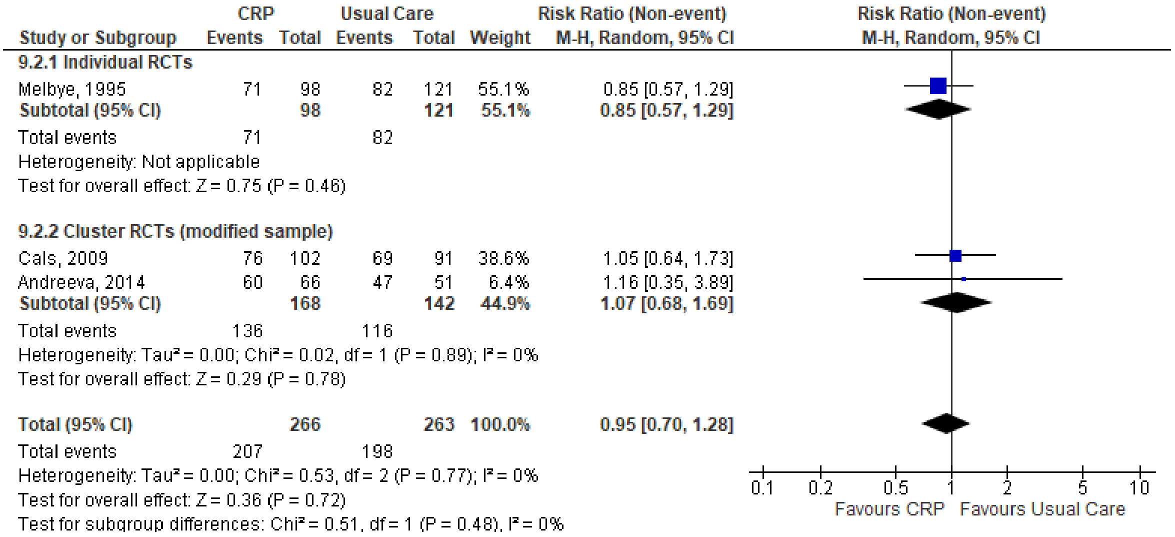

Appendix, Figure 7. Time in mean number of days to resolution of symptoms.

Study or Subgroup	CRP Mean	CRP SD	CRP Total	Usual Care Mean	Usual Care SD	Usual Care Total	Weight	Mean Difference IV, Random, 95% CI
**9.3.1 Individual RCTs**								
Do, 2016	5.33	2.23	1017	5.33	2.23	1019	100.0%	0.00 [-0.19, 0.19]
**Subtotal (95% CI)**			**1017**			**1019**	**100.0%**	**0.00 [-0.19, 0.19]**
Heterogeneity: Not applicable								
Test for overall effect: Z = 0.00 (P = 1.00)								
**9.3.2 Cluster RCTs**								
Little, 2013	5.33	3.71	1018	5	2.97	861	100.0%	0.33 [0.03, 0.63]
**Subtotal (95% CI)**			**1018**			**861**	**100.0%**	**0.33 [0.03, 0.63]**
Heterogeneity: Not applicable								
Test for overall effect: Z = 2.14 (P = 0.03)								

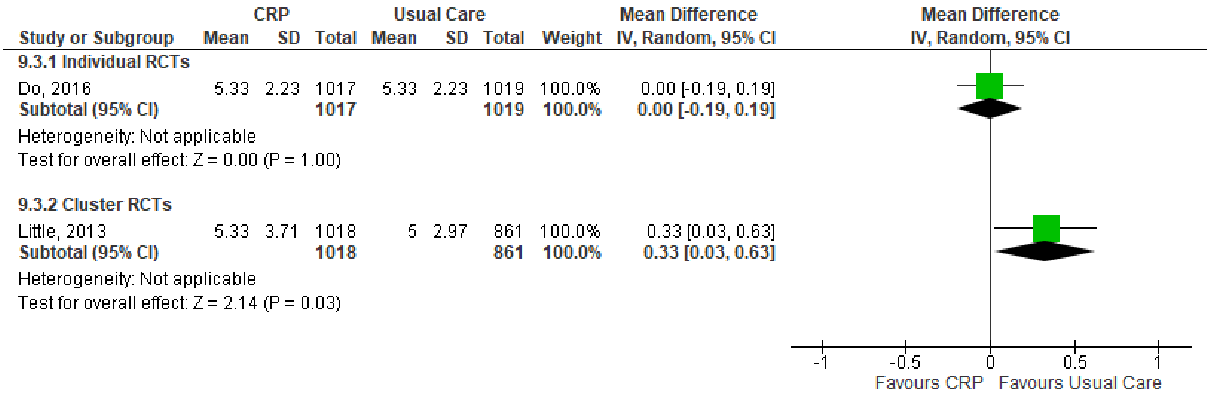

Appendix, Figure 8. Re-consultations within 30 days.

Study or Subgroup	CRP Events	CRP Total	Usual Care Events	Usual Care Total	Weight	Risk Ratio M-H, Random, 95% CI
**12.1.1 Individual RCTs**						
Schot, 2018	27	81	34	99	14.5%	0.97 [0.64, 1.46]
Gonzales, 2011	28	69	20	62	11.6%	1.26 [0.79, 1.99]
Cals, 2010	33	129	23	129	11.0%	1.43 [0.89, 2.30]
Do, 2016	5	1017	3	1019	1.2%	1.67 [0.40, 6.97]
Van den Bruel, 2016	9	17	6	19	3.9%	1.68 [0.75, 3.73]
**Subtotal (95% CI)**		**1313**		**1328**	**42.3%**	**1.23 [0.97, 1.57]**
Total events	102		86			
Heterogeneity: Tau² = 0.00; Chi² = 2.45, df = 4 (P = 0.65); I² = 0%						
Test for overall effect: Z = 1.69 (P = 0.09)						
**12.1.2 Cluster RCTs (modified sample)**						
Andreeva, 2014	3	93	3	72	1.0%	0.77 [0.16, 3.72]
Cals, 2009	66	188	51	169	26.2%	1.16 [0.86, 1.57]
Little, 2013	129	636	64	538	30.5%	1.71 [1.29, 2.25]
**Subtotal (95% CI)**		**917**		**779**	**57.7%**	**1.38 [0.98, 1.94]**
Total events	198		118			
Heterogeneity: Tau² = 0.04; Chi² = 3.99, df = 2 (P = 0.14); I² = 50%						
Test for overall effect: Z = 1.85 (P = 0.06)						
**Total (95% CI)**		**2230**		**2107**	**100.0%**	**1.33 [1.14, 1.57]**
Total events	300		204			
Heterogeneity: Tau² = 0.00; Chi² = 7.22, df = 7 (P = 0.41); I² = 3%						
Test for overall effect: Z = 3.55 (P = 0.0004)						
Test for subgroup differences: Chi² = 0.28, df = 1 (P = 0.60), I² = 0%						

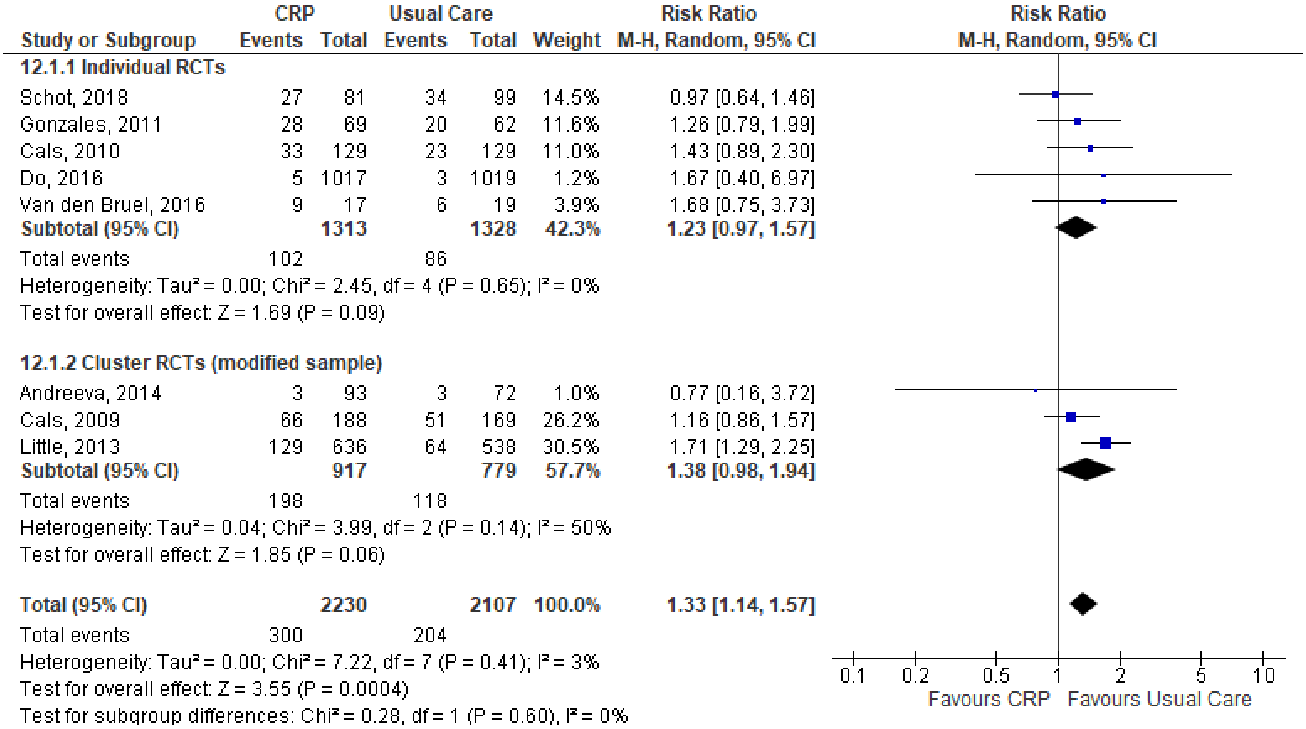

Appendix, Figure 9. Intention to re-consult.

Study or Subgroup	CRP Events	CRP Total	Usual Care Events	Usual Care Total	Weight	Risk Ratio M-H, Random, 95% CI
**12.2.1 Individual RCTs**						
Cals, 2010	86	115	75	123	100.0%	1.23 [1.03, 1.46]
**Subtotal (95% CI)**		**115**		**123**	**100.0%**	**1.23 [1.03, 1.46]**
Total events	86		75			
Heterogeneity: Not applicable						
Test for overall effect: Z = 2.26 (P = 0.02)						
**12.2.2 Cluster RCTs**						
Cals, 2009	153	227	138	204	100.0%	1.00 [0.87, 1.14]
**Subtotal (95% CI)**		**227**		**204**	**100.0%**	**1.00 [0.87, 1.14]**
Total events	153		138			
Heterogeneity: Not applicable						
Test for overall effect: Z = 0.05 (P = 0.96)						

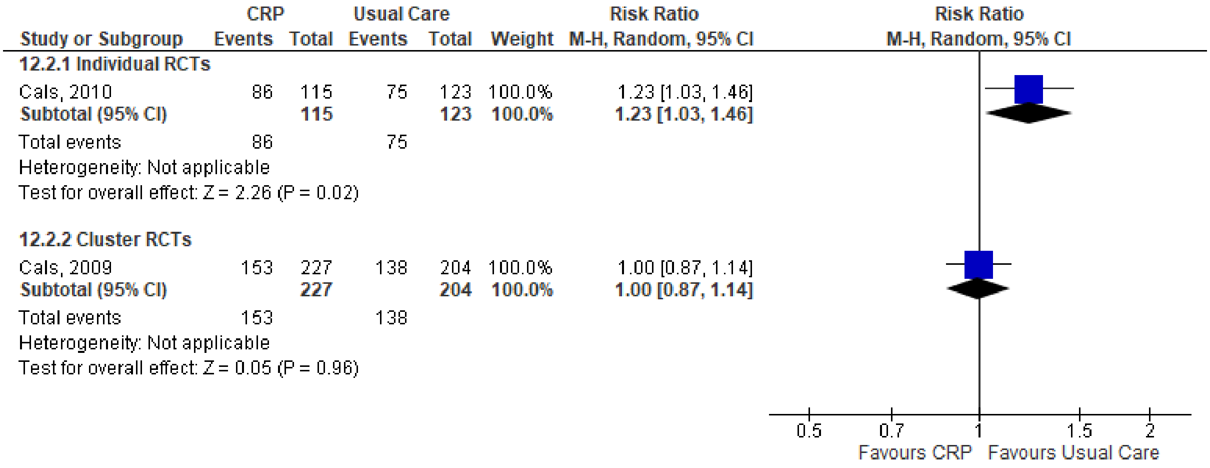

Appendix, Figure 10. Referrals to Hospital.

Study or Subgroup	CRP Events	CRP Total	Usual Care Events	Usual Care Total	Weight	Risk Ratio M-H, Random, 95% CI
**13.1.1 Individual RCTs**						
Rebnord, 2016	7	138	24	259	46.3%	0.55 [0.24, 1.24]
Van den Bruel, 2016	3	26	4	28	19.1%	0.81 [0.20, 3.27]
**Subtotal (95% CI)**		**164**		**287**	**65.4%**	**0.60 [0.30, 1.22]**
Total events	10		28			
Heterogeneity: Tau² = 0.00; Chi² = 0.22, df = 1 (P = 0.64); I² = 0%						
Test for overall effect: Z = 1.40 (P = 0.16)						
**13.1.2 Cluster RCTs (modified sample)**						
Verbakel, 2016	11	566	6	470	34.6%	1.52 [0.57, 4.09]
**Subtotal (95% CI)**		**566**		**470**	**34.6%**	**1.52 [0.57, 4.09]**
Total events	11		6			
Heterogeneity: Not applicable						
Test for overall effect: Z = 0.83 (P = 0.40)						
**Total (95% CI)**		**730**		**757**	**100.0%**	**0.84 [0.44, 1.61]**
Total events	21		34			
Heterogeneity: Tau² = 0.06; Chi² = 2.45, df = 2 (P = 0.29); I² = 18%						
Test for overall effect: Z = 0.53 (P = 0.60)						
Test for subgroup differences: Chi² = 2.23, df = 1 (P = 0.14), I² = 55.1%						

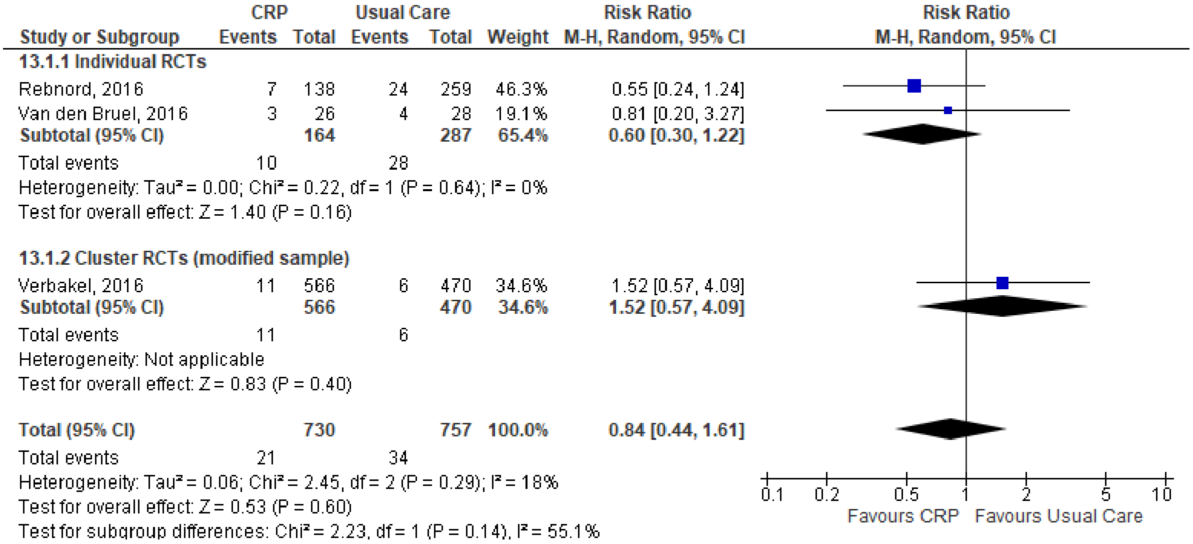

Appendix, Figure 11. Admissions to Hospital.

Study or Subgroup	CRP Events	CRP Total	Usual Care Events	Usual Care Total	Weight	Risk Ratio M-H, Random, 95% CI
**14.1.1 Individual RCTs**						
Schot, 2018	0	136	1	165	3.4%	0.40 [0.02, 9.84]
Do, 2016	6	901	8	874	31.5%	0.73 [0.25, 2.09]
Gonzales, 2011	4	69	2	62	12.7%	1.80 [0.34, 9.47]
**Subtotal (95% CI)**		**1106**		**1101**	**47.6%**	**0.89 [0.38, 2.09]**
Total events	10		11			
Heterogeneity: Tau² = 0.00; Chi² = 1.06, df = 2 (P = 0.59); I² = 0%						
Test for overall effect: Z = 0.27 (P = 0.78)						
**14.1.4 Cluster RCTs (modified sample)**						
Verbakel, 2016	12	649	8	532	44.5%	1.23 [0.51, 2.99]
Little, 2013	6	645	1	545	7.8%	5.07 [0.61, 41.98]
**Subtotal (95% CI)**		**1294**		**1077**	**52.4%**	**1.80 [0.52, 6.24]**
Total events	18		9			
Heterogeneity: Tau² = 0.35; Chi² = 1.51, df = 1 (P = 0.22); I² = 34%						
Test for overall effect: Z = 0.92 (P = 0.36)						
**Total (95% CI)**		**2400**		**2178**	**100.0%**	**1.18 [0.65, 2.13]**
Total events	28		20			
Heterogeneity: Tau² = 0.00; Chi² = 3.35, df = 4 (P = 0.50); I² = 0%						
Test for overall effect: Z = 0.54 (P = 0.59)						
Test for subgroup differences: Chi² = 0.84, df = 1 (P = 0.36), I² = 0%						

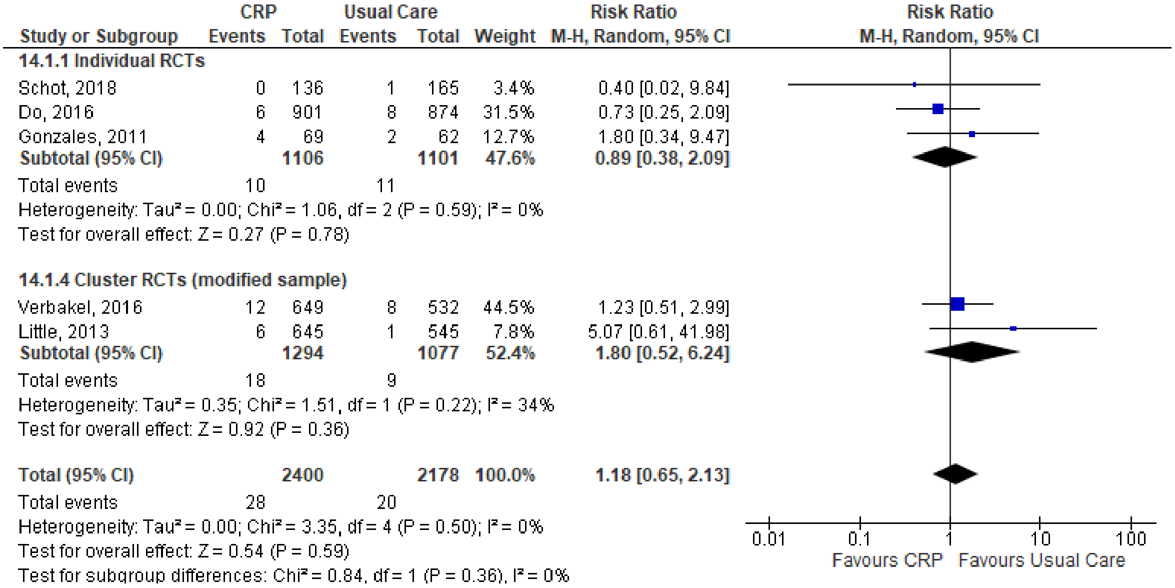

Appendix, Figure 12. a) Ordering of Investigations: additional tests.

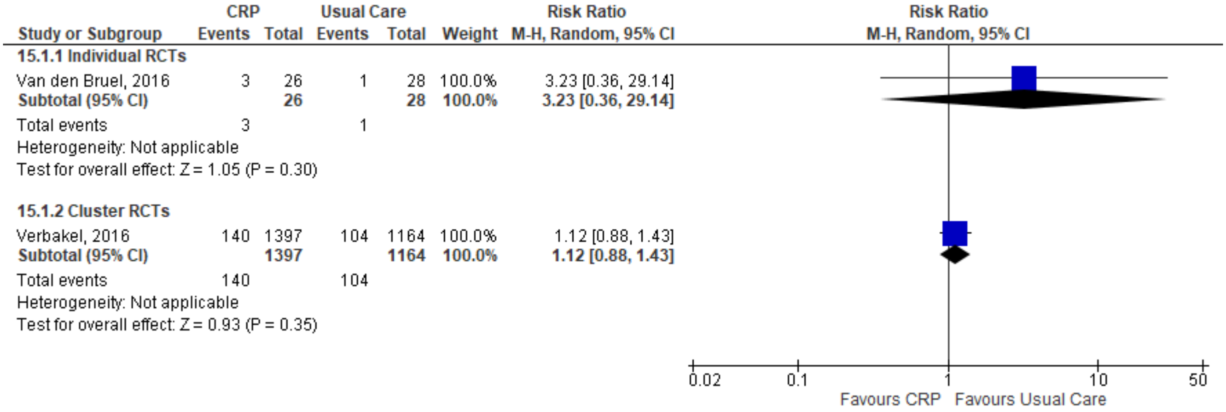

Appendix, Figure 12. b) Ordering of Investigations: chest x-rays.

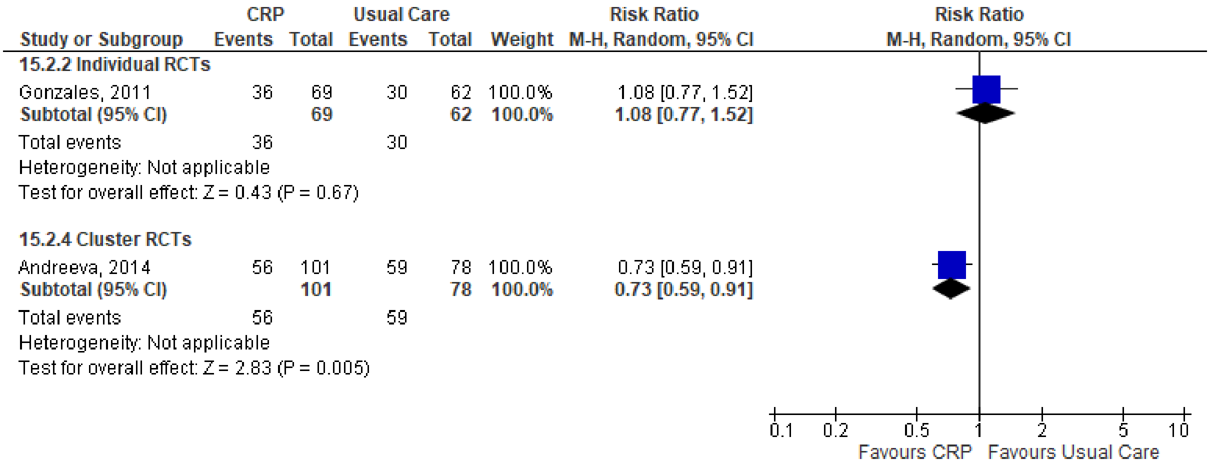

Appendix, Figure 13. Patient Satisfaction.

Study or Subgroup	CRP Events	CRP Total	Usual Care Events	Usual Care Total	Weight	Risk Ratio (Non-event) M-H, Random, 95% CI
**16.1.1 Individual RCTs**						
Cals, 2010	90	118	79	125	42.4%	0.64 [0.43, 0.96]
Do, 2016	545	549	541	542	3.1%	3.95 [0.44, 35.22]
**Subtotal (95% CI)**		**667**		**667**	**45.5%**	**1.15 [0.22, 6.17]**
Total events	635		620			
Heterogeneity: Tau² = 1.04; Chi² = 2.61, df = 1 (P = 0.11); I² = 62%						
Test for overall effect: Z = 0.17 (P = 0.87)						
**16.1.2 Cluster RCTs (modified sample)**						
Cals, 2009	159	227	136	204	54.5%	0.90 [0.68, 1.19]
**Subtotal (95% CI)**		**227**		**204**	**54.5%**	**0.90 [0.68, 1.19]**
Total events	159		136			
Heterogeneity: Not applicable						
Test for overall effect: Z = 0.75 (P = 0.45)						
**Total (95% CI)**		**894**		**871**	**100.0%**	**0.82 [0.55, 1.21]**
Total events	794		756			
Heterogeneity: Tau² = 0.05; Chi² = 3.82, df = 2 (P = 0.15); I² = 48%						
Test for overall effect: Z = 1.01 (P = 0.31)						
Test for subgroup differences: Chi² = 0.08, df = 1 (P = 0.77), I² = 0%						

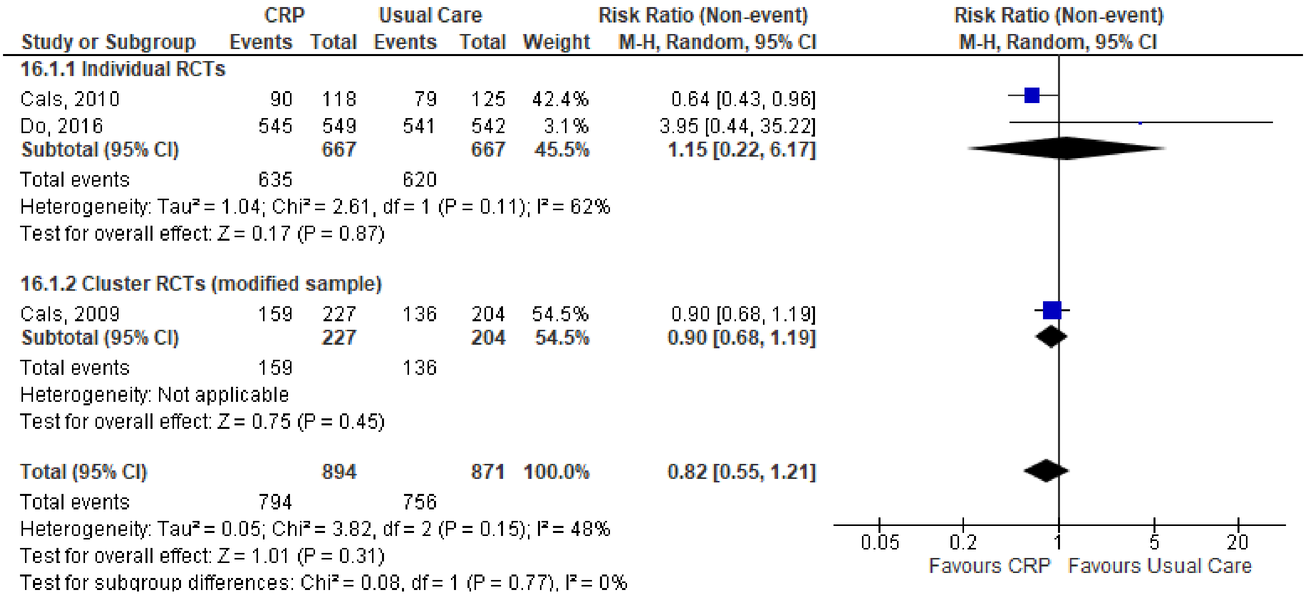

Appendix, Figure 14. Patient Enablement.

Study or Subgroup	CRP Mean	CRP SD	CRP Total	Usual Care Mean	Usual Care SD	Usual Care Total	Weight	Mean Difference IV, Random, 95% CI
**17.1.1 Individual RCTs**								
Cals, 2010	2.5	2.6	117	2.3	2.4	125	100.0%	0.20 [-0.43, 0.83]
**Subtotal (95% CI)**			**117**			**125**	**100.0%**	**0.20 [-0.43, 0.83]**
Heterogeneity: Not applicable								
Test for overall effect: Z = 0.62 (P = 0.53)								
**17.1.2 Cluster RCTs**								
Cals, 2009	2.97	2.59	227	3.4	2.48	204	100.0%	-0.43 [-0.91, 0.05]
**Subtotal (95% CI)**			**227**			**204**	**100.0%**	**-0.43 [-0.91, 0.05]**
Heterogeneity: Not applicable								
Test for overall effect: Z = 1.76 (P = 0.08)								

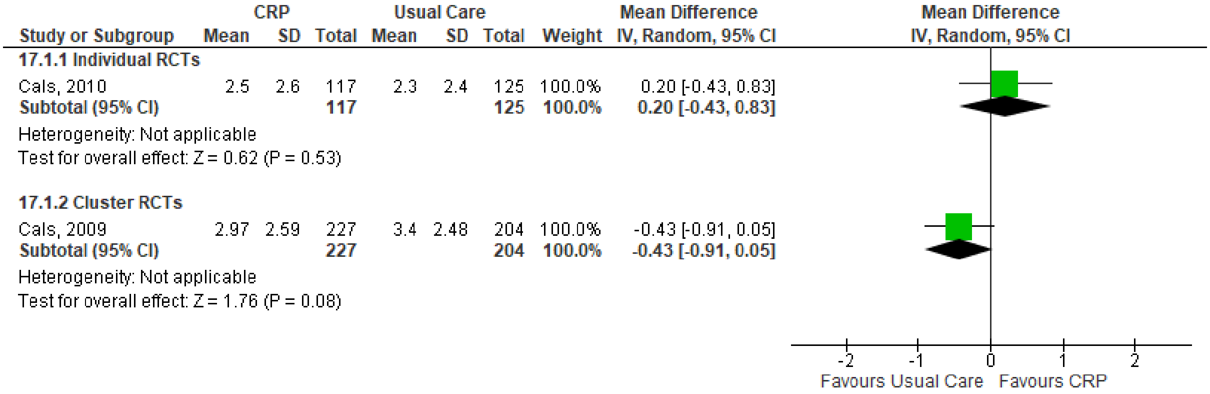

Individual trial estimates

Appendix, Table 4. Individual trial estimates from data not combined in meta-analyses.

Study, year	Outcome	End point	CRP n/N or Mean(SD)/N	Usual Care n/N or Mean(SD)/N	RR (95% CI)	P
**Clinical recovery and resolution of symptoms**						
Cals, 2010	Clinical recovery from URTI	7 days	12 / 51	9 / 49	0.94 (0.77 to 1.15)	0.53
Cals, 2010	Clinical recovery from LRTI	7 days	15 / 67	22 / 76	1.09 (0.90 to 1.32)	0.37
Cals, 2010	Time to full clinical recovery from Rhinosinusitis, mean number of days	28 days	17.3 (SD 9.3) / 67	16.6 (SD 9.9) / 76	0.70 (-2.45 to 3.85)	0.66
Cals, 2010	Time to full clinical recovery from LRTI, mean number of days	28 days	17.5 (SD 9.2) / 51	19.8 (SD 9.5) / 49	-2.30 (-5.97 to 1.37)	0.22
Little, 2013	Mean symptom severity scores	2-4 days	1.7 (SD 1.0) / 760	1.75 (SD 0.95) / 861	-0.05 (-0.15 to 0.05)	0.30
**Visit duration and visits at follow-up**						
Gonzales, 2011	Visit duration, minutes	Index consultation	282.0 (SD 123.0) / 69	287.0 (SD 122.0) / 62	-5.00 (-47.01 to 37.01)	0.82
Cals, 2009	Visits due to RTI during follow-up, per patients per year	3.5 years (starting after first 28 days)	0.4 (SD 0.51) / 203	0.56 (SD 0.85) / 176	-0.16 (-0.30 to -0.02)	0.03
**Referrals to secondary care**						
Schot, 2018	Non-urgent referrals to secondary care (specialists or ED) due to the same episodes	at the time of re-consultations	3 / 81	5 / 99	0.73 (0.18 to 2.98)	0.66
Schot, 2018	Non-urgent referrals to secondary care (specialists or ED) due to new episodes	3 months	3 / 81	7 / 99	0.52 (0.14 to 1.96)	0.34
**Admissions to hospital and mortality**						
Cals, 2009	Hospital admissions as adverse events due to RTI	3.5 years	3 / 203	2 / 176	1.30 (0.22 to 7.69)	0.77
Cals, 2010	Hospitalisations as serious adverse events	at consultation time	0 / 129	0 / 129		
Cals, 2010	Deaths as serious adverse events	at consultation time	0 / 129	0 / 129		

Note. URTI, Upper Respiratory Tract Infection; LRTI, Lower Respiratory Tract Infection; N, total number of patients; n, number of patients or events; RR, Relative Risk; CI, confidence interval; Mean, weighted mean difference; SD, standard deviation; P, overall p-value.

Sensitivity analyses

Appendix, Table 5. Sensitivity analyses.

**Outcome**	**RCTs, n**	**RR(95%CI)**	**CRP group (Events / Total), N**	**Usual Care (Events / Total), N**	**Test for overall effect, Z**	**Heterogeneity**
**Antibiotic prescribing at the index consultation**						
Overall original analysis	13	0.79 (0.70 to 0.90)	1289 / 3377	1720 / 3347	3.59 (P = 0.0003)	$Tau^2 = 0.03$; $Chi^2 = 49.59$, df = 12 (P < 0.00001); $I^2 = 76\%$
**Sensitivity analyses**						
*Excluding one RCT that excluded patients with severe RTI [1], and one RCT that did not report exclusion criteria [2]	11	0.84 (0.71 to 0.99)	787 / 2254	951 / 2194	2.08 (P = 0.04)	$Tau^2 = 0.05$; $Chi^2 = 40.79$, df = 10 (P<0.0001); $I^2 = 75\%$
†Excluding one RCT: patients at high risk of serious infections were excluded post-hoc [3]	12	0.78 (0.68 to 0.89)	1172 / 2922	1614 / 2966	3.69 (P = 0.0002)	$Tau^2 = 0.03$; $Chi^2 = 44.80$, df = 11 (P < 0.00001); $I^2 = 75\%$
‡Excluding one cluster RCT with factorial design [4]	12	0.80 (0.71 to 0.92)	1269 / 3312	1689 / 3288	3.23 (P = 0.001)	$Tau^2 = 0.03$; $Chi^2 = 48.67$, df = 11 (P < 0.00001); $I^2 = 77\%$
§Excluding 1 RCT with different CRP analyser [2]	11	0.81 (0.71 to 0.93)	1228 / 3271	1598 / 3213	2.92 (P = 0.003)	$Tau^2 = 0.04$; $Chi^2 = 46.55$, df = 11 (P < 0.00001); $I^2 = 76\%$
RCTs in Europe	10	0.82 (0.69 to 0.97)	762 / 2185	932 / 2132	2.31 (P = 0.02)	$Tau^2 = 0.05$; $Chi^2 = 37.94$, df = 9 (P < 0.0001); $I^2 = 76\%$
RCTs in other continents: Japan, Vietnam, USA	3	0.71 (0.59 to 0.85)	527 / 1192	788 / 1215	3.69 (P = 0.0002)	$Tau^2 = 0.02$; $Chi^2 = 5.71$, df = 2 (P = 0.06); $I^2 = 65\%$

Note.

* Both RCTs had high prescribing rates in the usual care group and were carried out in the outpatient services (hospital & health centres) in Vietnam and Japan. [1, 2].

† RCT in patients with acute infection, carried out in general practices in Belgium [3].

‡ RCT carried out in general practices in the Netherlands. Data reported for a 2x2 factorial analysis of combined interventions built based on four individual intervention arms for which data were not reported. Individual intervention arms: CRP, Usual Care, Communication Skills (CST), Combined (CRP + CST). Factorial comparison used in meta-analysis: CRP (CRP + Combined [CRP+CST]) versus No CRP (Usual Care + CST). Both on its own or in combination with CST, CRP POCT reduced prescribing [4].

§ RCT carried out in Japan, using a CRP multichannel analyser that allowed a turnaround of CRP results of 40-50 minutes [2].

Funnel Plot and Egger's Test

Appendix, Figure 15. Funnel plot and Egger Test to assess publication bias.

Outcome: overall antibiotic prescribing at index consultations. Funnel plot drawn using R software.

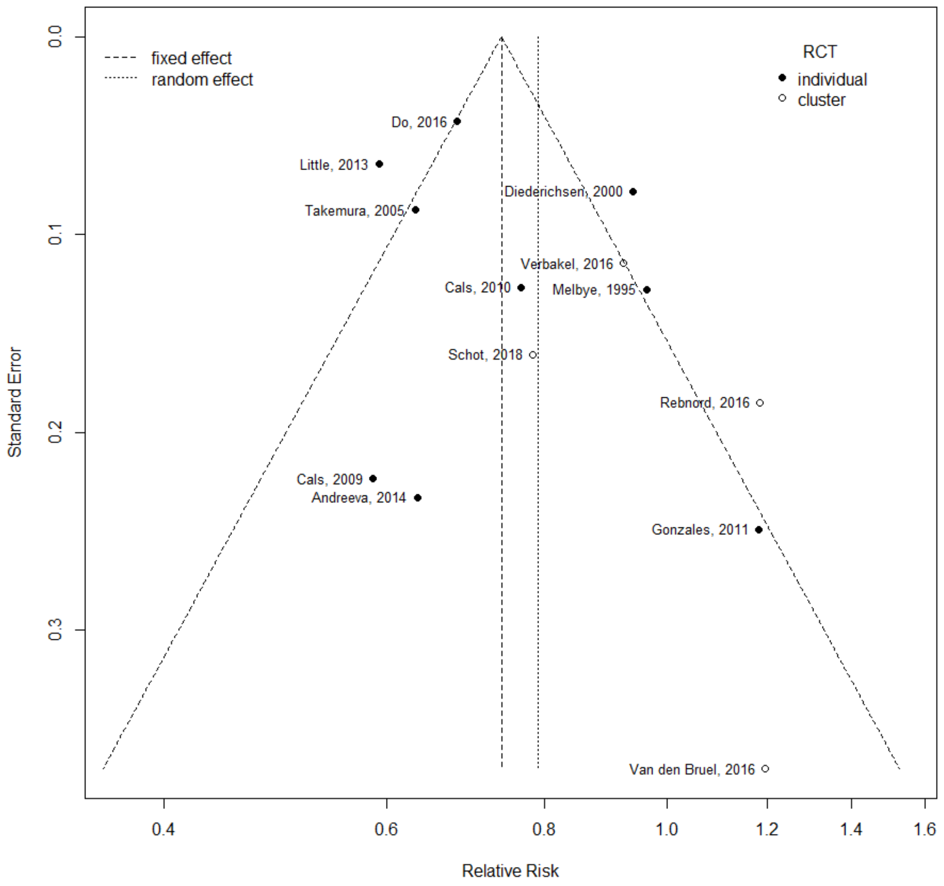

Egger's Test

```
eggers.test(x = a002a0_01)
###                 Intercept ConfidenceInterval     t       p
### Egger's test      1.783        -0.177-3.743 1.847 0.09177
```

Meta-regression

Appendix, Table 6. Meta-regression results for the outcome of overall antibiotic prescribing at index consultations.

Meta-analysis of 15 studies split according to age groups*

I2 (residual heterogeneity / unaccounted variability):	74.4%
τ2 (estimated amount of residual heterogeneity):	0.0333
Test for Residual Heterogeneity:	<0.001

Meta-regression of the 15 studies split according to age groups*

Model: ~ year + age group + crp guidance		
Reference: year = 2010; age group = adults; crp guidance = no		
year is centered with mean 2010		
	**REML**	**ML**
I^2 (residual heterogeneity / unaccounted variability):	47.9%	17.9%
τ^2 (estimated amount of residual heterogeneity):	0.1169	0.0571
Test for Residual Heterogeneity:	0.032	0.032
R^2 (amount of heterogeneity accounted for):	59.0%	88.9%
Test of Moderators (omnibus test):	0.013	<0.001

Note: year (centred with the mean of publication year = 2010); CI = Confidence Intervals; REML, Restricted Maximum Likelihood; ML, Maximum Likelihood.

*Studies that reported mutually exclusive data by age groups (adults and children) were included as separate studies in the analyses.

Meta-regression results (REML)

	Estimate	SE	z-Value	CI lower	CI upper	P-Value	RR	95% CI
**Intercept**	-0.195	0.124	-1.573	-0.438	0.0480	0.116	0.823	(0.645,1.049)
**Year (centered with 2010)**	-0.016	0.008	-1.899	-0.032	0.0005	0.058	0.985	(0.969,1.001)
**Year 2020 vs 2010**	-0.156	0.008	-1.899	-0.316	0.005	0.058	0.856	(0.729,1.005)
**Age group**								
Adults	0.000							
Children	0.207	0.121	1.703	-0.031	0.4441	0.089	1.229	(0.969,1.559)
Children & Adults	-0.342	0.184	-1.861	-0.702	0.0183	0.063	0.711	(0.496,1.018)
**CRP guidance**								
no	0.000							
yes	-0.147	0.124	-1.183	-0.391	0.0966	0.237	0.863	(0.677,1.101)

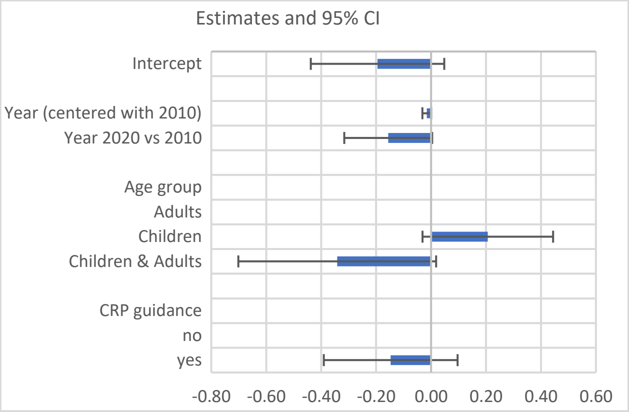

Meta-regression results (ML)

	Estimate	SE	z-Value	CI lower	CI upper	P-Value	RR	95% CI
**Intercept**	-0.201	0.093	-2.174	-0.382	-0.0198	0.030	0.818	(0.682,0.980)
**Year (centered with 2010)**	-0.016	0.006	-2.451	-0.028	-0.0032	0.014	0.984	(0.972,0.997)
**Year 2020 vs 2010**	-0.157	0.006	-2.451	-0.283	-0.032	0.014	0.855	(0.754,0.969)
**Age group**								
Adults	0.000							
Children	0.190	0.086	2.226	0.023	0.3581	0.026	1.210	(1.023,1.431)
Children & Adults	-0.337	0.133	-2.532	-0.597	-0.0760	0.011	0.714	(0.550,0.927)
**CRP guidance**								
no	0.000							
yes	-0.156	0.097	-1.610	-0.346	0.0340	0.108	0.856	(0.708,1.035)

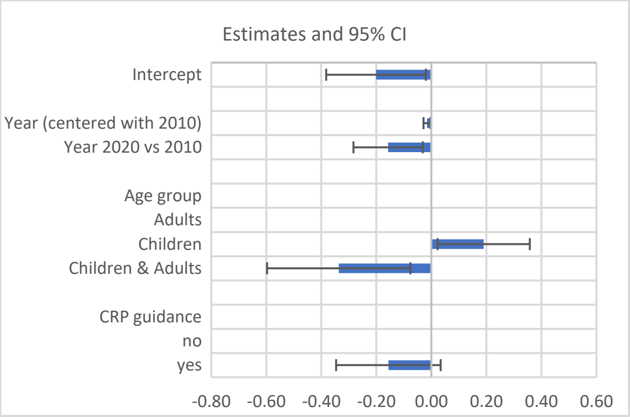

Note: year (centred with the mean of publication year = 2010); CI = Confidence Intervals; REML, Restricted Maximum Likelihood; ML, Maximum Likelihood.

Appendix, Figure 16. Funnel plots for meta-analysis and meta-regression considering studies split according to age.

a) Meta-analysis of 15 studies split according to age groups*

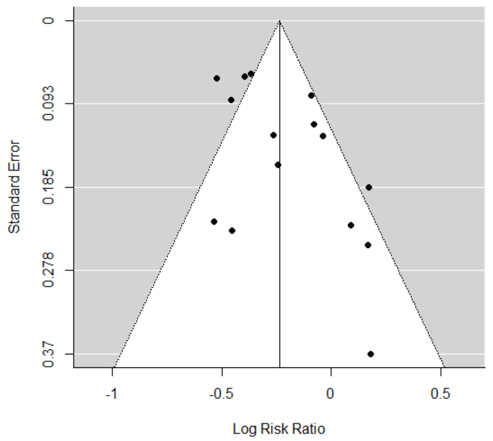

* studies with mutually exclusive data by age groups (adults and children) were included as separate studies

Egger's Test

```
> regtest(a003a1_00, model="rma", predictor="sei")

 Regression Test for Funnel Plot Asymmetry

 model:     mixed-effects meta-regression model
 predictor: standard error

 test for funnel plot asymmetry: z = 2.2362, p = 0.0253
```

b) Meta-regression of 15 studies split according to age groups after corrected for modifiers of between-study heterogeneity

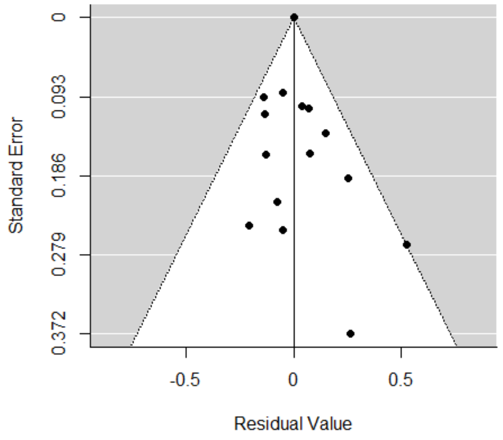

* modifiers: year (centred with the mean of publication year = 2010), CRP cut-off guidance (no/yes), age groups (adults, adults and children, children)

Egger's Test

```
> regtest(a003a2_03, model="rma", predictor="sei")

Regression Test for Funnel Plot Asymmetry

model:     mixed-effects meta-regression model
predictor: standard error

test for funnel plot asymmetry: z = 1.5862, p = 0.1127
```

References

1. Do NT, Ta NT, Tran NT, Than HM, Vu BT, Hoang LB, et al. Point-of-care C-reactive protein testing to reduce inappropriate use of antibiotics for non-severe acute respiratory infections in Vietnamese primary health care: a randomised controlled trial. The Lancet Global health. 2016;4(9):e633-41.
2. Takemura Y, Ebisawa K, Kakoi H, Saitoh H, Kure H, Ishida H, et al. Antibiotic selection patterns in acutely febrile new outpatients with or without immediate testing for C reactive protein and leucocyte count. Journal of clinical pathology. 2005;58(7):729-33.
3. Verbakel JY, Lemiengre MB, De Burghgraeve T, De Sutter A, Aertgeerts B, Shinkins B, et al. Should all acutely ill children in primary care be tested with point-of-care CRP: a cluster randomised trial. BMC Med. 2016;14(1):131.
4. Cals JW, Butler CC, Hopstaken RM, Hood K, Dinant GJ. Effect of point of care testing for C reactive protein and training in communication skills on antibiotic use in lower respiratory tract infections: cluster randomised trial. BMJ. 2009;338:b1374.
